# Common mental disorders in young adults: temporal trends in primary care episodes and self-reported symptoms

**DOI:** 10.1136/bmjment-2024-301457

**Published:** 2025-05-13

**Authors:** Jennifer Dykxhoorn, Francesca Solmi, Kate Walters, Shamini Gnani, Antonio Lazzarino, Judi Kidger, James B Kirkbride, David P J Osborn

**Affiliations:** 1 UCL Division of Psychiatry, London, UK; 2 UCL Research Department of Primary Care and Population Health, London, UK; 3 Department of Primary Care and Public Health, Imperial College London, London, UK; 4 Population Health Sciences, University of Bristol, Bristol, UK

**Keywords:** Depression, Anxiety disorders, Child & adolescent psychiatry, Adult psychiatry

## Abstract

**Background:**

Rates of common mental disorders (CMDs) including anxiety, depression and stress, treated in primary care have increased among young adults, but it is unclear if this reflects more help-seeking and/or an increase in symptoms, and if there are differences across sociodemographic groups.

**Objective:**

This study examined trends in primary care-recorded CMD and self-reported psychological distress symptoms in young adults over time.

**Methods:**

We used data from participants born between 1980 and 2003 in two datasets: UK primary care records and longitudinal cohort data. Participants were followed from age 16 to age 39 (maximum) or the end of the follow-up (2019–2020). Annual incidence rates of recorded CMD overall and by sociodemographic group were calculated for 2009–2019, using incidence rate ratios to explore changes. We calculated annual self-reported psychological distress symptoms from cohort data, calculating ratios to explore changes over time.

**Findings:**

Between 2009 and 2019, recorded CMD increased by 9.90%, while average psychological distress symptoms rose by 19.33%. The sharpest increases for both recorded CMD and average psychological distress symptoms were observed in older adolescents (ages 16–19) and those born after 1995. Recorded CMD increased more in males (20.61%) than in females (7.65%), despite similar symptom increases. Recorded CMD increased the most in the least deprived areas (16.34%) compared with the most deprived areas (3.55%), despite comparable rises in psychological distress symptoms.

**Conclusions:**

Both recorded CMD and psychological distress symptoms in young adults increased between 2009 and 2019, which may indicate that the rising primary care-recorded CMD reflects increased symptom burden.

**Implications:**

Differences between recorded CMD and psychological distress symptoms across sociodemographic groups highlight potential misalignment in mental healthcare with underlying population need, indicating that the most affected groups may not be those receiving the most care.

**WHAT IS ALREADY KNOWN ON THIS TOPIC**

- The observed rise in common mental disorders (CMD) among young people has been a prominent concern in public and professional discourse, with potential drivers of these trends—including increased psychological distress, greater help-seeking, or changing primary care practices— remaining insufficiently explored.
- This study utilised electronic health records and population cohort data to examine temporal patterns and sociodemographic variation in primary care-recorded common mental disorders and self-reported psychological distress, aiming to explore underlying mechanisms.
- We performed searches in MEDLINE, PubMed and Google Scholar to identify studies published in English in the last 10 years using search terms relevant to (1) common mental disorders, including symptoms, diagnoses or pharmaceutical treatment for anxiety, depression or psychological distress; (2) young adults, including adolescents and young adults; and (3) population rates over time, including incidence, prevalence, temporal trends or secular patterns.

**WHAT THIS STUDY ADDS**

- We found an increase in both primary care-recorded CMD and self-reported psychological distress symptoms in young adults between 2009 and 2019. We observed a larger increase in self-reported psychological distress symptoms than primary care-recorded CMD. If the observed increase in self-reported symptoms reflects a growing burden of mental health problems, these differences may indicate that the increases in mental healthcare may not be keeping pace with the underlying increases in self-reported symptoms. We revealed important differences in the patterns of CMD in the two data sources, revealing inequalities across sociodemographic groups.

**HOW THIS STUDY MIGHT AFFECT RESEARCH, PRACTICE OR POLICY**

- Our study adds to the growing evidence of increasing mental health problems in young adults in the UK. We showed that the symptoms of common mental disorders were increasing more than the observed increases in mental healthcare, suggesting that service provision has not kept pace with underlying need for mental health support. Importantly, the differences we observed in patterns of psychological distress symptoms and CMD recorded in primary care reveal growing inequalities across sociodemographic groups, where the population groups who experienced the largest increases in psychological distress symptoms did not correspond to the groups with the largest increases in mental healthcare. For example, those living in the most deprived areas had the largest increases in psychological distress symptoms but the smallest increases in primary care-recorded CMD, when compared with those living in more affluent areas. These findings suggest that current mental health service provision is not aligned to the populations with the greatest burden of mental health problems, exacerbating systemic inequalities. By triangulating the evidence, this study provides key information to inform equitable treatment provision to better support the mental health of young adults.

## Background

 Common mental disorders (CMD), including anxiety, depression and stress, are leading causes of years lived with disability, particularly for young adults aged 15–24. In this age group, depressive and anxiety disorders are the second-leading and fourth-leading causes of years lived with disability, respectively.^1^

Rates of primary care-recorded CMD have increased in the UK since 2000,^2^ but this increase could be attributed to several mechanisms. For instance, symptoms of CMD may have become more common,^3^ young people may seek help more often due to increased mental health literacy and decreased stigma,^4^ or primary care practitioners may have changed approaches to CMD identification, recording and treatment, including increased recording of symptoms and decreased use of diagnostic labels over time.^5^ These different mechanisms might also coexist in the population, and rates of CMD may be differentially impacted by these mechanisms across age, sex, ethnic or regional characteristics. Triangulating electronic health record data and population cohort data could help understand these mechanisms and inform population-based prevention and treatment provision to better support the mental health of young adults.

### Objectives

We used primary healthcare records and longitudinal cohort data from the UK to investigate whether (1) the increase in primary care-recorded CMD corresponded to an increase in self-reported psychological distress symptoms in young adults, and (2) these patterns differed by sex, age, cohort, ethnicity, country, region and deprivation. We compared the patterns in these two datasets to understand the drivers of increased CMD in young adults with the aim of informing population-based prevention and treatment provision.

## Methods

### Data sources

We used two datasets: electronic primary care records from the *Clinical Research Practice Datalink (CPRD)*; and longitudinal cohort data from *Understanding Society (USoc)*. The analytical protocol was preregistered (https://osf.io/xr3bc). Analyses were conducted in Stata and R.

### CPRD methods

#### Study sample

We included participants born between 1980 and 2003 registered with a UK primary care practice for at least 12 months between 2009 and 2019 (online supplemental A).

#### Measures

The primary outcome was annual incidence of primary care-recorded CMD (referred to as ‘recorded CMD’), including (1) symptoms or diagnoses for anxiety, depression and/or stress (CMD), or (2) pharmaceutical treatment for CMD including antidepressants or anxiolytics, consistent with previous research (online supplemental B).^2^ To calculate the incidence of new episodes, participants who were receiving ongoing care for CMD within primary care were excluded from annual estimates. New episodes of CMD included any participant who was diagnosed or treated for CMD, and they had not been diagnosed and/or treated for CMD in the previous 12 months.

We stratified estimates by sociodemographic group including sex (female, male), age group (ages 16–19, 20–24, 25–29, 30–34, 35–39), cohort (birth years: 1980–1984, 1985–1989, 1990–1994, 1995–1999, 2000–2003), ethnicity (Asian, Black, Mixed, Other, White, Not stated) and country (England, Scotland, Northern Ireland, Wales). Within England, we stratified by region (North East, North West, Yorkshire and the Humber, East Midlands, West Midlands, East of England, London, South East, South West) and deprivation.

#### Missing data

We had complete data on all characteristics apart from ethnicity. Participants without recorded ethnicity were included in a ‘Not stated’ ethnic group.

#### Statistical analysis

Participants entered the cohort on 1 January 2009 if they were aged 16 or older, or the year they turned 16 if after 2009. We estimated the annual incidence per 1000 person-years (PYs) and 95% CIs of recorded CMD overall. We fit multilevel Cox regression, clustered by participant, and used Wald tests to determine the presence of interactions across sociodemographic strata and time. We reported stratified incidence rate ratios (IRR) and 95% CI comparing the incidence in 2009 (study start) to 2014 (midpoint); 2014 to 2019 (study end); and overall (2009 to 2019) (online supplemental C).

### USoc methods

#### Study sample

We included participants born from 1980 to 2003 who participated in at least one wave of USoc between 2009–2010 and 2019–2020 (online supplemental A). Participants were included in 2009, or after their 16th birthday, if later than 2009.

#### Measures

Our primary outcome was self-reported psychological distress symptoms (referred to throughout as ‘psychological distress symptoms’) measured by the 12-item General Health Questionnaire (GHQ-12), a self-report questionnaire measuring symptoms of psychological distress, depression and anxiety in population studies.^6^ Participants receive a score between 0 (no psychological distress symptoms) to 36 (high psychological distress symptoms) (online supplemental A). We conducted a post-hoc analysis where psychological distress symptoms were dichotomised using the threshold of a score of ≥14 indicating high psychological distress.

#### Missing data

We explored patterns of missing variables, comparing the full sample with those who had complete data. We generated 50 imputed datasets for missing data, combined using Rubin’s rules, with the assumption that data were missing at random (online supplemental A).

#### Statistical analysis

We used cross-sectional weights to generate representative estimates by accounting for unequal selection probability and differential non-response.^7^ CMD symptom scores were calculated annually, generating separate estimates for each study wave (eg, 2009–2010; 2010–2011). We fit multilevel linear regression models with CMD symptom scores as the dependent variable, clustered by participant to account for repeated measures. To assess whether the association between time and CMD symptom scores varied by sociodemographic factors, we included interaction terms between time and each sociodemographic variable, performing likelihood ratio tests (LRT) to determine if the interaction improved model fit. To estimate the relative change in CMD symptom scores over time, we calculated rate ratios (RR) and 95% CI, comparing the CMD symptom scores at the start of the study (2009–2010) to the midpoint (2014–2015) and to the end of follow-up (2019–2020). These RRs represent multiplicative differences in CMD symptom scores over time. We calculated the ratio of CMD symptom scores within each sociodemographic category, using the Delta method to estimate the variance. In the post-hoc analysis, we modelled the weighted proportion and 95% CI of those exceeding the symptom threshold (GHQ-12 ≥14) in each stratum (online supplemental D).

## Findings

### Sample characteristics

There were 7 354 888 unique participants in the primary care sample contributing 26 928 036 PYs of follow-up. 54.03% were female and 87.87% resided in England. Of those with recorded ethnicity, 71.17% were from the White ethnic group, broadly consistent with censuses estimates (online supplemental A). In England, the largest proportion of participants were registered to practices in London (27.85%) and the smallest proportion in the North East (2.68%). 23.51% of participants were registered to practices in the most deprived areas in England and 13.18% were registered to practices in the least deprived fifth. We reported sample characteristics in 2009, 2014 and 2019 (table 1; online supplemental C table 1).

**Table 1:** Primary care (Clinical Research Practice Datalink) sample characteristics: 2009, 2014 and 2019

	2009		2014		2019	
	n	%	n	%	n	%
Total	2 179 380	100.00	3 061 418	100.00	3 935 301	100.00
Sex						
Female	1 144 514	52.50	1 614 301	52.73	2 034 723	51.70
Male	1 034 866	47.50	1 447 117	47.27	1 900 578	48.30
Age group						
16–19	169 508	7.78	163 530	5.34	158 905	4.04
20–24	702 825	32.25	691 587	22.59	680 305	17.29
25–29	1 307 047	59.97	925 879	30.24	877 798	22.31
30–34	–	–	1 280 422	41.82	1 018 290	25.88
35–39	–	–	–	–	1 200 003	30.49
Cohort						
1980–1984	1 328 153	60.94	1 304 076	42.60	1 219 774	31.00
1985–1989	687 754	31.56	921 962	30.12	1 016 325	25.83
1990–1994	163 473	7.50	678 220	22.15	879 112	22.34
1995–1999	–	–	157 160	5.13	668 104	16.98
2000–2003	–	–	–	–	151 986	3.86
Ethnicity						
Asian	192 098	13.92	340 259	15.76	461 591	16.10
Black	76 413	5.54	119 025	5.50	160 523	5.60
Mixed	51 072	3.70	88 510	4.10	139 285	4.86
Other	26 924	1.95	47 058	2.20	76 870	2.68
White	1 033 148	74.88	1 564 471	72.45	2 028 898	70.76
Country						
England	1 882 792	86.39	2 649 545	86.55	3 413 569	86.74
Scotland	162 414	7.45	230 273	7.52	297 645	7.56
Northern Ireland	31 305	1.44	40 106	1.31	50 927	1.29
Wales	102 869	4.72	141 494	4.62	173 160	4.40
Region*						
North East	54 434	2.50	76 163	2.49	92 089	2.34
North West	305 487	14.02	406 230	13.27	541 028	13.75
Yorkshire and the Humber	84 607	3.88	108 575	3.55	126 587	3.22
East Midlands	85 335	3.92	92 306	3.02	117 900	3.00
West Midlands	240 884	11.05	344 809	11.26	445 681	11.33
East of England	83 903	3.85	98 123	3.21	103 118	2.62
London	431 105	19.78	711 831	23.25	973 897	24.75
South East	380 293	17.45	521 410	17.03	659 028	16.75
South West	216 744	9.95	290 098	9.48	354 241	9.00
Deprivation*						
Fifth 1 (least deprived)	273 508	12.55	401 528	13.12	542 754	13.79
Fifth 2	395 052	18.13	554 444	18.11	716 215	18.20
Fifth 3	401 383	18.42	557 730	18.22	705 587	17.93
Fifth 4	573 390	26.31	804 201	26.27	1 029 073	26.15
Fifth (most deprived)	536 047	24.60	743 515	24.29	941 672	23.93

* England only.

We included 25 214 unique participants in the USoc sample, 53.11% were female, 68.94% were from the White ethnic group and 81.68% resided in England. We reported sample characteristics in 2009, 2014 and 2019 (table 2; online supplemental D table 1).

**Table 2:** Understanding Society sample characteristics of full sample, 2009–2010; 2014–2015; and 2019–2020

	2009–2010 (wave A)		2014–2015 (wave F)		2019–2020 (wave K)	
	n	%	n	%	n	%
Total	10 245	100.00	10 668	100.00	7081	100.00
Sex						
Female	5730	55.90	5874	55.06	3984	56.26
Male	4515	44.10	4794	44.94	3097	43.74
Age group						
16–19	2936	28.66	2419	22.68	1337	18.88
20–24	3397	33.16	2743	25.71	1506	21.27
25–29	3679	35.91	2432	22.80	1285	18.15
30–34	233	2.27	2835	26.57	1311	18.51
35–39	–	–	239	2.24	1642	23.19
Cohort						
1980–1984	3899	38.10	3000	28.12	1650	23.30
1985–1989	3400	33.20	2467	23.13	1301	18.37
1990–1994	2946	28.80	2726	25.55	1284	18.13
1995–1999	–	–	2475	23.20	1515	21.40
2000–2003	–	–			1331	18.80
Ethnicity						
Asian	1927	18.81	1869	17.52	1152	16.27
Black	724	7.07	787	7.38	256	3.62
Mixed	354	3.46	369	3.46	243	3.43
Other	155	1.51	153	1.43	54	0.76
White	7084	69.15	7460	69.93	5363	75.74
Missing	1	0.01	30	0.28	13	0.18
Country						
England	8767	85.57	8741	81.94	5742	81.09
Scotland	638	6.23	693	5.84	470	5.66
Northern Ireland	368	3.59	611	5.73	468	6.61
Wales	472	4.61	623	5.84	401	5.66
Region*						
North East	427	4.87	359	4.11	218	3.80
North West	1092	12.46	1128	12.90	802	13.97
Yorkshire and the Humber	918	10.47	977	11.18	639	11.13
East Midlands	765	8.73	750	8.58	512	8.92
West Midlands	936	10.68	978	11.19	660	11.49
East of England	797	9.09	757	8.66	609	10.61
London	2127	24.26	2030	23.22	1036	18.04
South East	1066	12.16	1074	12.29	770	13.41
South West	639	7.29	688	7.87	496	8.64
Deprivation*						
Fifth 1 (least deprived)	2781	31.72	1074	12.29	943	16.42
Fifth 2	1932	22.04	1372	15.70	1072	18.67
Fifth 3	1448	16.52	1489	17.03	1072	18.67
Fifth 4	1250	14.26	2003	22.91	1254	21.84
Fifth 5 (most deprived)	946	10.79	2803	32.07	1401	24.40
Missing	410	4.68	422	4.83	422	7.35

* England only.

### Patterns of recorded CMD and psychological distress symptoms

**Figure 1:**
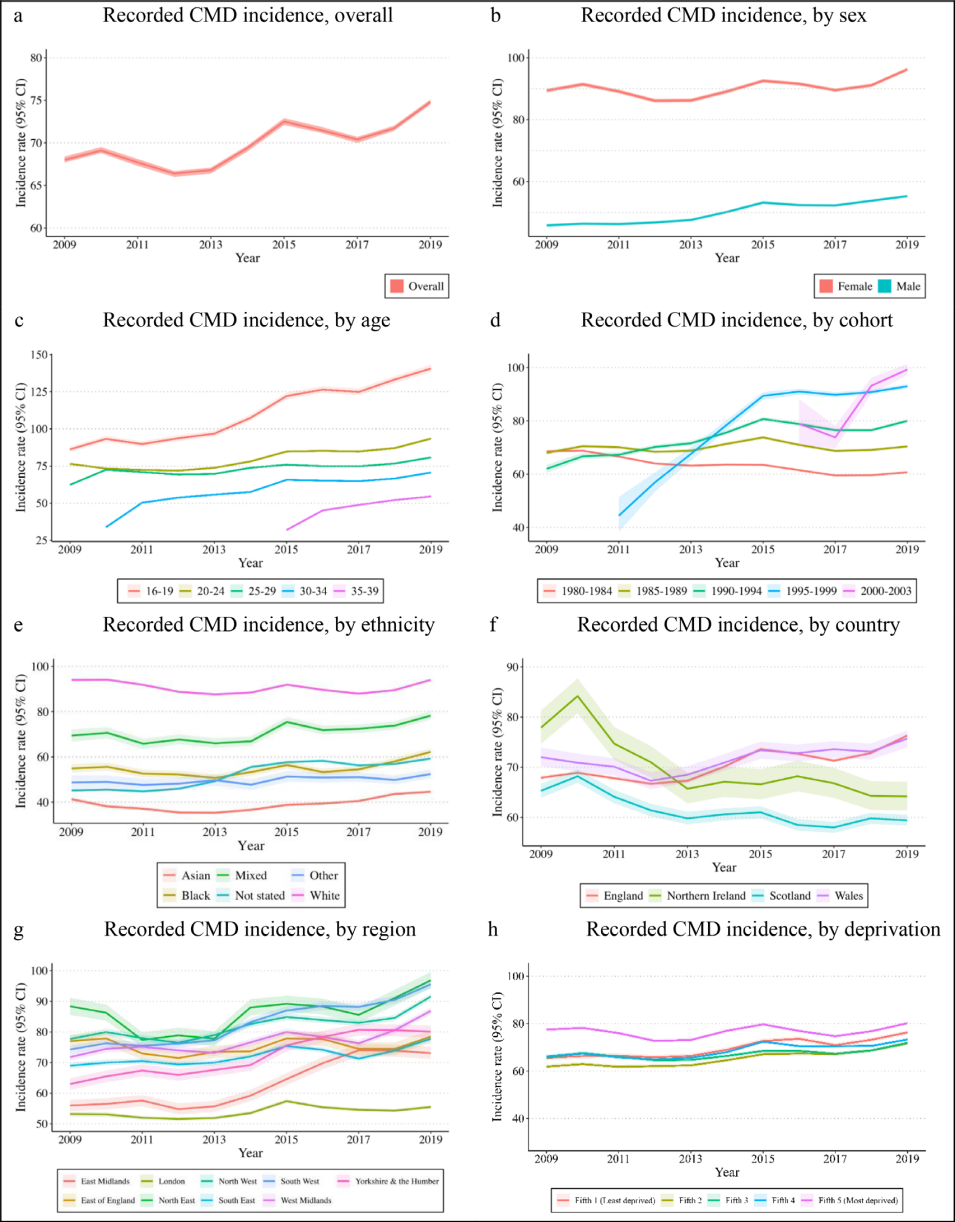
Primary care-recorded CMD incidence (per 1000 person-years) and 95% CI, overall and by sociodemographic group (CPRD). Notes: 95% CIs indicated by shaded areas, Y-axis scales differ between figures in order to clearly display the results. CMD, common mental disorder; CPRD, Clinical Research Practice Datalink.

The annual incidence of recorded CMD (per 1000 PYs) increased from 68.05 (95% CI 67.66 to 68.44) in 2009 to 74.79 (95% CI 74.45 to 75.13) in 2019, a 9.90% increase (95% CI 9.11% to 10.70%; figure 1; online supplemental C table 2) We found evidence of interactions across all sociodemographic characteristics (Wald>0.001) and reported stratified estimates (online supplemental C table 3).

**Figure 2:**
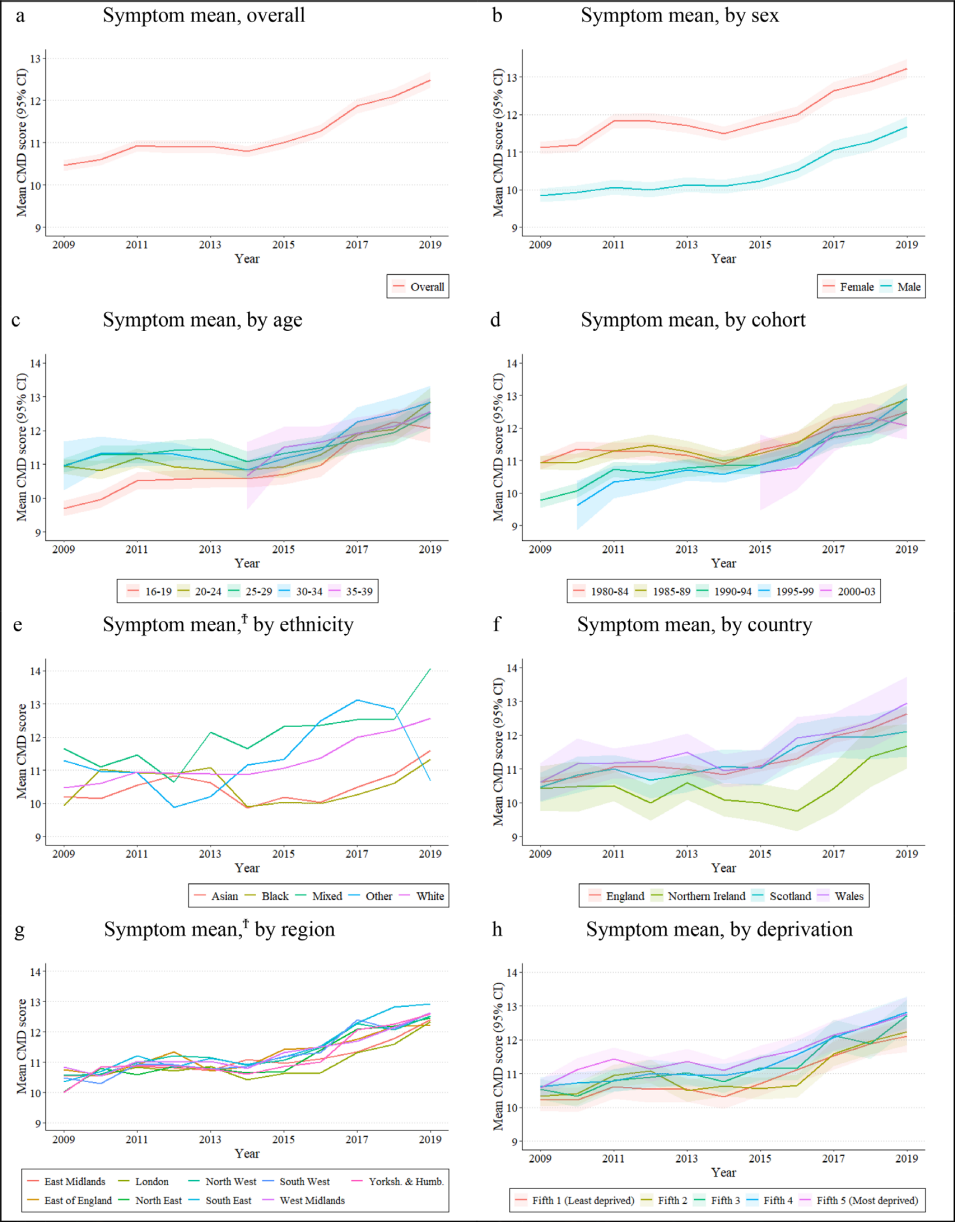
Self-reported psychological distress symptoms, mean and 95% CI, overall and by sociodemographic group (USoc). Notes: 95% CIs indicated by shaded areas, Y-axis scales differ between figures in order to clearly display the results. ^Ϯ^CIs removed as they overlap. CMD, common mental disorder; USoc, Understanding Society.

Self-reported psychological distress symptoms increased by 19.34% (95% CI 17.05% to 21.62%) between 2009–2010 and 2019–2020 (figure 2; online supplemental D table 2). We found evidence of interactions by age, cohort, ethnicity and region (LRT>0.001), but not by sex, country or deprivation (online supplemental D table 3).

### Sex

In 2009, the incidence (per 1000 PYs) was higher in females (89.39, 95% CI 88.77 to 90.01) than males (45.86, 95% CI 45.40 to 46.31). Females continued to have a higher incidence of recorded CMD across all years; we saw larger relative increases in recorded CMD among males (20.60%; 95% CI 19.13% to 22.10%) than females (7.65%; 95% CI 6.68% to 8.63%) (figure 1).

Females had higher psychological distress symptom scores than males in 2009–2010 (females 11.12, 95% CI 10.96 to 11.28; males: 9.85, 95% CI 9.67 to 10.03), which persisted across the study period. We did not find evidence of an interaction for sex over time (LRT=0.55), evidenced by similar patterns of increase (figure 2).

### Age

The incidence of recorded CMD was highest in those aged 16–19 (86.18, 95% CI 84.40 to 88.01) compared with those aged 20–24 (76.49, 95% CI 75.75 to 77.24) or aged 25–29 (62.32, 95% CI 61.86 to 62.79) in 2009. The most pronounced relative increase was observed in the youngest age group (aged 16–19), increasing by 63.30% (95% CI 58.92% to 67.82%); 22.26% (95% CI 20.61% to 23.94%) in those aged 20–24; and 29.63% (95% CI 28.11% to 31.16%) in those aged 25–29.

Those aged 16–19 had the lowest level of psychological distress symptoms in 2009–2010 but experienced the largest relative increase over time (24.22%, 95% CI 19.28% to 29.16%). However, this estimate overlapped with the increases observed in other age groups.

### Cohort

We observed an 11.49% decrease in recorded CMD between 2009 and 2019 among those born between 1980 and 1984 (online supplemental C table 3). The incidence of recorded CMD increased over time in all other cohorts, with more pronounced increases for the 1990–1994 cohort (29.05%, 95% CI 25.68% to 32.53%), and the 1995–1999 cohort (109.46%, 95% CI 80.94% to 143.84%). We observed a 25.84% relative increase (95% CI 12.54% to 41.16%) in recorded CMD for the 2000–2003 cohort between 2016 and 2019.

Psychological distress symptoms increased for all cohorts, with the largest relative increases observed in later-born cohorts: 27.61% (95% CI 22.41% to 32.81%) in the 1990–1994 cohort; and 33.33% (95% CI 22.47% to 44.18%) in the 1995–1999 cohort, compared with 15.88% (95% CI 11.52% to 20.24%) in the earliest-born cohort (1980–1984) (online supplemental D table 3).

### Ethnicity

The highest incidence of recorded CMD in 2009 was in the White ethnic group (incidence per 1000 PYs: 94.00; 95% CI 93.32 to 94.68), which did not change over the study period. Incidence was lower in all other ethnic groups at baseline but increased over time. Recorded CMD incidence increased by 13.18% (95% CI 8.57% to 18.01%) in the Black group, 12.77% (95% CI 7.86% to 17.93%) in the Mixed group, and 7.97% (95% CI 4.78% to 11.28%) in the Asian group. These distinct patterns in the White ethnic group and minoritised ethnic groups contributed to converging rates over time.

At baseline, psychological distress was highest in the Mixed ethnic group compared with Black, Asian and White groups. Psychological distress symptoms increased over time in the Mixed (20.78%, 95% CI 6.93% to 34.63%), White (19.88%, 95% CI 17.38% to 22.37%), Black (14.08%, 95% CI 1.00% to 28.24%) and Asian (13.56%, 95% CI 7.34% to 19.77%) groups, but not in the Other ethnic group.

### Country

In 2009, the recorded CMD incidence was highest in Northern Ireland (77.92, 95% CI 74.68 to 81.31 per 1000 PYs), and lowest in Scotland (65.29, 95% CI 63.92 to 66.69 per 1000 PYs). Incidence decreased by 17.62% (95% CI 12.40% to 22.54%) in Northern Ireland, and 8.97% (95% CI 6.39% to 11.48%) in Scotland, but increased in England (12.35%, 95% CI 11.48% to 13.23%) and Wales (5.03%, 95% CI 1.59% to 8.60%).

We observed similar psychological distress symptoms in all countries at baseline and no evidence of an interaction over time (LRT=0.05), with symptoms increasing in all countries over time.

### Region

In 2009, the incidence of recorded CMD (per 1000 PYs) was lowest in London (53.22, 95% CI 52.45 to 54.00), and highest in the North East (88.37, 95% CI 85.62 to 91.22; online supplemental C table 2). The incidence increased in all regions over time, except the East of England (IRR 1.02, 95% CI 0.98 to 1.06; figure 1). The smallest relative increases were in London (4.27%, 95% CI 2.43% to 6.14%), and the largest increases were observed in the East Midlands (30.50%, 95% CI 25.14% to 36.10%), the South West (28.78%, 95% CI 25.96% to 31.66%) and Yorkshire and the Humber (27.22%, 95% CI 22.34% to 32.30%).

In 2009–2010, the highest mean psychological distress symptoms were in West Midlands (10.84, 95% CI 10.41 to 11.26) and the lowest in Yorkshire and the Humber (10.01, 95% CI 9.63 to 10.39) and the North East (10.02, 95% CI 9.51 to 10.53; online supplemental D table 2). Psychological distress increased in all regions; however, the wide variance around each estimate precluded detecting meaningful regional differences.

### Deprivation

We observed higher incidence recorded CMD (per 1000 PYs) in 2009 in the most deprived fifth (77.47, 95% CI 76.64 to 78.30) than the least deprived fifth (65.47, 95% CI 64.52 to 66.67; figure 1). While the incidence increased across all levels of deprivation, we saw the largest relative increase in the least deprived areas: (16.34%, 95% CI 14.00% to 18.74%) and the smallest relative increase in the most deprived fifth (3.55%, 95% CI 2.11% to 5.01%), contributing to converging rates over time (online supplemental C tables 3 and 4).

At baseline, psychological distress symptoms were elevated in the most deprived fifth than the least deprived fifth, and we observed similar increases in psychological distress symptoms across the study period, finding no evidence of an interaction (LRT=0.10; online supplemental D table 4).

### Patterns of proportion exceeding psychological distress cut-off

We conducted a post-hoc analysis using a cut-off score for psychological distress and observed similar patterns to those observed in the mean symptom scores (online supplemental D table 5).

## Conclusions

We found increases to both the incidence of primary care-recorded CMD and levels of self-reported psychological distress symptoms in young adults between 2009 and 2019.

These findings indicate that the increased levels of mental healthcare provided in primary care may reflect increased levels of self-reported psychological distress symptoms.

The increases in primary care-reported CMD are consistent with reports from the National Health Service indicating increasing demand for mental health services,^8^ and the increases in self-reported psychological distress may reflect a higher burden of symptoms but also could be an indication of increased willingness to report mental health problems due to reduced self-stigma and increased mental health literacy.^4^

Notably, psychological distress symptoms increased to a larger extent than primary care-recorded CMD (19.34% vs 9.90%). This larger increase in self-reported symptoms compared with primary care-recorded CMD may indicate that available mental health services have not been able to keep pace with increased need for mental healthcare. Alternatively, these findings could suggest that individuals with high levels of psychological distress may not be seeking mental healthcare, and differences observed across sociodemographic groups may indicate differential patterns of help-seeking behaviour or barriers to mental healthcare

Recorded CMD and psychological distress symptoms varied by sociodemographic group. Females consistently reported higher psychological distress symptoms than males, which could reflect higher symptom burden or higher willingness to discuss mental health problems. While psychological distress symptoms increased similarly in both sexes, recorded CMD increased more in males (20.61%) compared with females (7.65%). This increase in recorded CMD among males may indicate increased willingness to seek mental healthcare among males experiencing mental health problems. However, it may reveal growing levels of unmet need, as the burden of psychological distress symptoms continued to increase in females without a comparable increase in mental healthcare, with no indication of decreased willingness to seek mental healthcare in females who have persistently high rates of presentation to primary care for CMD.

The patterns of primary care-recorded CMD showed that the gap in CMD incidence between the most and least deprived areas was narrowing over time. However, when interpreted alongside the data on underlying psychological distress symptoms, this convergence may reflect growing disparities in mental healthcare. We found a higher burden of psychological distress symptoms in more deprived areas than less deprived areas at the start of the study and noted a similar magnitude of increase across all levels of deprivation (approximately 19%), revealing a persistent gradient over time. While the absolute rate of accessing primary mental healthcare was high in most deprived areas, which aligned with the persistently high burden of psychological distress symptoms, these areas saw the smallest increase in mental health services provided in primary care (3.55%) compared with the least deprived areas (16.34%).

The largest increases in both recorded CMD and psychological distress symptoms were observed in those aged 16–19 and those born after 1995. In these groups, we saw that the increases in primary care-recorded CMD exceeded the increase in psychological distress symptoms, including those aged 16–19 (63.30% increase in recorded CMD; 24.22% increase in psychological distress symptoms) and those born between 1995 and 1999 (109.46% increase in recorded CMD; 33.33% increase in psychological distress symptoms). Interestingly, those born between 1990 and 1994 had a similar increase in psychological distress symptoms (27.51%) but a much smaller increase in mental healthcare (29.05%). Further, symptoms of CMD increased by 15.88% in the earliest born cohort (1980–84) but primary care-recorded CMD services decreased by 11.49%.

Taken together, these findings suggest that the groups experiencing the highest burden of psychological distress symptoms may not be the groups most likely to receive care, and while increased symptoms explain some of the increases in primary care provision of mental healthcare, these disparities indicate that the expansion of mental healthcare was not fully explained by the underlying population need for care.

### Strengths and limitations

This study leverages two large UK-representative datasets with a decade of follow-up, offering a broad temporal context for observations of increases in CMD during the COVID-19 pandemic,^9^ which may be extensions of prepandemic trends.

The inclusive case definition of CMD in the CPRD data included symptoms, diagnostic codes and prescriptions which encompass the shared psychopathology of anxiety and depressive disorders increasing the sensitivity of case finding.^10 11^ This definition was particularly relevant to younger populations where diagnostic labels may be applied more conservatively in favour of symptom codes.^12 13^ The GHQ-12 similarly captures psychological distress symptoms, including measures of anxiety, depression and stress. The GHQ-12 has been shown to have high reliability in detecting psychological distress and CMD symptoms in population surveys.^6 14^ However, we did not have access to symptoms like the Patient Health Questionnaire for depression or the Generalised Anxiety Disorder questionnaire for anxiety. The observed changes in psychological distress over time may reflect variations in response to the GHQ-12 over the study period. Increased awareness of mental health in recent decades may lead participants to more readily endorse symptoms of psychological distress, which would impact symptom reporting. Further investigation of the psychometric performance of the GHQ-12 would be valuable in assessing the extent of measurement invariance over time. While the broad case definition allowed us to explore overall trends, it is challenging to precisely interpret the changes over time as they could be due to changing clinical practices or shifting norms around identifying and discussing mental health problems.

The incidence estimates of recorded CMD rely on primary care records. While more than 98% people in the UK are registered in primary care, some groups like asylum seekers, unhoused individuals and institutionalised individuals are excluded. These groups were also excluded from the USoc household sampling frame. The lack of individual-level linkage between these datasets was a limitation of this study, preventing the direct examination of symptom scores among care-seeking individuals. Differences across population groups could impact these findings. For example, later-born cohorts may present to mental healthcare with moderate psychological distress, while other groups may be reluctant to seek care, even when experiencing high psychological distress. Future research that directly compares these data sources would be a valuable addition to the literature, quantifying the patterns of presentation and the extent of unmet need to further disentangle the mechanisms underlying these patterns.

### Meaning of findings

This study adds to evidence of increasing rates of common mental disorders among young adults in the UK, aligning with previous research showing increased primary care rates from the UK^15^ and increased psychological distress symptoms in Europe and North America.^16^,^19^

These patterns may indicate an increased burden of psychological distress symptoms but could also result from increased mental health awareness, reduced mental health stigma and increased help-seeking. Higher self-reported psychological distress may indicate greater recognition of mental health problems and increased willingness to accurately report these symptoms, alongside increased willingness to discuss mental health problems with primary care providers. While changes in primary care practice could contribute to observed patterns, they are unlikely to fully explain these trends as there were few changes to clinical treatment guidelines over the study period.^20 21^ Increased funding for mental health services may have expanded the capacity of primary care to treat mental health problems;^22^ however, increased funding does not clearly explain the differences we observed across sociodemographic group, as it was not targeted to any specific population group.

These findings highlight a rising burden of primary care-recorded CMD and symptoms of psychological distress in those aged 16–19 and later-born cohorts, signalling a pressing public mental health concern. The pronounced increases observed in those aged 16–19 are consistent with research showing that adolescents have more awareness of mental health campaigns, which has been linked to higher mental health literacy, decreased stigma and increased willingness to seek help.^23^ Cohorts born after 1990 show a higher burden of mental health symptoms than previous birth cohorts, which has implications for healthcare planning as many mental disorders which emerge in adolescence persist throughout the life course.^24^ Increased mental healthcare provision to these cohorts may be an important public mental health strategy, as access to timely mental healthcare can improve long-term psychiatric, educational and social outcomes.^25^ While we found large increases in mental healthcare provided to these cohorts, previous research has shown that only 34–56% adolescents with mental disorders have access to mental health services,^26^ and services may not have the capacity to meet the growing demand for care.

While primary care-recorded CMD and psychological distress symptoms increased overall, the differing patterns we observed by ethnicity and deprivation indicate that treatment rates did not change in direct proportion to the changing patterns of psychological distress by sociodemographic group. Despite high levels of psychological distress symptoms in minoritised ethnic groups, the highest levels of mental healthcare utilisation were in those from White ethnic groups, consistent with previous research.^27^ Lower mental healthcare utilisation in minoritised groups compared with White groups, despite similar or higher levels of psychological distress symptoms, may be linked to additional barriers that marginalised individuals may face when seeking care, resulting in more untreated mental health problems in minoritised ethnic groups.^28^ While inequalities persist, we did observe increases in primary care-recorded CMD in minoritised groups over time, which may point to a reduction in the treatment gap for minoritised groups.

The differential patterns of primary care-recorded CMD and self-reported psychological distress symptoms across levels of deprivation present a concerning picture. We found higher burdens of psychological distress symptoms in the most deprived areas, and similar increases in symptom burden over time. However, we found a much larger increase in primary care-recorded CMD in the least deprived areas compared with more deprived areas. These differences in mental healthcare do not align with the underlying symptom burden and instead may reflect the unequal availability of primary healthcare, where there are a higher number of patients per physician in more deprived areas compared with more affluent areas, making it more difficult to access care.^29^ These differences may also indicate inequalities in mental health literacy or differences in help-seeking behaviours across sociodemographic groups. The persistently elevated level of psychological distress symptoms in more deprived areas and comparatively small increases in mental healthcare when compared with more affluent areas reflect the inverse care law, where people most in need of healthcare are least likely to receive it.^30^ These patterns require further investigation, as the level of unmet need may be growing in more deprived areas, despite the greatest need for care, representing an urgent health equity issue.

### Implications

Our findings revealed a substantial rise in both primary care-recorded common mental disorders and self-reported psychological distress symptoms among young adults in the UK from 2009 to 2019, with the increases in psychological distress symptoms outpacing primary care identification and treatment rates. This points to an urgent need to address the growing burden of psychological distress experienced by young adults in the UK.

We found evidence for a growing treatment gap, where marginalised groups experiencing a persistently elevated burden of psychological distress symptoms did not receive the proportionately large increases in mental healthcare in primary care. The mismatch between rising psychological distress symptoms and rates of service provision across sociodemographic groups highlights the urgent need for targeted research which estimates the extent of the treatment gap and the mechanisms underlying these patterns. While further expansion of mental healthcare is warranted to address the growing burden of psychological distress experienced by young adults in the UK, these efforts must consider how the expansion of mental health services aligns with underlying mental health needs to prevent exacerbating existing inequalities.

## Supplementary material

10.1136/bmjment-2024-301457 online supplemental file 1

## Data Availability

Data may be obtained from a third party and are not publicly available.
