# Supplementary material for: Common mental disorders in young adults: temporal trends in primary care episodes and self-reported symptoms
Source: BMJ Ment Health. 2025 May 13;28(1):e301457. doi: 10.1136/bmjment-2024-301457 (PMC12083424; doi:10.1136/bmjment-2024-301457)
Supplement: online supplemental file 1 [file bmjment-28-1-s001.pdf]

# Supplements


Supplement A Additional documentation for CPRD and USoc

Supplement B CPRD code list

Supplement C Supplemental tables for primary care-recorded CMD, CPRD

Supplement D Supplemental tables for self-reported psychological distress symptoms, USoc

## SUPPLEMENT A ADDITIONAL DOCUMENTATION

**Clinical Practice Research Datalink (CPRD) documentation**

### CPRD COHORT SAMPLE & MEASURES

*Study sample:* We used de-identified electronic primary care records from CPRD Aurum and Gold. CPRD contains consultation records including symptoms, diagnoses, and prescriptions, and sociodemographic information, and is broadly representative of the UK population [1,2] More than 98% people in the UK are registered in primary care [1] however some groups were excluded from the cohort, including asylum seekers, unhoused individuals, and people living in institutions [3] We included participants born 1980 and 2003 who were registered with a primary care practice for at least 12 months between 2009 and 2019 (Supplement A figure 1).

Supplement A figure 1 Birth cohorts and ages during follow-up

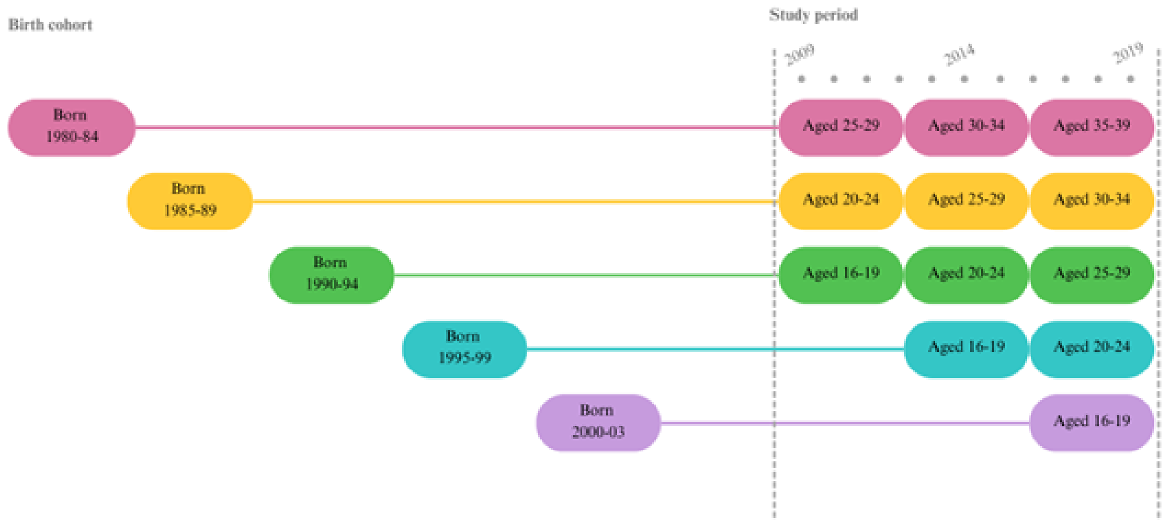


*Deprivation:* Level of deprivation was estimated using the 2019 English Index of Multiple Deprivation (IMD), which is a composite measure of domains of material deprivation: income, employment, education, health, housing, crime, access to services, and living environment calculated for Lower Super Output Areas (LSOA) [4]. The overall score is a weighted sum of the domain indices, and an area-level ranking was produced using postcodes for each practice, mapped onto 2011 LSOA boundaries. Data was provided as fifths to prevent the possibility of deductive disclosure of the location of a practice, from Fifth 1 (least deprived) to Fifth 5 (most deprived) [4] Level of deprivation was estimated for each participant based on the post code of the primary care practice they were registered to. While practices have practice boundaries and participants are likely to register to primary care close to their home address, they may register to a GP outside of this boundary area.

### CPRD SAMPLE CHARACTERISTICS

There were 7,354,888 unique participants in the database, contributing to 26,928,036 person-years of follow-up, which included between 2.1 and 3.2 million participants in each year. Participants were followed for a median of 4.9 person-years (interquartile range: 5.54 person-years; Table 1). There were more females than males in the primary care sample across the study period (female: 52.50% in 2009; 52.73% in 2014; 51.70% in 2019). In 2009, the earliest birth cohort (1980-84) comprised 60.94% of the sample, 31.56% of the sample was born between 1985-89, and 7.50% were born between 1990-94. With the inclusion of later birth cohorts, these proportions were more balanced at the end of the study: 31.00% for 1980-84; 25.83% from 1985-89; 22.34% from 1990-94; 16.98% from 1995-99; and 3.86% from 2000-03.

*Ethnicity:* Of those with ethnicity recorded, most of the sample belonged to the White ethnic group (74.88% in 2009), although this proportion decreased slightly over the study period (70.76% in 2019). These were broadly consistent with population estimates from Census for England & Wales, the Scottish Census, and the Northern Ireland Census (see *Comparison to Census proportions below).*

*Country:* In 2009, 86.39% of the participants were registered to practices in England and 7.45% in Scottish practices, which were consistent with the 2011 census figures, showing that 83.92% of the UK population lived in England and 8.37% lived in Scotland [5] In England, the largest proportion of participants were registered to London-based practices (19.78% in 2009; 24.75% in 2019). The South East region had the next largest proportion of participants: 17.45% in 2009; 16.75% in 2019. The smallest proportion of participants were registered to practices in the North East: 2.50% in 2009 and 2.34% in 2019.

*Deprivation (England only):* In 2009, 50.91% of participants were registered to practices in the two highest fifths of deprivation, while only 12.55% were registered to the least deprived fifth, with similar proportions across all follow-up years (Table 1). Deprivation was estimated for each participant based on the English Index of Multiple Deprivation score estimated for the post code of the primary care practice they were registered to. While practices have practice boundaries and participants are likely to register to primary care close to their home address, they may register to a GP outside of this boundary area. In 2009, 50.91% of participants were registered to practices in the two highest fifths of deprivation, while only 12.55% were registered to the least deprived fifth, with similar proportions across all follow-up years (Table 1).

## MISSING DATA IN CPRD

We had complete information for all variables except ethnicity. The completeness and accuracy of ethnicity recording in CPRD must be considered, as these changed over the study period, which may impact the estimates across ethnic groups [6]. In 2009, 36.70% did not have ethnicity recorded in their patient records, which decreased to 27.14% in 2019. We included those who did not have a recorded ethnicity in the “not stated” category throughout our analysis, however, acknowledge that the "not stated" group is a heterogenous population, making interpretation of these findings challenging.

## COMPARISON TO CENSUS PROPORTIONS

*Ethnicity:* Detailed estimates of ethnic group in the UK population are estimated by Censuses in England & Wales, Scotland, and Northern Ireland separately. These show similar patterns to those observed in the primary care dataset, with a large majority of the population from the White ethnic group, and a small decrease over time in this proportion. The proportions observed in the primary care sample were most similar to those observed in England and Wales, with the proportion of the population from the White ethnic group even higher in Scotland and Northern Ireland than those in England and Wales. The 2011 and 2022 Census for England & Wales estimated that the White ethnic group accounted for 86.0% of the population of England and Wales, decreasing to 81.7% in 2021 [5] The second-largest ethnic group in England and Wales was Asian: 7.5% in 2011 and 9.3% in 2021. Scotland's Census in 2011 estimated that that most of the Scottish population was white: 96.02% in 2011 and 92.87% in 2022 [7,8] The second-largest ethnic group was Asian: 2.66% in 2011 and 3.90% in 2022 [7,8]. The census in Northern Ireland also showed that the majority of the population were in the White ethnic group: 98.7% in 2011 and 96.55% in 2021 [9,10]. The Asian ethnic group was the next largest, comprising 0.80% in 2011 and 1.71% in 2021 [9,10]

*Country*: In 2009, 86.39% of the participants were registered to practices in England and 7.45% in Scottish practices, which were consistent with the 2011 Census figures, showing that 83.92% of the UK population lived in England, 8.37% in Scotland, 4.84 in Northern Ireland, and 2.87% in Wales [5] These were similar to the proportions reported in 2022: 84.48% in England, 8.06% in Scotland, 4.63% in Northern Ireland, and 4.63% in Wales [5].

*Region (England only):* In 2009, 86.39% of the participants were registered to practices in England and 7.45% in Scottish practices, which were consistent with the 2011 census figures, showing that 83.92% of the UK population lived in England and 8.37% lived in Scotland (Appendix A) [5] In England, the largest proportion of participants were registered to London-based practices (19.78% in 2009; 24.75% in 2019). The South East region had the next largest proportion of participants: 17.45% in 2009; 16.75% in 2019. The smallest proportion of participants were registered to practices in the North East: 2.50% in 2009 and 2.34% in 2019.

## Understanding Society (USoc) documentation

### USOC COHORT SAMPLE & MEASURES

*Study sample:* We used data from Understanding Society, a population-representative longitudinal cohort of UK households [11]. We included participants born between 1980 and 2003 who participated in at least one wave between Wave 1 and 10 (Figure 1). Participants were included in 2009, or after their 16th birthday, if later than 2009. While each study wave covers a 24-month period, USoc aims to interview each participant at approximately 12-month intervals.

*Self-reported psychological distress symptoms:* The General Health Questionnaire (GHQ-12) was developed to measure symptoms of psychological distress, including broad symptoms of common mental disorders (CMD) including anxiety, depression [12] The GHQ-12 includes 12 questions which are scored between 0 (much less than usual) and 3 (much more than usual), with positively worded questions reverse coded to result in a score between 0 (no psychological distress symptoms) and 36 (high burden of psychological distress symptoms). The GHQ-12 has been validated for detecting common mental disorders, including psychological distress, depressive symptoms and anxiety symptoms in general population surveys [13,14]
The 12-item General Health Questionnare (GHQ-12) includes 12 questions assessing the severity of mental health symptoms over the past few weeks (0=less than usual, 1 = no more than usual, 2=rather more than usual, 3=much more than usual), with positively worded items reverse-coded so the total score ranges from 0 (no symptoms of psychological distress) to 36 (high burden of symptoms of psychological distress). The following are the items measured on the GHQ-12:

1. Been able to concentrate on what you are doing
2. Lost much sleep over worry
3. Felt you were playing a useful part in things
4. Felt capable of making decisions about things
5. Felt constantly under strain
6. Felt you couldn't overcome your difficulties
7. Been able to enjoy your normal day-to-day activities
8. Been able to face up to your problems
9. Been feeling unhappy and depressed
10. Been lacking confidence in yourself
11. Been thinking of yourself as a worthless person
12. Been feeling reasonably happy, all things considered

Supplement A figure 2 Distribution of psychological distress symptoms (imputed mean GHQ-12 score), Understanding Society Wave 1 (2009-10)

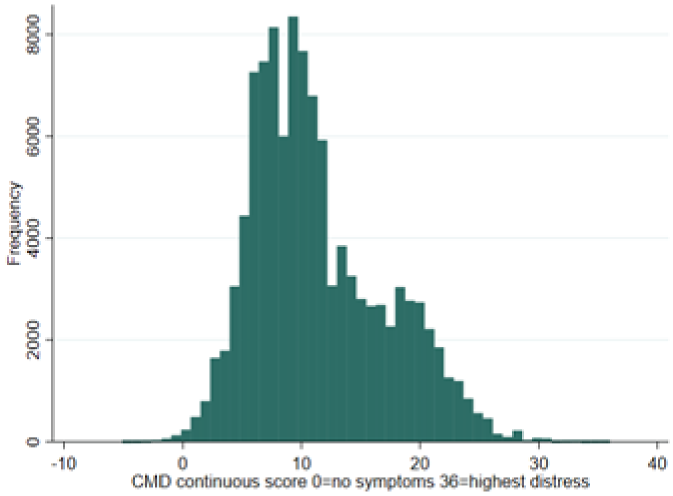

*Ethnicity:* USoc uses multiple sources to derive ethnic group for study participants [11]. Participants were asked about their ethnic and racial group during their first interview and at several other USoc waves. The survey asked participants to select one of the following categories: (1) British/English/Scottish/Welsh/Northern Irish; (2) Irish; (3) Gypsy or Irish Traveller; (4) Any other White background; (5) White and Black Caribbean; (6) White and Black African; (7) White and Asian; (8) any other missed background; (9) Indian; (10) Pakistani; (11) Bangladeshi; (12) Chinese; (13) Any other Asian background; (14) Caribbean; (15) African; (16) Any other Black background; (17) Arab; (18) Any other ethnic group. USoc provides a derived variable which includes information from multiple sources to generate ethnic group, prioritising the latest reported value where there were multiple reports [11]. We grouped these into five broader ethnic groups (1) Asian; (2) Black; (3) Mixed; (4) Other; and (5) White.

*Deprivation:* Level of deprivation was estimated using the Index of Multiple Deprivation (IMD), calculated at the Lower Super Output Area (LSOA). As IMD was calculated differently by devolved nation (country), we restricted this analysis to England only. The IMD scores were divided into fifths from Fifth 1 (least deprived) to Fifth 5 (most deprived). Each household received a IMD score based on the 2011 LSOA boundaries [11].

**USOC SAMPLE CHARACTERISTICS**
We included 24,675 unique participants, with sample sizes in each wave varying from 7,501 (Wave 10) and 10,668 (Wave 6). 30.48% participated in only one wave of data collection, while 6.23% participated in all waves.

The sample characteristics in USoc were similar to CPRD, with a higher proportion of females than males (2009-10: 55.90%), a majority from the White ethnic group (2009-10: 69.15%), and most residing in England (2009-10: 85.57%). The samples differed somewhat by birth cohort, where the proportions were more balanced in USoc. In 2009-10, 38.10% of participants were born between 1980-84, 33.16% were born between 1985-89, and 28.80% were born between 1990-94. In 2019-20, each birth cohort comprised between 18.13% and 23.30% of the total sample.

**MISSING DATA IN USOC**
We explored patterns of missing variables in each wave, comparing the full sample with those who had complete data. Participants with complete data were more likely to be younger, White, and from less deprived areas than those with missing data. We used multiple imputation by chained equations (MICE) to reduce bias from missing data, imputing GHQ score, ethnicity, and deprivation, with the assumption that data were at least missing at random [15]. We estimated the fraction of missing information (FMI) and determined that 50 imputed datasets would be sufficient to ensure replicability of the point estimates. We used all available variables from the analytic models in the imputation models and sample weights were also included in these imputation models. All analysis was run across imputed datasets using Rubin's rules to pool estimates [16]

**WEIGHTING IN USOC**
To account for unequal selection probability and differential non-response, we used the provided cross-sectional weights in each wave to ensure the results were representative of the UK population [17]

**DELTA METHOD FOR CALCULATING THE VARIANCE OF RATIO ESTIMATES**
We used the Delta method to calculate the variance of the ratio of mean estimates (Tables B2-B3), using the following procedure:

Step 1 Calculate the ratio of the point estimates (means):

$$ratio = (\frac{mean_1}{mean_{ref}})$$

Step 2 Calculate the variance ratio using the Delta method:

$$variance\ ratio = (\frac{standard\ error_1}{mean_{ref}})^2 + (\frac{mean_1}{standard\ error_{ref}^{\ 2}})^2$$

Step 3 Calculate the standard error ratio:

$$standard\ error\ ratio = \sqrt{variance\ ratio}$$

Step 4 Calculate the upper and lower bounds of the 95% confidence intervals:

$$lower\ CI = ratio - 1.96\ \times standard\ error\ ratio$$

$$upper\ CI = ratio + 1.96\ \times standard\ error\ ratio$$

**REFERENCES**


1 Herrett E, Gallagher AM, Bhaskaran K, et al. Data resource profile: Clinical Practice Research Datalink (CPRD). Int J Epidemiol. 2015;44:827–36. doi: 10.1093/ije/dyv098

2 Wolf A, Dedman D, Campbell J, et al. Data resource profile: Clinical Practice Research Datalink (CPRD) Aurum. Int J Epidemiol. 2019;48:1740-1740G. doi: 10.1093/ije/dyz034

3 Cocoros NM, Ochoa A, Eberhardt K, et al. Denominators matter: Understanding medical encounter frequency and its impact on surveillance estimates using EHR data. eGEMs. 2019;7:31. doi: 10.5334/egems.292

4 Clinical Practice Research Datalink. Small area level data based on practice postcode: documentation and data dictionary. London 2022.

5 Office for National Statistics. Estimates of the population for the UK, England, Wales, Scotland, and Northern Ireland [dataset]. 2022. https://www.ons.gov.uk/peoplepopulationandcommunity/populationandmigration/populationestimates/datasets/populationestimatesforukenglandandwalesscotlandandnorthernireland (accessed 9 September 2024)

6 Shiekh SI, Harley M, Ghosh RE, et al. Completeness, agreement, and representativeness of ethnicity recording in the United Kingdom's Clinical Practice Research Datalink (CPRD) and linked Hospital Episode Statistics (HES). Popul Health Metr. 2023;21. doi: 10.1186/s12963-023-00302-0

7 National Records of Scotland. Ethnic group [Table UV201]. Scotland's Census 2022. 2024. https://www.scotlandscensus.gov.uk/webapi/jsf/tableView/tableView.xhtml (accessed 12 September 2024)

8 National Records of Scotland. Ethnic group [Table KS201SC]. Scotland's Census 2011. 2012. https://www.scotlandscensus.gov.uk/webapi/jsf/tableView/tableView.xhtml (accessed 12 September 2024)

9 Northern Ireland Statistics and Research Agency. Census 2021 main statistics ethnicity tables. 2022. https://www.nisra.gov.uk/publications/census-2021-main-statistics-ethnicity-tables (accessed 12 September 2024)

10 Northern Ireland Statistics and Research Agency. 2011 Census quick statistics tables on ethnicity, identity, language and religion. 2012. https://www.nisra.gov.uk/publications/2011-census-quick-statistics-tables-ethnicity-identity-language-and-religion (accessed 12 September 2024)
11 Understanding Society. Main survey user guide. Institute for Health and Social Research. 2021;47–8.
12 Goldberg DP. The detection of psychiatric illness by questionnaire: A technique for the identification and assessment of non-psychotic psychiatric illness. Oxford University Press 1972.
13 Lundin A, Hallgren M, Theobald H, et al. Validity of the 12-item version of the General Health Questionnaire in detecting depression in the general population. Public Health. 2016;136:66–74. doi: 10.1016/j.puhe.2016.03.005
14 King K, Allum N, Stoneman P, et al. Estimating measurement equivalence of the 12-item General Health Questionnaire across ethnic groups in the UK. Psychol Med. 2023;53:1778–86. doi: 10.1017/S0033291721003408
15 Azur MJ, Stuart EA, Frangakis C, et al. Multiple imputation by chained equations: what is it and how does it work? Int J Methods Psychiatr Res. 2011;20:40. doi: 10.1002/MPR.329
16 White IR, Royston P, Wood AM. Multiple imputation using chained equations: Issues and guidance for practice. Stat Med. 2011;30:377–99. doi: 10.1002/SIM.4067
17 Benzeval M, Bollinger CR, Burton J, et al. The representativeness of Understanding Society. Colchester 2020.

# SUPPLEMENT B CLINICAL PRACTICE RESEARCH DATALINK (CPRD) CODE LIST

We identified CMD in the Clinical Practice Research Datalink (CPRD) using the code list published by Dykxhoorn et al (2023). This included Read and SNOMED codes related to the (i) symptoms or diagnosis of an anxiety disorder, depressive disorder, mixed depressive and anxiety disorder, or stress, or (ii) pharmaceutical treatment for an anxiety or depressive disorder, including prescriptions for antidepressants and anxiolytics. Code lists were developed by the research team in this previous paper, which included two GPs, a psychiatrist, and psychiatric epidemiologists.

**Reference:**

Dykxhoorn J, Osborn D, Walters K, et al. Temporal patterns in the recorded annual incidence of common mental disorders over two decades in the United Kingdom: a primary care cohort study. Psychol Med. 2023;54:663–74. doi: 10.1017/S0033291723002349

# APPENDIX D: CLINICAL PRACTICE RESEARCH DATALINK (CPRD) CODE LIST

We identified CMD in the Clinical Practice Research Datalink (CPRD) using the code list published by Dykxhoorn et al (2023). This included Read and SNOMED codes related to the (i) symptoms or diagnosis of an anxiety disorder, depressive disorder, mixed depressive and anxiety disorder, or stress, or (ii) pharmaceutical treatment for an anxiety or depressive disorder, including prescriptions for antidepressants and anxiolytics. Code lists were developed by the research team in this previous paper, which included two GPs, a psychiatrist, and psychiatric epidemiologists.

**Reference:**

Dykxhoorn J, Osborn D, Walters K, *et al.* Temporal patterns in the recorded annual incidence of common mental disorders over two decades in the United Kingdom: a primary care cohort study. *Psychol Med*. 2023;54:663–74. doi: 10.1017/S0033291723002349

**Code list:**

| database | code_type | code | flag |
|---|---|---|---|
| aurum | medcode | 1008631000006111 | anxiety |
| aurum | medcode | 1008651000006116 | anxiety |
| aurum | medcode | 1008691000006110 | anxiety |
| aurum | medcode | 1008731000006119 | anxiety |
| aurum | medcode | 1008821000006112 | depression |
| aurum | medcode | 11932111000006112 | anxiety |
| aurum | medcode | 11932121000006116 | anxiety |
| aurum | medcode | 1210253015 | anxiety |
| aurum | medcode | 121671000006117 | stress |
| aurum | medcode | 121771000006110 | stress |
| aurum | medcode | 121781000006113 | stress |
| aurum | medcode | 121881000006115 | stress |
| aurum | medcode | 1222256015 | anxiety |
| aurum | medcode | 1222477019 | depression |
| aurum | medcode | 1222478012 | stress |
| aurum | medcode | 1222480018 | stress |
| aurum | medcode | 1230451012 | anxiety |
| aurum | medcode | 1231321013 | anxiety |
| aurum | medcode | 1231868010 | depression |
| aurum | medcode | 130538010 | anxiety |
| aurum | medcode | 138401000006119 | anxiety |
| aurum | medcode | 138421012 | depression |
| aurum | medcode | 142521000006110 | depression |
| aurum | medcode | 142541000006115 | depression |
| aurum | medcode | 1488626018 | depression |
| aurum | medcode | 1488771018 | depression |
| aurum | medcode | 1494612017 | depression |
| aurum | medcode | 16211000000113 | stress |
| aurum | medcode | 1626511000006116 | anxiety |
| aurum | medcode | 1650771000000113 | depression |
| aurum | medcode | 166341019 | anxiety |
| aurum | medcode | 1680571000006118 | depression |
| aurum | medcode | 1715181000006114 | depression |

| | | | |
|---|---|---|---|
| aurum | medcode | 1715191000006112 | depression |
| aurum | medcode | 1715771000006112 | depression |
| aurum | medcode | 1715781000006110 | depression |
| aurum | medcode | 1721191000006118 | anxiety |
| aurum | medcode | 1721201000006115 | anxiety |
| aurum | medcode | 1721211000006117 | anxiety |
| aurum | medcode | 1721221000006113 | anxiety |
| aurum | medcode | 1752441000006114 | anxiety |
| aurum | medcode | 1752451000006111 | anxiety |
| aurum | medcode | 1752461000006113 | anxiety |
| aurum | medcode | 1755901000006112 | depression |
| aurum | medcode | 1755911000006110 | depression |
| aurum | medcode | 1771531000006110 | depression |
| aurum | medcode | 1773361000006112 | anxiety |
| aurum | medcode | 1773563015 | depression |
| aurum | medcode | 1780506015 | anxiety |
| aurum | medcode | 1785881000006119 | depression |
| aurum | medcode | 1803241000006112 | anxiety |
| aurum | medcode | 1803271000006116 | anxiety |
| aurum | medcode | 1806261000006117 | anxiety |
| aurum | medcode | 1806431000006113 | depression |
| aurum | medcode | 1808511000006119 | anxiety |
| aurum | medcode | 1808521000006110 | anxiety |
| aurum | medcode | 1809331000006113 | anxiety |
| aurum | medcode | 1809341000006115 | anxiety |
| aurum | medcode | 1818111000006112 | anxiety |
| aurum | medcode | 1820771000006113 | depression |
| aurum | medcode | 1820781000006111 | depression |
| aurum | medcode | 1820791000006114 | depression |
| aurum | medcode | 1820801000006110 | depression |
| aurum | medcode | 1821311000006112 | depression |
| aurum | medcode | 1823881000006110 | depression |
| aurum | medcode | 182721000006111 | depression |
| aurum | medcode | 182771000006112 | depression |
| aurum | medcode | 182801000006114 | depression |
| aurum | medcode | 1839551000006116 | depression |
| aurum | medcode | 1839561000006119 | depression |
| aurum | medcode | 1839571000006114 | depression |
| aurum | medcode | 1839581000006112 | depression |
| aurum | medcode | 1839741000006111 | anxiety |
| aurum | medcode | 1839751000006113 | anxiety |
| aurum | medcode | 1839761000006110 | anxiety |
| aurum | medcode | 1839771000006115 | anxiety |
| aurum | medcode | 1839781000006117 | anxiety |
| aurum | medcode | 1839791000006119 | anxiety |
| aurum | medcode | 1839801000006118 | anxiety |
| aurum | medcode | 1861181000006114 | anxiety |
| aurum | medcode | 1882561000006118 | anxiety |
| aurum | medcode | 1956041000006112 | anxiety |

| | | | |
|---|---|---|---|
| aurum | medcode | 1956061000006111 | anxiety |
| aurum | medcode | 1956381000006115 | anxiety |
| aurum | medcode | 1956401000006115 | anxiety |
| aurum | medcode | 1956411000006117 | anxiety |
| aurum | medcode | 1956421000006113 | anxiety |
| aurum | medcode | 1956461000006119 | anxiety |
| aurum | medcode | 1956471000006114 | anxiety |
| aurum | medcode | 1956511000006116 | anxiety |
| aurum | medcode | 1956521000006112 | anxiety |
| aurum | medcode | 1956551000006115 | anxiety |
| aurum | medcode | 1956631000006114 | anxiety |
| aurum | medcode | 1956641000006116 | anxiety |
| aurum | medcode | 1956651000006119 | anxiety |
| aurum | medcode | 1956661000006117 | anxiety |
| aurum | medcode | 1956821000006110 | anxiety |
| aurum | medcode | 1956831000006113 | anxiety |
| aurum | medcode | 1962471000006118 | depression |
| aurum | medcode | 1972071000006118 | depression |
| aurum | medcode | 1972131000006115 | depression |
| aurum | medcode | 1972201000006118 | depression |
| aurum | medcode | 1972311000006112 | depression |
| aurum | medcode | 1972451000006118 | depression |
| aurum | medcode | 1972541000006119 | depression |
| aurum | medcode | 1972661000006119 | depression |
| aurum | medcode | 1972771000006116 | depression |
| aurum | medcode | 1972911000006111 | depression |
| aurum | medcode | 1973381000006112 | depression |
| aurum | medcode | 1973551000006112 | depression |
| aurum | medcode | 1975191000006114 | depression |
| aurum | medcode | 1975211000006110 | depression |
| aurum | medcode | 1975231000006116 | depression |
| aurum | medcode | 1975261000006113 | depression |
| aurum | medcode | 1975281000006115 | depression |
| aurum | medcode | 1975301000006116 | depression |
| aurum | medcode | 1975321000006114 | depression |
| aurum | medcode | 1975981000006114 | depression |
| aurum | medcode | 1975991000006112 | depression |
| aurum | medcode | 1976021000006115 | depression |
| aurum | medcode | 1976051000006112 | depression |
| aurum | medcode | 1976211000006116 | depression |
| aurum | medcode | 1976231000006110 | depression |
| aurum | medcode | 1976251000006115 | depression |
| aurum | medcode | 1976271000006113 | depression |
| aurum | medcode | 1976411000006115 | depression |
| aurum | medcode | 1976491000006113 | mixed |
| aurum | medcode | 1976921000006116 | depression |
| aurum | medcode | 1995011000006112 | depression |
| aurum | medcode | 1995051000006113 | anxiety |
| aurum | medcode | 1995071000006115 | anxiety |

| | | | |
|---|---|---|---|
| aurum | medcode | 1995091000006119 | anxiety |
| aurum | medcode | 202561000006114 | depression |
| aurum | medcode | 213641000000111 | depression |
| aurum | medcode | 2164005017 | depression |
| aurum | medcode | 2164009011 | depression |
| aurum | medcode | 21671000000115 | stress |
| aurum | medcode | 223631000000113 | anxiety |
| aurum | medcode | 223641000000116 | anxiety |
| aurum | medcode | 223651000000118 | depression |
| aurum | medcode | 223741000000112 | depression |
| aurum | medcode | 226411000000110 | depression |
| aurum | medcode | 226581000000119 | depression |
| aurum | medcode | 2370061000000111 | depression |
| aurum | medcode | 2391541000000115 | depression |
| aurum | medcode | 2474715017 | depression |
| aurum | medcode | 251629019 | depression |
| aurum | medcode | 251630012 | anxiety |
| aurum | medcode | 25271000000116 | stress |
| aurum | medcode | 2533375017 | depression |
| aurum | medcode | 2533632018 | anxiety |
| aurum | medcode | 2534091015 | depression |
| aurum | medcode | 2534092010 | depression |
| aurum | medcode | 2534096013 | depression |
| aurum | medcode | 253620013 | anxiety |
| aurum | medcode | 253621012 | anxiety |
| aurum | medcode | 2548573012 | depression |
| aurum | medcode | 2549694013 | stress |
| aurum | medcode | 2549835014 | anxiety |
| aurum | medcode | 2549895012 | anxiety |
| aurum | medcode | 2618241000000117 | anxiety |
| aurum | medcode | 2618361000000113 | anxiety |
| aurum | medcode | 2618401000000116 | anxiety |
| aurum | medcode | 2652351000000117 | anxiety |
| aurum | medcode | 2659511000000118 | anxiety |
| aurum | medcode | 2659531000000114 | anxiety |
| aurum | medcode | 2659601000000116 | depression |
| aurum | medcode | 269821000006113 | anxiety |
| aurum | medcode | 285783010 | stress |
| aurum | medcode | 285790017 | stress |
| aurum | medcode | 294081000000110 | depression |
| aurum | medcode | 294621000000118 | depression |
| aurum | medcode | 294642013 | depression |
| aurum | medcode | 294644014 | depression |
| aurum | medcode | 294646011 | depression |
| aurum | medcode | 294647019 | depression |
| aurum | medcode | 294655014 | depression |
| aurum | medcode | 294824018 | depression |
| aurum | medcode | 294825017 | depression |
| aurum | medcode | 294826016 | depression |

| | | | |
|---|---|---|---|
| aurum | medcode | 294828015 | depression |
| aurum | medcode | 294831019 | depression |
| aurum | medcode | 294832014 | depression |
| aurum | medcode | 294836012 | depression |
| aurum | medcode | 294837015 | depression |
| aurum | medcode | 294838013 | depression |
| aurum | medcode | 294840015 | depression |
| aurum | medcode | 294843018 | depression |
| aurum | medcode | 294844012 | depression |
| aurum | medcode | 294845013 | depression |
| aurum | medcode | 294894013 | depression |
| aurum | medcode | 294917018 | depression |
| aurum | medcode | 294918011 | depression |
| aurum | medcode | 294953016 | anxiety |
| aurum | medcode | 294960010 | anxiety |
| aurum | medcode | 294961014 | anxiety |
| aurum | medcode | 294963012 | anxiety |
| aurum | medcode | 294991016 | anxiety |
| aurum | medcode | 294992011 | anxiety |
| aurum | medcode | 294993018 | anxiety |
| aurum | medcode | 294994012 | anxiety |
| aurum | medcode | 294995013 | anxiety |
| aurum | medcode | 294996014 | anxiety |
| aurum | medcode | 294998010 | anxiety |
| aurum | medcode | 295004011 | anxiety |
| aurum | medcode | 295011010 | anxiety |
| aurum | medcode | 295015018 | anxiety |
| aurum | medcode | 295016017 | anxiety |
| aurum | medcode | 295017014 | anxiety |
| aurum | medcode | 295026012 | anxiety |
| aurum | medcode | 295033012 | anxiety |
| aurum | medcode | 295063015 | anxiety |
| aurum | medcode | 295474019 | anxiety |
| aurum | medcode | 295494011 | depression |
| aurum | medcode | 295535012 | depression |
| aurum | medcode | 295536013 | depression |
| aurum | medcode | 295537016 | depression |
| aurum | medcode | 295572018 | anxiety |
| aurum | medcode | 295584016 | anxiety |
| aurum | medcode | 296137015 | depression |
| aurum | medcode | 296138013 | depression |
| aurum | medcode | 296180012 | depression |
| aurum | medcode | 296181011 | depression |
| aurum | medcode | 296198011 | depression |
| aurum | medcode | 296199015 | depression |
| aurum | medcode | 296224013 | anxiety |
| aurum | medcode | 296236019 | anxiety |
| aurum | medcode | 296237011 | anxiety |
| aurum | medcode | 296238018 | anxiety |

| | | | |
|---|---|---|---|
| aurum | medcode | 296239014 | anxiety |
| aurum | medcode | 296245018 | anxiety |
| aurum | medcode | 296249012 | anxiety |
| aurum | medcode | 296251011 | anxiety |
| aurum | medcode | 296252016 | anxiety |
| aurum | medcode | 296253014 | anxiety |
| aurum | medcode | 296254015 | anxiety |
| aurum | medcode | 296255019 | anxiety |
| aurum | medcode | 296341013 | anxiety |
| aurum | medcode | 296358015 | anxiety |
| aurum | medcode | 296722013 | anxiety |
| aurum | medcode | 300711000000119 | depression |
| aurum | medcode | 302741000006114 | stress |
| aurum | medcode | 304838011 | anxiety |
| aurum | medcode | 30729012 | anxiety |
| aurum | medcode | 318033015 | stress |
| aurum | medcode | 33475016 | anxiety |
| aurum | medcode | 338135018 | stress |
| aurum | medcode | 339044013 | anxiety |
| aurum | medcode | 346972018 | depression |
| aurum | medcode | 346973011 | depression |
| aurum | medcode | 346984016 | anxiety |
| aurum | medcode | 346985015 | anxiety |
| aurum | medcode | 359121000006116 | depression |
| aurum | medcode | 362181000006117 | anxiety |
| aurum | medcode | 362871000006111 | anxiety |
| aurum | medcode | 362881000006114 | anxiety |
| aurum | medcode | 363221000006116 | anxiety |
| aurum | medcode | 363281000006117 | anxiety |
| aurum | medcode | 363361000006117 | anxiety |
| aurum | medcode | 363641000006114 | anxiety |
| aurum | medcode | 363651000006111 | anxiety |
| aurum | medcode | 363661000006113 | anxiety |
| aurum | medcode | 363671000006118 | anxiety |
| aurum | medcode | 363681000006115 | anxiety |
| aurum | medcode | 363691000006117 | anxiety |
| aurum | medcode | 366561000006119 | depression |
| aurum | medcode | 367931000006118 | anxiety |
| aurum | medcode | 369915018 | depression |
| aurum | medcode | 369972011 | depression |
| aurum | medcode | 369974012 | depression |
| aurum | medcode | 369982012 | depression |
| aurum | medcode | 370048011 | anxiety |
| aurum | medcode | 370049015 | anxiety |
| aurum | medcode | 370861000006110 | anxiety |
| aurum | medcode | 371071000006117 | anxiety |
| aurum | medcode | 371341000006110 | anxiety |
| aurum | medcode | 371371000006119 | anxiety |
| aurum | medcode | 376501000006110 | anxiety |

| | | | |
|---|---|---|---|
| aurum | medcode | 376691000006116 | depression |
| aurum | medcode | 376701000006116 | depression |
| aurum | medcode | 376711000006118 | depression |
| aurum | medcode | 376721000006114 | depression |
| aurum | medcode | 376741000006119 | depression |
| aurum | medcode | 376751000006117 | depression |
| aurum | medcode | 376991000006113 | anxiety |
| aurum | medcode | 378291000006117 | anxiety |
| aurum | medcode | 379771000006116 | depression |
| aurum | medcode | 379781000006118 | depression |
| aurum | medcode | 388071000006116 | anxiety |
| aurum | medcode | 389371000006113 | anxiety |
| aurum | medcode | 389391000006114 | anxiety |
| aurum | medcode | 396081000006116 | depression |
| aurum | medcode | 397773012 | stress |
| aurum | medcode | 397774018 | stress |
| aurum | medcode | 397785016 | stress |
| aurum | medcode | 397966011 | stress |
| aurum | medcode | 397967019 | stress |
| aurum | medcode | 398351000006110 | mixed |
| aurum | medcode | 398561000006117 | mixed |
| aurum | medcode | 398841000006119 | depression |
| aurum | medcode | 399961000006118 | depression |
| aurum | medcode | 399981000006111 | anxiety |
| aurum | medcode | 400311000006117 | anxiety |
| aurum | medcode | 400321000006113 | anxiety |
| aurum | medcode | 400351000006116 | anxiety |
| aurum | medcode | 401766011 | depression |
| aurum | medcode | 401780016 | anxiety |
| aurum | medcode | 401783019 | anxiety |
| aurum | medcode | 401785014 | anxiety |
| aurum | medcode | 401866015 | depression |
| aurum | medcode | 401869010 | depression |
| aurum | medcode | 401871010 | depression |
| aurum | medcode | 401872015 | depression |
| aurum | medcode | 401873013 | depression |
| aurum | medcode | 401876017 | depression |
| aurum | medcode | 401881014 | anxiety |
| aurum | medcode | 401888015 | anxiety |
| aurum | medcode | 402503018 | anxiety |
| aurum | medcode | 402505013 | anxiety |
| aurum | medcode | 403931000006116 | anxiety |
| aurum | medcode | 406881000000111 | depression |
| aurum | medcode | 407066012 | depression |
| aurum | medcode | 408521000000112 | depression |
| aurum | medcode | 408541000000117 | depression |
| aurum | medcode | 408561000000116 | depression |
| aurum | medcode | 408581000000113 | depression |
| aurum | medcode | 408601000000116 | depression |

| | | | |
|---|---|---|---|
| aurum | medcode | 410861011 | depression |
| aurum | medcode | 412064013 | stress |
| aurum | medcode | 412065014 | stress |
| aurum | medcode | 412066010 | stress |
| aurum | medcode | 412091010 | stress |
| aurum | medcode | 412299019 | anxiety |
| aurum | medcode | 418031000006113 | anxiety |
| aurum | medcode | 418051000006118 | anxiety |
| aurum | medcode | 418061000006116 | anxiety |
| aurum | medcode | 419841000006116 | anxiety |
| aurum | medcode | 420631000006114 | anxiety |
| aurum | medcode | 420641000006116 | anxiety |
| aurum | medcode | 420671000006112 | anxiety |
| aurum | medcode | 423041000006119 | depression |
| aurum | medcode | 423131000006113 | depression |
| aurum | medcode | 423141000006115 | depression |
| aurum | medcode | 423611000006111 | depression |
| aurum | medcode | 423791000006113 | anxiety |
| aurum | medcode | 424111000006116 | anxiety |
| aurum | medcode | 424201000006112 | anxiety |
| aurum | medcode | 424541000006111 | depression |
| aurum | medcode | 424551000006113 | depression |
| aurum | medcode | 424561000006110 | depression |
| aurum | medcode | 424631000006119 | depression |
| aurum | medcode | 424641000006112 | depression |
| aurum | medcode | 424651000006114 | depression |
| aurum | medcode | 424671000006116 | depression |
| aurum | medcode | 424681000006118 | depression |
| aurum | medcode | 425411000006110 | depression |
| aurum | medcode | 425751000006115 | depression |
| aurum | medcode | 426871000006113 | anxiety |
| aurum | medcode | 426911000006111 | depression |
| aurum | medcode | 426921000006115 | depression |
| aurum | medcode | 426931000006117 | depression |
| aurum | medcode | 426941000006110 | depression |
| aurum | medcode | 426951000006112 | depression |
| aurum | medcode | 426961000006114 | depression |
| aurum | medcode | 426971000006119 | depression |
| aurum | medcode | 426981000006116 | depression |
| aurum | medcode | 426991000006118 | depression |
| aurum | medcode | 427051000006113 | anxiety |
| aurum | medcode | 427061000006110 | anxiety |
| aurum | medcode | 427071000006115 | anxiety |
| aurum | medcode | 427231000006111 | anxiety |
| aurum | medcode | 432511000006119 | depression |
| aurum | medcode | 441512015 | anxiety |
| aurum | medcode | 441826016 | depression |
| aurum | medcode | 442136013 | stress |
| aurum | medcode | 442663013 | anxiety |

| | | | |
|---|---|---|---|
| aurum | medcode | 443717015 | stress |
| aurum | medcode | 460275012 | anxiety |
| aurum | medcode | 460748018 | stress |
| aurum | medcode | 474171000006112 | depression |
| aurum | medcode | 475487017 | anxiety |
| aurum | medcode | 481154010 | anxiety |
| aurum | medcode | 481471000006112 | anxiety |
| aurum | medcode | 481481000006110 | anxiety |
| aurum | medcode | 485870016 | anxiety |
| aurum | medcode | 488201000006114 | anxiety |
| aurum | medcode | 488211000006112 | mixed |
| aurum | medcode | 504351000006118 | anxiety |
| aurum | medcode | 51590017 | depression |
| aurum | medcode | 51671015 | stress |
| aurum | medcode | 525921000006119 | depression |
| aurum | medcode | 54211000006117 | stress |
| aurum | medcode | 550361000006119 | anxiety |
| aurum | medcode | 566981000000113 | anxiety |
| aurum | medcode | 577931000006110 | anxiety |
| aurum | medcode | 613781000006118 | depression |
| aurum | medcode | 613791000006115 | depression |
| aurum | medcode | 626101000006114 | anxiety |
| aurum | medcode | 626121000006116 | anxiety |
| aurum | medcode | 627541000006119 | stress |
| aurum | medcode | 642461000006116 | depression |
| aurum | medcode | 647711000006111 | anxiety |
| aurum | medcode | 647721000006115 | anxiety |
| aurum | medcode | 65032014 | anxiety |
| aurum | medcode | 675861000006113 | depression |
| aurum | medcode | 675881000006115 | anxiety |
| aurum | medcode | 675901000006118 | anxiety |
| aurum | medcode | 675911000006115 | anxiety |
| aurum | medcode | 675921000006111 | anxiety |
| aurum | medcode | 681801000000113 | depression |
| aurum | medcode | 717221000006112 | stress |
| aurum | medcode | 734561000006117 | depression |
| aurum | medcode | 7776981000006112 | anxiety |
| aurum | medcode | 79731000006117 | stress |
| aurum | medcode | 851141000006112 | anxiety |
| aurum | medcode | 851351000006112 | anxiety |
| aurum | medcode | 853201000006116 | anxiety |
| aurum | medcode | 853241000006119 | anxiety |
| aurum | medcode | 853871000006111 | depression |
| aurum | medcode | 882371000006118 | anxiety |
| aurum | medcode | 882381000006115 | anxiety |
| aurum | medcode | 882391000006117 | anxiety |
| aurum | medcode | 882401000006115 | depression |
| aurum | medcode | 882411000006117 | anxiety |
| aurum | medcode | 882421000006113 | depression |

| | | | |
|---|---|---|---|
| aurum | medcode | 882671000006112 | depression |
| aurum | medcode | 882681000006110 | depression |
| aurum | medcode | 882811000006119 | depression |
| aurum | medcode | 882821000006110 | depression |
| aurum | medcode | 882831000006113 | depression |
| aurum | medcode | 90724016 | anxiety |
| aurum | medcode | 908651000006112 | anxiety |
| aurum | medcode | 908731000006114 | depression |
| aurum | medcode | 909681000006110 | depression |
| aurum | medcode | 909691000006113 | anxiety |
| aurum | medcode | 915521000006112 | depression |
| aurum | medcode | 915531000006110 | depression |
| aurum | medcode | 915541000006117 | depression |
| aurum | medcode | 915551000006115 | depression |
| aurum | medcode | 915561000006118 | depression |
| aurum | medcode | 915571000006113 | depression |
| aurum | medcode | 915581000006111 | depression |
| aurum | medcode | 915591000006114 | depression |
| aurum | medcode | 915601000006118 | depression |
| aurum | medcode | 915611000006115 | depression |
| aurum | medcode | 932131000006118 | anxiety |
| aurum | medcode | 959741000006117 | depression |
| aurum | medcode | 959751000006115 | anxiety |
| aurum | medcode | 959761000006118 | depression |
| aurum | medcode | 960061000006117 | anxiety |
| aurum | medcode | 972931000006117 | anxiety |
| aurum | medcode | 97986013 | anxiety |
| aurum | medcode | 980191000006111 | anxiety |
| aurum | medcode | 981121000006116 | anxiety |
| aurum | medcode | 982241000006110 | anxiety |
| aurum | medcode | 982251000006112 | anxiety |
| aurum | medcode | 982261000006114 | anxiety |
| aurum | medcode | 983861000006115 | depression |
| aurum | medcode | 988881000006118 | anxiety |
| aurum | medcode | 991621000006112 | anxiety |
| aurum | medcode | 999901000006113 | depression |
| aurum | medcode | 999911000006111 | depression |
| gold | medcode | 131 | anxiety |
| gold | medcode | 276 | anxiety |
| gold | medcode | 324 | depression |
| gold | medcode | 462 | anxiety |
| gold | medcode | 464 | stress |
| gold | medcode | 465 | stress |
| gold | medcode | 514 | anxiety |
| gold | medcode | 543 | depression |
| gold | medcode | 595 | depression |
| gold | medcode | 636 | anxiety |
| gold | medcode | 655 | mixed |
| gold | medcode | 723 | stress |

| | | | |
|---|---|---|---|
| gold | medcode | 791 | anxiety |
| gold | medcode | 924 | stress |
| gold | medcode | 962 | anxiety |
| gold | medcode | 1055 | depression |
| gold | medcode | 1131 | depression |
| gold | medcode | 1293 | anxiety |
| gold | medcode | 1533 | depression |
| gold | medcode | 1582 | anxiety |
| gold | medcode | 1650 | stress |
| gold | medcode | 1693 | stress |
| gold | medcode | 1758 | anxiety |
| gold | medcode | 1908 | depression |
| gold | medcode | 1996 | depression |
| gold | medcode | 2030 | anxiety |
| gold | medcode | 2291 | stress |
| gold | medcode | 2509 | anxiety |
| gold | medcode | 2524 | anxiety |
| gold | medcode | 2560 | depression |
| gold | medcode | 2571 | anxiety |
| gold | medcode | 2585 | anxiety |
| gold | medcode | 2639 | depression |
| gold | medcode | 2716 | depression |
| gold | medcode | 2777 | depression |
| gold | medcode | 2893 | depression |
| gold | medcode | 2923 | depression |
| gold | medcode | 2930 | depression |
| gold | medcode | 2970 | depression |
| gold | medcode | 2972 | depression |
| gold | medcode | 3076 | anxiety |
| gold | medcode | 3208 | anxiety |
| gold | medcode | 3291 | depression |
| gold | medcode | 3292 | depression |
| gold | medcode | 3328 | anxiety |
| gold | medcode | 3334 | stress |
| gold | medcode | 3407 | anxiety |
| gold | medcode | 3489 | depression |
| gold | medcode | 3502 | depression |
| gold | medcode | 3586 | anxiety |
| gold | medcode | 3667 | depression |
| gold | medcode | 4069 | anxiety |
| gold | medcode | 4081 | anxiety |
| gold | medcode | 4127 | anxiety |
| gold | medcode | 4171 | anxiety |
| gold | medcode | 4323 | depression |
| gold | medcode | 4534 | anxiety |
| gold | medcode | 4634 | anxiety |
| gold | medcode | 4639 | depression |
| gold | medcode | 4659 | anxiety |
| gold | medcode | 4677 | depression |

| | | | |
|---|---|---|---|
| gold | medcode | 4740 | depression |
| gold | medcode | 4824 | depression |
| gold | medcode | 4876 | depression |
| gold | medcode | 4979 | depression |
| gold | medcode | 5304 | anxiety |
| gold | medcode | 5347 | anxiety |
| gold | medcode | 5385 | anxiety |
| gold | medcode | 5678 | anxiety |
| gold | medcode | 5719 | stress |
| gold | medcode | 5726 | depression |
| gold | medcode | 5751 | depression |
| gold | medcode | 5811 | anxiety |
| gold | medcode | 5879 | depression |
| gold | medcode | 5902 | anxiety |
| gold | medcode | 5987 | depression |
| gold | medcode | 6021 | depression |
| gold | medcode | 6121 | depression |
| gold | medcode | 6142 | anxiety |
| gold | medcode | 6212 | depression |
| gold | medcode | 6221 | anxiety |
| gold | medcode | 6408 | anxiety |
| gold | medcode | 6482 | depression |
| gold | medcode | 6546 | depression |
| gold | medcode | 6854 | depression |
| gold | medcode | 6932 | depression |
| gold | medcode | 6939 | anxiety |
| gold | medcode | 6950 | depression |
| gold | medcode | 7011 | depression |
| gold | medcode | 7098 | stress |
| gold | medcode | 7230 | depression |
| gold | medcode | 7235 | depression |
| gold | medcode | 7382 | stress |
| gold | medcode | 7383 | anxiety |
| gold | medcode | 7569 | depression |
| gold | medcode | 7604 | depression |
| gold | medcode | 7737 | depression |
| gold | medcode | 7749 | mixed |
| gold | medcode | 7840 | stress |
| gold | medcode | 7899 | anxiety |
| gold | medcode | 7953 | depression |
| gold | medcode | 7999 | anxiety |
| gold | medcode | 8205 | anxiety |
| gold | medcode | 8250 | stress |
| gold | medcode | 8424 | anxiety |
| gold | medcode | 8478 | depression |
| gold | medcode | 8584 | depression |
| gold | medcode | 8725 | anxiety |
| gold | medcode | 8826 | depression |
| gold | medcode | 8851 | depression |

| | | | |
|---|---|---|---|
| gold | medcode | 8902 | depression |
| gold | medcode | 8928 | depression |
| gold | medcode | 9055 | depression |
| gold | medcode | 9125 | anxiety |
| gold | medcode | 9134 | anxiety |
| gold | medcode | 9183 | depression |
| gold | medcode | 9211 | depression |
| gold | medcode | 9386 | anxiety |
| gold | medcode | 9642 | stress |
| gold | medcode | 9667 | depression |
| gold | medcode | 9796 | depression |
| gold | medcode | 9944 | anxiety |
| gold | medcode | 10015 | depression |
| gold | medcode | 10290 | depression |
| gold | medcode | 10344 | anxiety |
| gold | medcode | 10438 | depression |
| gold | medcode | 10455 | depression |
| gold | medcode | 10535 | anxiety |
| gold | medcode | 10610 | depression |
| gold | medcode | 10667 | depression |
| gold | medcode | 10720 | depression |
| gold | medcode | 10723 | anxiety |
| gold | medcode | 10825 | depression |
| gold | medcode | 11055 | depression |
| gold | medcode | 11098 | anxiety |
| gold | medcode | 11252 | depression |
| gold | medcode | 11329 | depression |
| gold | medcode | 11376 | anxiety |
| gold | medcode | 11607 | anxiety |
| gold | medcode | 11717 | depression |
| gold | medcode | 11764 | anxiety |
| gold | medcode | 11890 | anxiety |
| gold | medcode | 11913 | mixed |
| gold | medcode | 11940 | anxiety |
| gold | medcode | 12080 | stress |
| gold | medcode | 12099 | depression |
| gold | medcode | 12122 | depression |
| gold | medcode | 12303 | depression |
| gold | medcode | 12399 | depression |
| gold | medcode | 12416 | depression |
| gold | medcode | 12450 | depression |
| gold | medcode | 12831 | depression |
| gold | medcode | 12838 | anxiety |
| gold | medcode | 13124 | anxiety |
| gold | medcode | 13307 | depression |
| gold | medcode | 13583 | depression |
| gold | medcode | 13584 | depression |
| gold | medcode | 14709 | depression |
| gold | medcode | 14890 | anxiety |

| | | | |
|---|---|---|---|
| gold | medcode | 15099 | depression |
| gold | medcode | 15155 | depression |
| gold | medcode | 15219 | depression |
| gold | medcode | 15220 | anxiety |
| gold | medcode | 15516 | depression |
| gold | medcode | 15551 | stress |
| gold | medcode | 15566 | anxiety |
| gold | medcode | 15665 | depression |
| gold | medcode | 15923 | depression |
| gold | medcode | 16506 | depression |
| gold | medcode | 16562 | depression |
| gold | medcode | 16632 | depression |
| gold | medcode | 16729 | anxiety |
| gold | medcode | 16861 | depression |
| gold | medcode | 17003 | stress |
| gold | medcode | 17333 | stress |
| gold | medcode | 17420 | anxiety |
| gold | medcode | 17687 | anxiety |
| gold | medcode | 17770 | depression |
| gold | medcode | 17853 | anxiety |
| gold | medcode | 17964 | depression |
| gold | medcode | 18315 | depression |
| gold | medcode | 18399 | anxiety |
| gold | medcode | 18494 | anxiety |
| gold | medcode | 18510 | depression |
| gold | medcode | 18702 | depression |
| gold | medcode | 19000 | anxiety |
| gold | medcode | 19054 | depression |
| gold | medcode | 19409 | depression |
| gold | medcode | 19439 | depression |
| gold | medcode | 19631 | anxiety |
| gold | medcode | 19696 | depression |
| gold | medcode | 19921 | depression |
| gold | medcode | 20089 | anxiety |
| gold | medcode | 20163 | anxiety |
| gold | medcode | 20375 | anxiety |
| gold | medcode | 20634 | anxiety |
| gold | medcode | 20755 | stress |
| gold | medcode | 20773 | anxiety |
| gold | medcode | 20785 | depression |
| gold | medcode | 20881 | anxiety |
| gold | medcode | 21431 | anxiety |
| gold | medcode | 21753 | stress |
| gold | medcode | 21836 | anxiety |
| gold | medcode | 21887 | depression |
| gold | medcode | 22019 | anxiety |
| gold | medcode | 22116 | depression |
| gold | medcode | 22159 | anxiety |
| gold | medcode | 22228 | depression |

| | | | |
|---|---|---|---|
| gold | medcode | 22259 | anxiety |
| gold | medcode | 22264 | depression |
| gold | medcode | 22654 | anxiety |
| gold | medcode | 22687 | depression |
| gold | medcode | 22721 | anxiety |
| gold | medcode | 22806 | depression |
| gold | medcode | 22807 | stress |
| gold | medcode | 22885 | depression |
| gold | medcode | 23713 | depression |
| gold | medcode | 23731 | depression |
| gold | medcode | 23808 | anxiety |
| gold | medcode | 23838 | anxiety |
| gold | medcode | 23869 | stress |
| gold | medcode | 24000 | depression |
| gold | medcode | 24066 | anxiety |
| gold | medcode | 24112 | depression |
| gold | medcode | 24117 | depression |
| gold | medcode | 24171 | depression |
| gold | medcode | 24212 | depression |
| gold | medcode | 24251 | anxiety |
| gold | medcode | 24354 | stress |
| gold | medcode | 24483 | depression |
| gold | medcode | 24530 | stress |
| gold | medcode | 24847 | anxiety |
| gold | medcode | 25213 | anxiety |
| gold | medcode | 25238 | depression |
| gold | medcode | 25344 | anxiety |
| gold | medcode | 25435 | depression |
| gold | medcode | 25563 | depression |
| gold | medcode | 25638 | anxiety |
| gold | medcode | 25697 | depression |
| gold | medcode | 25832 | depression |
| gold | medcode | 26028 | depression |
| gold | medcode | 26079 | anxiety |
| gold | medcode | 26080 | depression |
| gold | medcode | 26138 | anxiety |
| gold | medcode | 26195 | depression |
| gold | medcode | 26208 | anxiety |
| gold | medcode | 26224 | depression |
| gold | medcode | 26251 | stress |
| gold | medcode | 26295 | anxiety |
| gold | medcode | 26331 | depression |
| gold | medcode | 26374 | depression |
| gold | medcode | 26817 | depression |
| gold | medcode | 27491 | depression |
| gold | medcode | 27665 | stress |
| gold | medcode | 27677 | depression |
| gold | medcode | 27685 | anxiety |
| gold | medcode | 27759 | depression |

| | | | |
|---|---|---|---|
| gold | medcode | 27890 | depression |
| gold | medcode | 28008 | depression |
| gold | medcode | 28167 | anxiety |
| gold | medcode | 28248 | depression |
| gold | medcode | 28381 | anxiety |
| gold | medcode | 28408 | anxiety |
| gold | medcode | 28442 | depression |
| gold | medcode | 28677 | depression |
| gold | medcode | 28756 | depression |
| gold | medcode | 28863 | depression |
| gold | medcode | 28925 | anxiety |
| gold | medcode | 28970 | depression |
| gold | medcode | 29342 | depression |
| gold | medcode | 29451 | depression |
| gold | medcode | 29520 | depression |
| gold | medcode | 29527 | depression |
| gold | medcode | 29569 | anxiety |
| gold | medcode | 29579 | depression |
| gold | medcode | 29608 | anxiety |
| gold | medcode | 29707 | stress |
| gold | medcode | 29784 | depression |
| gold | medcode | 29797 | anxiety |
| gold | medcode | 29921 | depression |
| gold | medcode | 30282 | depression |
| gold | medcode | 30405 | depression |
| gold | medcode | 30483 | depression |
| gold | medcode | 30583 | depression |
| gold | medcode | 30688 | depression |
| gold | medcode | 30740 | depression |
| gold | medcode | 31515 | anxiety |
| gold | medcode | 31557 | depression |
| gold | medcode | 31757 | depression |
| gold | medcode | 32159 | depression |
| gold | medcode | 32226 | depression |
| gold | medcode | 32387 | anxiety |
| gold | medcode | 32593 | depression |
| gold | medcode | 32841 | depression |
| gold | medcode | 32845 | depression |
| gold | medcode | 32941 | depression |
| gold | medcode | 33469 | depression |
| gold | medcode | 33618 | depression |
| gold | medcode | 34064 | anxiety |
| gold | medcode | 34275 | depression |
| gold | medcode | 34390 | depression |
| gold | medcode | 34456 | anxiety |
| gold | medcode | 35274 | depression |
| gold | medcode | 35320 | depression |
| gold | medcode | 35607 | depression |
| gold | medcode | 35648 | stress |

| | | | |
|---|---|---|---|
| gold | medcode | 35671 | depression |
| gold | medcode | 35733 | anxiety |
| gold | medcode | 35734 | depression |
| gold | medcode | 35808 | stress |
| gold | medcode | 35825 | anxiety |
| gold | medcode | 36246 | depression |
| gold | medcode | 36616 | depression |
| gold | medcode | 37090 | depression |
| gold | medcode | 37248 | stress |
| gold | medcode | 37296 | depression |
| gold | medcode | 37764 | depression |
| gold | medcode | 37942 | depression |
| gold | medcode | 38058 | depression |
| gold | medcode | 38100 | anxiety |
| gold | medcode | 38194 | depression |
| gold | medcode | 38640 | stress |
| gold | medcode | 38809 | anxiety |
| gold | medcode | 39131 | depression |
| gold | medcode | 39446 | depression |
| gold | medcode | 39447 | depression |
| gold | medcode | 39767 | depression |
| gold | medcode | 39808 | depression |
| gold | medcode | 40578 | stress |
| gold | medcode | 40739 | anxiety |
| gold | medcode | 41022 | depression |
| gold | medcode | 41089 | depression |
| gold | medcode | 41599 | depression |
| gold | medcode | 41989 | depression |
| gold | medcode | 41992 | depression |
| gold | medcode | 42737 | anxiety |
| gold | medcode | 42836 | depression |
| gold | medcode | 42839 | depression |
| gold | medcode | 42857 | depression |
| gold | medcode | 42931 | depression |
| gold | medcode | 42975 | depression |
| gold | medcode | 42988 | depression |
| gold | medcode | 43239 | depression |
| gold | medcode | 43292 | depression |
| gold | medcode | 43296 | anxiety |
| gold | medcode | 43324 | depression |
| gold | medcode | 44287 | depression |
| gold | medcode | 44300 | depression |
| gold | medcode | 44321 | anxiety |
| gold | medcode | 44447 | depression |
| gold | medcode | 44674 | depression |
| gold | medcode | 44848 | depression |
| gold | medcode | 44927 | depression |
| gold | medcode | 45603 | depression |
| gold | medcode | 46045 | depression |

| | | | |
|---|---|---|---|
| gold | medcode | 46244 | depression |
| gold | medcode | 46635 | anxiety |
| gold | medcode | 46796 | depression |
| gold | medcode | 46962 | depression |
| gold | medcode | 47009 | depression |
| gold | medcode | 47365 | anxiety |
| gold | medcode | 47731 | depression |
| gold | medcode | 48349 | depression |
| gold | medcode | 48588 | depression |
| gold | medcode | 48702 | anxiety |
| gold | medcode | 48970 | depression |
| gold | medcode | 50191 | anxiety |
| gold | medcode | 50243 | depression |
| gold | medcode | 50778 | anxiety |
| gold | medcode | 50998 | depression |
| gold | medcode | 51258 | depression |
| gold | medcode | 51349 | anxiety |
| gold | medcode | 52243 | anxiety |
| gold | medcode | 52678 | depression |
| gold | medcode | 53025 | anxiety |
| gold | medcode | 53148 | depression |
| gold | medcode | 53526 | stress |
| gold | medcode | 54999 | depression |
| gold | medcode | 55288 | depression |
| gold | medcode | 55384 | depression |
| gold | medcode | 55631 | depression |
| gold | medcode | 56020 | stress |
| gold | medcode | 56130 | depression |
| gold | medcode | 56273 | depression |
| gold | medcode | 56445 | anxiety |
| gold | medcode | 56609 | depression |
| gold | medcode | 56924 | anxiety |
| gold | medcode | 56982 | depression |
| gold | medcode | 57409 | depression |
| gold | medcode | 57465 | depression |
| gold | medcode | 57673 | depression |
| gold | medcode | 57856 | anxiety |
| gold | medcode | 57949 | depression |
| gold | medcode | 57950 | depression |
| gold | medcode | 58610 | stress |
| gold | medcode | 58774 | depression |
| gold | medcode | 59386 | depression |
| gold | medcode | 59794 | depression |
| gold | medcode | 59869 | depression |
| gold | medcode | 60180 | anxiety |
| gold | medcode | 62935 | anxiety |
| gold | medcode | 62986 | depression |
| gold | medcode | 63249 | stress |
| gold | medcode | 63701 | depression |

| | | | |
|---|---|---|---|
| gold | medcode | 64519 | anxiety |
| gold | medcode | 64584 | depression |
| gold | medcode | 65435 | depression |
| gold | medcode | 65942 | anxiety |
| gold | medcode | 66243 | anxiety |
| gold | medcode | 66333 | depression |
| gold | medcode | 66680 | anxiety |
| gold | medcode | 67918 | anxiety |
| gold | medcode | 68396 | anxiety |
| gold | medcode | 70751 | stress |
| gold | medcode | 71009 | depression |
| gold | medcode | 72026 | depression |
| gold | medcode | 72649 | anxiety |
| gold | medcode | 72966 | depression |
| gold | medcode | 73859 | anxiety |
| gold | medcode | 73991 | depression |
| gold | medcode | 83565 | depression |
| gold | medcode | 85852 | depression |
| gold | medcode | 88644 | depression |
| gold | medcode | 89707 | depression |
| gold | medcode | 91105 | depression |
| gold | medcode | 91242 | stress |
| gold | medcode | 91400 | depression |
| gold | medcode | 91580 | depression |
| gold | medcode | 93401 | anxiety |
| gold | medcode | 94196 | anxiety |
| gold | medcode | 94394 | anxiety |
| gold | medcode | 94436 | depression |
| gold | medcode | 94518 | depression |
| gold | medcode | 94671 | anxiety |
| gold | medcode | 95547 | anxiety |
| gold | medcode | 95954 | anxiety |
| gold | medcode | 96038 | depression |
| gold | medcode | 96504 | anxiety |
| gold | medcode | 96995 | depression |
| gold | medcode | 98252 | depression |
| gold | medcode | 98346 | depression |
| gold | medcode | 98414 | depression |
| gold | medcode | 98417 | depression |
| gold | medcode | 99737 | anxiety |
| gold | medcode | 99798 | depression |
| gold | medcode | 100179 | depression |
| gold | medcode | 100194 | depression |
| gold | medcode | 100977 | depression |
| gold | medcode | 101054 | depression |
| gold | medcode | 101153 | depression |
| gold | medcode | 101250 | depression |
| gold | medcode | 101401 | depression |
| gold | medcode | 101422 | mixed |

| | | | |
|---|---|---|---|
| gold | medcode | 101423 | depression |
| gold | medcode | 101725 | anxiety |
| gold | medcode | 101785 | anxiety |
| gold | medcode | 102106 | anxiety |
| gold | medcode | 102465 | depression |
| gold | medcode | 102632 | depression |
| gold | medcode | 103677 | depression |
| gold | medcode | 103881 | depression |
| gold | medcode | 103903 | depression |
| gold | medcode | 105292 | anxiety |
| gold | medcode | 106143 | depression |
| gold | medcode | 106647 | depression |
| gold | medcode | 106997 | depression |
| gold | medcode | 107134 | anxiety |
| gold | medcode | 107410 | anxiety |
| gold | medcode | 108107 | anxiety |
| gold | medcode | 108194 | anxiety |
| gold | medcode | 108245 | anxiety |
| gold | medcode | 108416 | anxiety |
| gold | medcode | 108741 | depression |
| gold | medcode | 109182 | depression |
| gold | medcode | 109490 | anxiety |
| gold | medcode | 109588 | depression |
| gold | medcode | 109688 | anxiety |
| gold | medcode | 110355 | anxiety |
| gold | medcode | 110943 | depression |
| gold | medcode | 111028 | anxiety |
| gold | medcode | 112522 | anxiety |
| gold | medcode | 112682 | depression |
| gold | medcode | 112968 | anxiety |
| gold | medcode | 113199 | anxiety |
| gold | medcode | 113345 | depression |
| gold | medcode | 113683 | depression |
| gold | medcode | 113793 | depression |
| gold | medcode | 114506 | anxiety |
| aurum | prodcode | 1008941000033114 | antidepressant |
| aurum | prodcode | 1025741000033113 | anxiolytic |
| aurum | prodcode | 1025841000033115 | anxiolytic |
| aurum | prodcode | 10332441000033118 | antidepressant |
| aurum | prodcode | 1042141000033117 | antidepressant |
| aurum | prodcode | 1042241000033112 | antidepressant |
| aurum | prodcode | 10616941000033112 | antidepressant |
| aurum | prodcode | 10617041000033113 | antidepressant |
| aurum | prodcode | 10617141000033112 | antidepressant |
| aurum | prodcode | 10617241000033117 | antidepressant |
| aurum | prodcode | 10617341000033110 | antidepressant |
| aurum | prodcode | 10617441000033116 | antidepressant |
| aurum | prodcode | 10701841000033115 | antidepressant |
| aurum | prodcode | 10701941000033111 | antidepressant |

| | | | |
|---|---|---|---|
| aurum | prodcode | 10730041000033110 | antidepressant |
| aurum | prodcode | 10730141000033114 | antidepressant |
| aurum | prodcode | 1079341000033111 | antidepressant |
| aurum | prodcode | 11072841000033114 | antidepressant |
| aurum | prodcode | 1111541000033117 | antidepressant |
| aurum | prodcode | 1111641000033116 | antidepressant |
| aurum | prodcode | 1111941000033111 | antidepressant |
| aurum | prodcode | 1112441000033114 | antidepressant |
| aurum | prodcode | 11275941000033119 | anxiolytic |
| aurum | prodcode | 1129541000033110 | antidepressant |
| aurum | prodcode | 1135541000033110 | antidepressant |
| aurum | prodcode | 1135941000033116 | antidepressant |
| aurum | prodcode | 11603841000033119 | antidepressant |
| aurum | prodcode | 1165641000033115 | antidepressant |
| aurum | prodcode | 11693641000033117 | antidepressant |
| aurum | prodcode | 11707441000033119 | antidepressant |
| aurum | prodcode | 11707541000033118 | antidepressant |
| aurum | prodcode | 11755441000033119 | antidepressant |
| aurum | prodcode | 12194541000033118 | antidepressant |
| aurum | prodcode | 12194641000033117 | antidepressant |
| aurum | prodcode | 12194741000033114 | antidepressant |
| aurum | prodcode | 12194841000033116 | antidepressant |
| aurum | prodcode | 12257841000033117 | antidepressant |
| aurum | prodcode | 12292341000033116 | antidepressant |
| aurum | prodcode | 12389941000033119 | antidepressant |
| aurum | prodcode | 12390341000033114 | antidepressant |
| aurum | prodcode | 12475941000033119 | antidepressant |
| aurum | prodcode | 12476041000033112 | antidepressant |
| aurum | prodcode | 12476541000033119 | antidepressant |
| aurum | prodcode | 12503341000033119 | anxiolytic |
| aurum | prodcode | 12605341000033111 | antidepressant |
| aurum | prodcode | 12605441000033117 | antidepressant |
| aurum | prodcode | 12605541000033116 | antidepressant |
| aurum | prodcode | 12641541000033113 | antidepressant |
| aurum | prodcode | 12641641000033114 | antidepressant |
| aurum | prodcode | 12679241000033111 | anxiolytic |
| aurum | prodcode | 1278341000033111 | antidepressant |
| aurum | prodcode | 1278441000033117 | antidepressant |
| aurum | prodcode | 1278741000033112 | antidepressant |
| aurum | prodcode | 1278841000033119 | antidepressant |
| aurum | prodcode | 1294541000033115 | antidepressant |
| aurum | prodcode | 1294641000033119 | antidepressant |
| aurum | prodcode | 1294741000033111 | antidepressant |
| aurum | prodcode | 1294841000033118 | antidepressant |
| aurum | prodcode | 13058141000033113 | anxiolytic |
| aurum | prodcode | 13058341000033111 | anxiolytic |
| aurum | prodcode | 13159441000033115 | anxiolytic |
| aurum | prodcode | 13159541000033119 | anxiolytic |
| aurum | prodcode | 13444241000033119 | anxiolytic |

| | | | |
|---|---|---|---|
| aurum | prodcode | 13444341000033112 | anxiolytic |
| aurum | prodcode | 1363541000033112 | anxiolytic |
| aurum | prodcode | 1383241000033113 | anxiolytic |
| aurum | prodcode | 1383341000033115 | anxiolytic |
| aurum | prodcode | 1386141000033114 | anxiolytic |
| aurum | prodcode | 1387141000033111 | anxiolytic |
| aurum | prodcode | 1389441000033112 | antidepressant |
| aurum | prodcode | 1396741000033112 | antidepressant |
| aurum | prodcode | 1396841000033119 | antidepressant |
| aurum | prodcode | 1428141000033113 | anxiolytic |
| aurum | prodcode | 1428241000033118 | anxiolytic |
| aurum | prodcode | 1428441000033117 | anxiolytic |
| aurum | prodcode | 1428541000033116 | anxiolytic |
| aurum | prodcode | 1449441000033113 | antidepressant |
| aurum | prodcode | 1450841000033117 | antidepressant |
| aurum | prodcode | 1454641000033112 | antidepressant |
| aurum | prodcode | 1454741000033115 | antidepressant |
| aurum | prodcode | 1455241000033113 | antidepressant |
| aurum | prodcode | 1455941000033116 | anxiolytic |
| aurum | prodcode | 1461941000033113 | antidepressant |
| aurum | prodcode | 1462241000033110 | antidepressant |
| aurum | prodcode | 1469441000033116 | antidepressant |
| aurum | prodcode | 1471341000033116 | antidepressant |
| aurum | prodcode | 1471441000033110 | antidepressant |
| aurum | prodcode | 1473341000033117 | antidepressant |
| aurum | prodcode | 1473441000033111 | antidepressant |
| aurum | prodcode | 1473641000033113 | antidepressant |
| aurum | prodcode | 1474141000033117 | antidepressant |
| aurum | prodcode | 1481641000033115 | anxiolytic |
| aurum | prodcode | 1481741000033112 | anxiolytic |
| aurum | prodcode | 1502941000033114 | anxiolytic |
| aurum | prodcode | 1509241000033110 | antidepressant |
| aurum | prodcode | 1509341000033117 | antidepressant |
| aurum | prodcode | 1512241000033119 | antidepressant |
| aurum | prodcode | 1512341000033112 | antidepressant |
| aurum | prodcode | 1512441000033118 | antidepressant |
| aurum | prodcode | 1543541000033117 | anxiolytic |
| aurum | prodcode | 1543641000033116 | anxiolytic |
| aurum | prodcode | 1557941000033116 | anxiolytic |
| aurum | prodcode | 1558041000033118 | antidepressant |
| aurum | prodcode | 1558441000033110 | anxiolytic |
| aurum | prodcode | 1562141000033113 | anxiolytic |
| aurum | prodcode | 1562341000033111 | anxiolytic |
| aurum | prodcode | 1562441000033117 | anxiolytic |
| aurum | prodcode | 1563241000033113 | anxiolytic |
| aurum | prodcode | 1583041000033115 | antidepressant |
| aurum | prodcode | 1583141000033116 | antidepressant |
| aurum | prodcode | 1583241000033111 | antidepressant |
| aurum | prodcode | 1583341000033118 | antidepressant |

| | | | |
|---|---|---|---|
| aurum | prodcode | 1603341000033112 | antidepressant |
| aurum | prodcode | 1662441000033114 | antidepressant |
| aurum | prodcode | 1686141000033119 | antidepressant |
| aurum | prodcode | 1699841000033119 | anxiolytic |
| aurum | prodcode | 1712041000033111 | antidepressant |
| aurum | prodcode | 1713541000033112 | anxiolytic |
| aurum | prodcode | 1713641000033113 | anxiolytic |
| aurum | prodcode | 172041000033110 | anxiolytic |
| aurum | prodcode | 172141000033114 | anxiolytic |
| aurum | prodcode | 172241000033119 | anxiolytic |
| aurum | prodcode | 1728141000033116 | anxiolytic |
| aurum | prodcode | 174141000033116 | anxiolytic |
| aurum | prodcode | 1900641000033112 | antidepressant |
| aurum | prodcode | 1900741000033115 | antidepressant |
| aurum | prodcode | 1923141000033118 | antidepressant |
| aurum | prodcode | 2003741000033117 | anxiolytic |
| aurum | prodcode | 2003841000033110 | anxiolytic |
| aurum | prodcode | 2003941000033119 | anxiolytic |
| aurum | prodcode | 2004041000033117 | anxiolytic |
| aurum | prodcode | 2078841000033114 | antidepressant |
| aurum | prodcode | 2215541000033118 | antidepressant |
| aurum | prodcode | 238141000033111 | anxiolytic |
| aurum | prodcode | 238241000033116 | anxiolytic |
| aurum | prodcode | 250641000033119 | anxiolytic |
| aurum | prodcode | 250841000033118 | anxiolytic |
| aurum | prodcode | 259141000033116 | antidepressant |
| aurum | prodcode | 259241000033111 | antidepressant |
| aurum | prodcode | 2602041000033119 | antidepressant |
| aurum | prodcode | 262041000033115 | antidepressant |
| aurum | prodcode | 2621141000033119 | anxiolytic |
| aurum | prodcode | 2621241000033114 | anxiolytic |
| aurum | prodcode | 2621341000033116 | anxiolytic |
| aurum | prodcode | 262141000033116 | antidepressant |
| aurum | prodcode | 262241000033111 | antidepressant |
| aurum | prodcode | 2710441000033119 | antidepressant |
| aurum | prodcode | 2749541000033116 | antidepressant |
| aurum | prodcode | 2749641000033115 | antidepressant |
| aurum | prodcode | 2762341000033113 | anxiolytic |
| aurum | prodcode | 2762441000033119 | anxiolytic |
| aurum | prodcode | 2770541000033113 | antidepressant |
| aurum | prodcode | 2770641000033114 | antidepressant |
| aurum | prodcode | 284741000033110 | antidepressant |
| aurum | prodcode | 285741000033111 | antidepressant |
| aurum | prodcode | 2951341000033116 | antidepressant |
| aurum | prodcode | 2951441000033110 | antidepressant |
| aurum | prodcode | 2974841000033114 | antidepressant |
| aurum | prodcode | 3029641000033112 | antidepressant |
| aurum | prodcode | 3029741000033115 | antidepressant |
| aurum | prodcode | 3029841000033113 | antidepressant |

| | | | |
|---|---|---|---|
| aurum | prodcode | 3029941000033117 | antidepressant |
| aurum | prodcode | 3030341000033110 | antidepressant |
| aurum | prodcode | 3030541000033115 | antidepressant |
| aurum | prodcode | 3033041000033117 | anxiolytic |
| aurum | prodcode | 3033141000033118 | anxiolytic |
| aurum | prodcode | 3058741000033113 | antidepressant |
| aurum | prodcode | 3078541000033115 | antidepressant |
| aurum | prodcode | 3085341000033114 | anxiolytic |
| aurum | prodcode | 3088941000033118 | antidepressant |
| aurum | prodcode | 3089041000033110 | antidepressant |
| aurum | prodcode | 3089141000033114 | antidepressant |
| aurum | prodcode | 3089241000033119 | antidepressant |
| aurum | prodcode | 3177641000033112 | antidepressant |
| aurum | prodcode | 3177741000033115 | antidepressant |
| aurum | prodcode | 3177841000033113 | antidepressant |
| aurum | prodcode | 3177941000033117 | antidepressant |
| aurum | prodcode | 3179941000033111 | antidepressant |
| aurum | prodcode | 3180441000033118 | antidepressant |
| aurum | prodcode | 3180541000033117 | antidepressant |
| aurum | prodcode | 3226641000033111 | antidepressant |
| aurum | prodcode | 3226741000033119 | antidepressant |
| aurum | prodcode | 3226841000033112 | antidepressant |
| aurum | prodcode | 3226941000033116 | antidepressant |
| aurum | prodcode | 3256741000033118 | antidepressant |
| aurum | prodcode | 3256841000033111 | antidepressant |
| aurum | prodcode | 3308041000033119 | anxiolytic |
| aurum | prodcode | 3345241000033111 | anxiolytic |
| aurum | prodcode | 38741000033119 | antidepressant |
| aurum | prodcode | 38841000033112 | antidepressant |
| aurum | prodcode | 3923841000033119 | anxiolytic |
| aurum | prodcode | 3928841000033110 | antidepressant |
| aurum | prodcode | 3928941000033119 | antidepressant |
| aurum | prodcode | 3979041000033112 | anxiolytic |
| aurum | prodcode | 3993641000033114 | anxiolytic |
| aurum | prodcode | 4010441000033114 | antidepressant |
| aurum | prodcode | 4010541000033110 | antidepressant |
| aurum | prodcode | 4079041000033115 | antidepressant |
| aurum | prodcode | 4079141000033116 | antidepressant |
| aurum | prodcode | 41141000033110 | anxiolytic |
| aurum | prodcode | 41241000033115 | anxiolytic |
| aurum | prodcode | 4148341000033116 | antidepressant |
| aurum | prodcode | 4431541000033115 | antidepressant |
| aurum | prodcode | 4434141000033114 | anxiolytic |
| aurum | prodcode | 446441000033119 | anxiolytic |
| aurum | prodcode | 4498541000033116 | antidepressant |
| aurum | prodcode | 4498641000033115 | antidepressant |
| aurum | prodcode | 457941000033112 | anxiolytic |
| aurum | prodcode | 458041000033110 | anxiolytic |
| aurum | prodcode | 458241000033119 | anxiolytic |

| | | | |
|---|---|---|---|
| aurum | prodcode | 458341000033112 | anxiolytic |
| aurum | prodcode | 45841000033119 | antidepressant |
| aurum | prodcode | 458541000033117 | anxiolytic |
| aurum | prodcode | 458641000033116 | anxiolytic |
| aurum | prodcode | 45941000033110 | antidepressant |
| aurum | prodcode | 460041000033115 | anxiolytic |
| aurum | prodcode | 4655141000033113 | antidepressant |
| aurum | prodcode | 4663341000033118 | anxiolytic |
| aurum | prodcode | 466741000033115 | anxiolytic |
| aurum | prodcode | 466841000033113 | anxiolytic |
| aurum | prodcode | 466941000033117 | anxiolytic |
| aurum | prodcode | 472141000033110 | antidepressant |
| aurum | prodcode | 473041000033119 | antidepressant |
| aurum | prodcode | 473141000033115 | antidepressant |
| aurum | prodcode | 473241000033110 | antidepressant |
| aurum | prodcode | 473341000033117 | antidepressant |
| aurum | prodcode | 479641000033111 | antidepressant |
| aurum | prodcode | 4817341000033115 | antidepressant |
| aurum | prodcode | 4817441000033114 | antidepressant |
| aurum | prodcode | 4817641000033111 | antidepressant |
| aurum | prodcode | 4817741000033119 | antidepressant |
| aurum | prodcode | 4818541000033111 | antidepressant |
| aurum | prodcode | 4818641000033112 | antidepressant |
| aurum | prodcode | 4823541000033110 | antidepressant |
| aurum | prodcode | 4823641000033111 | antidepressant |
| aurum | prodcode | 4823741000033119 | antidepressant |
| aurum | prodcode | 4823841000033112 | antidepressant |
| aurum | prodcode | 4824541000033112 | antidepressant |
| aurum | prodcode | 4824641000033113 | antidepressant |
| aurum | prodcode | 4896741000033115 | antidepressant |
| aurum | prodcode | 4896841000033113 | antidepressant |
| aurum | prodcode | 4896941000033117 | antidepressant |
| aurum | prodcode | 4897041000033116 | antidepressant |
| aurum | prodcode | 4897141000033117 | antidepressant |
| aurum | prodcode | 4897241000033112 | antidepressant |
| aurum | prodcode | 489741000033113 | antidepressant |
| aurum | prodcode | 489841000033115 | antidepressant |
| aurum | prodcode | 4899541000033115 | antidepressant |
| aurum | prodcode | 4899641000033119 | antidepressant |
| aurum | prodcode | 4936641000033111 | antidepressant |
| aurum | prodcode | 4936741000033119 | antidepressant |
| aurum | prodcode | 4937041000033115 | antidepressant |
| aurum | prodcode | 4937141000033116 | antidepressant |
| aurum | prodcode | 4954041000033114 | antidepressant |
| aurum | prodcode | 4957241000033112 | anxiolytic |
| aurum | prodcode | 4957841000033111 | antidepressant |
| aurum | prodcode | 4957941000033115 | antidepressant |
| aurum | prodcode | 496741000033118 | antidepressant |
| aurum | prodcode | 498441000033112 | antidepressant |

| | | | |
|---|---|---|---|
| aurum | prodcode | 498541000033113 | antidepressant |
| aurum | prodcode | 499041000033111 | antidepressant |
| aurum | prodcode | 499141000033110 | antidepressant |
| aurum | prodcode | 499241000033115 | antidepressant |
| aurum | prodcode | 5008041000033112 | antidepressant |
| aurum | prodcode | 5052741000033119 | antidepressant |
| aurum | prodcode | 5052841000033112 | antidepressant |
| aurum | prodcode | 5052941000033116 | antidepressant |
| aurum | prodcode | 5053041000033114 | antidepressant |
| aurum | prodcode | 5077041000033116 | antidepressant |
| aurum | prodcode | 5077141000033117 | antidepressant |
| aurum | prodcode | 5128541000033119 | antidepressant |
| aurum | prodcode | 5128641000033118 | antidepressant |
| aurum | prodcode | 5234041000033119 | antidepressant |
| aurum | prodcode | 5234141000033115 | antidepressant |
| aurum | prodcode | 5328741000033117 | antidepressant |
| aurum | prodcode | 5328941000033119 | antidepressant |
| aurum | prodcode | 5329041000033111 | antidepressant |
| aurum | prodcode | 5414941000033113 | anxiolytic |
| aurum | prodcode | 5454141000033118 | antidepressant |
| aurum | prodcode | 5490141000033118 | antidepressant |
| aurum | prodcode | 55441000033119 | antidepressant |
| aurum | prodcode | 55541000033118 | antidepressant |
| aurum | prodcode | 5574641000033117 | antidepressant |
| aurum | prodcode | 5574741000033114 | antidepressant |
| aurum | prodcode | 561941000033115 | antidepressant |
| aurum | prodcode | 563341000033115 | antidepressant |
| aurum | prodcode | 5748641000033110 | anxiolytic |
| aurum | prodcode | 5749441000033115 | antidepressant |
| aurum | prodcode | 577841000033114 | antidepressant |
| aurum | prodcode | 579041000033111 | antidepressant |
| aurum | prodcode | 5812541000033115 | antidepressant |
| aurum | prodcode | 5812641000033119 | antidepressant |
| aurum | prodcode | 5813141000033117 | antidepressant |
| aurum | prodcode | 5813341000033119 | antidepressant |
| aurum | prodcode | 5888641000033118 | antidepressant |
| aurum | prodcode | 5889541000033114 | antidepressant |
| aurum | prodcode | 5889641000033110 | antidepressant |
| aurum | prodcode | 5891041000033116 | antidepressant |
| aurum | prodcode | 5892341000033111 | anxiolytic |
| aurum | prodcode | 5892441000033117 | anxiolytic |
| aurum | prodcode | 5896341000033112 | antidepressant |
| aurum | prodcode | 5896441000033118 | antidepressant |
| aurum | prodcode | 592841000033116 | antidepressant |
| aurum | prodcode | 595541000033113 | antidepressant |
| aurum | prodcode | 5975341000033119 | antidepressant |
| aurum | prodcode | 5978741000033112 | anxiolytic |
| aurum | prodcode | 5991641000033117 | anxiolytic |
| aurum | prodcode | 5991741000033114 | anxiolytic |

| | | | |
|---|---|---|---|
| aurum | prodcode | 5992241000033114 | antidepressant |
| aurum | prodcode | 5996641000033111 | antidepressant |
| aurum | prodcode | 5996741000033119 | antidepressant |
| aurum | prodcode | 6000441000033115 | antidepressant |
| aurum | prodcode | 6029641000033116 | antidepressant |
| aurum | prodcode | 6029741000033113 | antidepressant |
| aurum | prodcode | 60441000033116 | antidepressant |
| aurum | prodcode | 60541000033115 | antidepressant |
| aurum | prodcode | 60641000033119 | antidepressant |
| aurum | prodcode | 60941000033114 | antidepressant |
| aurum | prodcode | 61041000033116 | antidepressant |
| aurum | prodcode | 6161741000033111 | antidepressant |
| aurum | prodcode | 6161841000033118 | antidepressant |
| aurum | prodcode | 61941000033115 | antidepressant |
| aurum | prodcode | 62041000033114 | antidepressant |
| aurum | prodcode | 62141000033113 | antidepressant |
| aurum | prodcode | 626241000033115 | antidepressant |
| aurum | prodcode | 6456641000033117 | anxiolytic |
| aurum | prodcode | 6496341000033116 | antidepressant |
| aurum | prodcode | 664141000033111 | anxiolytic |
| aurum | prodcode | 68641000033117 | antidepressant |
| aurum | prodcode | 70241000033114 | antidepressant |
| aurum | prodcode | 741541000033119 | anxiolytic |
| aurum | prodcode | 745241000033119 | anxiolytic |
| aurum | prodcode | 745341000033112 | anxiolytic |
| aurum | prodcode | 754241000033115 | antidepressant |
| aurum | prodcode | 755541000033119 | antidepressant |
| aurum | prodcode | 787141000033110 | antidepressant |
| aurum | prodcode | 819741000033117 | antidepressant |
| aurum | prodcode | 819841000033110 | antidepressant |
| aurum | prodcode | 8247941000033115 | antidepressant |
| aurum | prodcode | 8248041000033117 | antidepressant |
| aurum | prodcode | 8265541000033112 | antidepressant |
| aurum | prodcode | 8265641000033113 | antidepressant |
| aurum | prodcode | 827841000033112 | anxiolytic |
| aurum | prodcode | 827941000033116 | anxiolytic |
| aurum | prodcode | 8298641000033115 | antidepressant |
| aurum | prodcode | 8298741000033112 | antidepressant |
| aurum | prodcode | 844141000033111 | anxiolytic |
| aurum | prodcode | 849241000033115 | antidepressant |
| aurum | prodcode | 850141000033116 | antidepressant |
| aurum | prodcode | 851141000033111 | anxiolytic |
| aurum | prodcode | 851241000033116 | anxiolytic |
| aurum | prodcode | 851341000033114 | anxiolytic |
| aurum | prodcode | 851441000033115 | anxiolytic |
| aurum | prodcode | 853441000033119 | antidepressant |
| aurum | prodcode | 853541000033118 | antidepressant |
| aurum | prodcode | 853641000033117 | antidepressant |
| aurum | prodcode | 853741000033114 | antidepressant |

| | | | |
|---|---|---|---|
| aurum | prodcode | 854141000033113 | antidepressant |
| aurum | prodcode | 854241000033118 | antidepressant |
| aurum | prodcode | 868841000033115 | antidepressant |
| aurum | prodcode | 868941000033111 | antidepressant |
| aurum | prodcode | 870441000033114 | antidepressant |
| aurum | prodcode | 870741000033119 | antidepressant |
| aurum | prodcode | 870841000033112 | antidepressant |
| aurum | prodcode | 870941000033116 | antidepressant |
| aurum | prodcode | 87641000033116 | antidepressant |
| aurum | prodcode | 87941000033111 | antidepressant |
| aurum | prodcode | 8867941000033118 | antidepressant |
| aurum | prodcode | 8885441000033118 | antidepressant |
| aurum | prodcode | 8885541000033117 | antidepressant |
| aurum | prodcode | 88941000033110 | anxiolytic |
| aurum | prodcode | 901941000033111 | anxiolytic |
| aurum | prodcode | 90841000033115 | anxiolytic |
| aurum | prodcode | 90941000033111 | anxiolytic |
| aurum | prodcode | 9120641000033112 | antidepressant |
| aurum | prodcode | 9120741000033115 | antidepressant |
| aurum | prodcode | 917541000033110 | antidepressant |
| aurum | prodcode | 917641000033111 | antidepressant |
| aurum | prodcode | 917741000033119 | antidepressant |
| aurum | prodcode | 9177941000033119 | anxiolytic |
| aurum | prodcode | 919641000033115 | antidepressant |
| aurum | prodcode | 923041000033117 | antidepressant |
| aurum | prodcode | 923241000033113 | antidepressant |
| aurum | prodcode | 929541000033113 | antidepressant |
| aurum | prodcode | 929841000033110 | antidepressant |
| aurum | prodcode | 9300741000033116 | antidepressant |
| aurum | prodcode | 9300841000033114 | antidepressant |
| aurum | prodcode | 937241000033118 | antidepressant |
| aurum | prodcode | 937841000033119 | antidepressant |
| aurum | prodcode | 938741000033111 | antidepressant |
| aurum | prodcode | 938841000033118 | antidepressant |
| aurum | prodcode | 938941000033114 | antidepressant |
| aurum | prodcode | 939441000033114 | anxiolytic |
| aurum | prodcode | 955241000033110 | antidepressant |
| aurum | prodcode | 966141000033116 | antidepressant |
| aurum | prodcode | 966241000033111 | antidepressant |
| aurum | prodcode | 974341000033116 | anxiolytic |
| aurum | prodcode | 975341000033115 | anxiolytic |
| aurum | prodcode | 989141000033111 | antidepressant |
| aurum | prodcode | 989241000033116 | antidepressant |
| gold | prodcode | 20 | anxiolytic |
| gold | prodcode | 22 | antidepressant |
| gold | prodcode | 35 | anxiolytic |
| gold | prodcode | 49 | antidepressant |
| gold | prodcode | 50 | antidepressant |
| gold | prodcode | 66 | anxiolytic |

| | | | |
|---|---|---|---|
| gold | prodcode | 67 | antidepressant |
| gold | prodcode | 74 | antidepressant |
| gold | prodcode | 83 | antidepressant |
| gold | prodcode | 84 | antidepressant |
| gold | prodcode | 114 | antidepressant |
| gold | prodcode | 182 | antidepressant |
| gold | prodcode | 252 | antidepressant |
| gold | prodcode | 301 | antidepressant |
| gold | prodcode | 418 | antidepressant |
| gold | prodcode | 470 | antidepressant |
| gold | prodcode | 476 | antidepressant |
| gold | prodcode | 487 | antidepressant |
| gold | prodcode | 488 | antidepressant |
| gold | prodcode | 513 | antidepressant |
| gold | prodcode | 527 | antidepressant |
| gold | prodcode | 563 | anxiolytic |
| gold | prodcode | 595 | antidepressant |
| gold | prodcode | 603 | antidepressant |
| gold | prodcode | 623 | antidepressant |
| gold | prodcode | 648 | antidepressant |
| gold | prodcode | 664 | anxiolytic |
| gold | prodcode | 721 | anxiolytic |
| gold | prodcode | 727 | antidepressant |
| gold | prodcode | 742 | antidepressant |
| gold | prodcode | 780 | anxiolytic |
| gold | prodcode | 785 | antidepressant |
| gold | prodcode | 815 | antidepressant |
| gold | prodcode | 841 | antidepressant |
| gold | prodcode | 921 | anxiolytic |
| gold | prodcode | 1088 | anxiolytic |
| gold | prodcode | 1169 | antidepressant |
| gold | prodcode | 1208 | antidepressant |
| gold | prodcode | 1222 | antidepressant |
| gold | prodcode | 1310 | antidepressant |
| gold | prodcode | 1397 | antidepressant |
| gold | prodcode | 1453 | antidepressant |
| gold | prodcode | 1474 | antidepressant |
| gold | prodcode | 1575 | antidepressant |
| gold | prodcode | 1612 | antidepressant |
| gold | prodcode | 1712 | antidepressant |
| gold | prodcode | 1729 | anxiolytic |
| gold | prodcode | 1730 | antidepressant |
| gold | prodcode | 1809 | antidepressant |
| gold | prodcode | 1888 | antidepressant |
| gold | prodcode | 1940 | antidepressant |
| gold | prodcode | 2017 | anxiolytic |
| gold | prodcode | 2039 | antidepressant |
| gold | prodcode | 2091 | anxiolytic |
| gold | prodcode | 2093 | antidepressant |

| | | | |
|---|---|---|---|
| gold | prodcode | 2290 | antidepressant |
| gold | prodcode | 2320 | antidepressant |
| gold | prodcode | 2356 | antidepressant |
| gold | prodcode | 2394 | anxiolytic |
| gold | prodcode | 2403 | anxiolytic |
| gold | prodcode | 2407 | anxiolytic |
| gold | prodcode | 2408 | antidepressant |
| gold | prodcode | 2486 | antidepressant |
| gold | prodcode | 2525 | antidepressant |
| gold | prodcode | 2531 | antidepressant |
| gold | prodcode | 2532 | antidepressant |
| gold | prodcode | 2548 | antidepressant |
| gold | prodcode | 2579 | antidepressant |
| gold | prodcode | 2617 | antidepressant |
| gold | prodcode | 2654 | antidepressant |
| gold | prodcode | 2828 | anxiolytic |
| gold | prodcode | 2880 | antidepressant |
| gold | prodcode | 2883 | antidepressant |
| gold | prodcode | 2897 | antidepressant |
| gold | prodcode | 2936 | antidepressant |
| gold | prodcode | 2985 | antidepressant |
| gold | prodcode | 3083 | antidepressant |
| gold | prodcode | 3126 | anxiolytic |
| gold | prodcode | 3183 | antidepressant |
| gold | prodcode | 3194 | antidepressant |
| gold | prodcode | 3196 | antidepressant |
| gold | prodcode | 3205 | anxiolytic |
| gold | prodcode | 3320 | anxiolytic |
| gold | prodcode | 3349 | antidepressant |
| gold | prodcode | 3351 | antidepressant |
| gold | prodcode | 3354 | anxiolytic |
| gold | prodcode | 3355 | antidepressant |
| gold | prodcode | 3356 | antidepressant |
| gold | prodcode | 3357 | anxiolytic |
| gold | prodcode | 3391 | antidepressant |
| gold | prodcode | 3490 | antidepressant |
| gold | prodcode | 3491 | anxiolytic |
| gold | prodcode | 3524 | anxiolytic |
| gold | prodcode | 3554 | antidepressant |
| gold | prodcode | 3574 | anxiolytic |
| gold | prodcode | 3601 | antidepressant |
| gold | prodcode | 3652 | antidepressant |
| gold | prodcode | 3657 | antidepressant |
| gold | prodcode | 3670 | antidepressant |
| gold | prodcode | 3686 | anxiolytic |
| gold | prodcode | 3687 | anxiolytic |
| gold | prodcode | 3741 | anxiolytic |
| gold | prodcode | 3777 | antidepressant |
| gold | prodcode | 3783 | antidepressant |

| | | | |
|---|---|---|---|
| gold | prodcode | 3842 | antidepressant |
| gold | prodcode | 3861 | antidepressant |
| gold | prodcode | 3870 | anxiolytic |
| gold | prodcode | 3903 | antidepressant |
| gold | prodcode | 3925 | antidepressant |
| gold | prodcode | 3955 | antidepressant |
| gold | prodcode | 4003 | antidepressant |
| gold | prodcode | 4011 | antidepressant |
| gold | prodcode | 4020 | antidepressant |
| gold | prodcode | 4075 | antidepressant |
| gold | prodcode | 4118 | antidepressant |
| gold | prodcode | 4140 | anxiolytic |
| gold | prodcode | 4187 | anxiolytic |
| gold | prodcode | 4194 | antidepressant |
| gold | prodcode | 4218 | antidepressant |
| gold | prodcode | 4297 | antidepressant |
| gold | prodcode | 4310 | antidepressant |
| gold | prodcode | 4321 | antidepressant |
| gold | prodcode | 4329 | antidepressant |
| gold | prodcode | 4338 | anxiolytic |
| gold | prodcode | 4352 | antidepressant |
| gold | prodcode | 4404 | antidepressant |
| gold | prodcode | 4411 | antidepressant |
| gold | prodcode | 4422 | antidepressant |
| gold | prodcode | 4554 | antidepressant |
| gold | prodcode | 4682 | antidepressant |
| gold | prodcode | 4690 | antidepressant |
| gold | prodcode | 4726 | antidepressant |
| gold | prodcode | 4770 | antidepressant |
| gold | prodcode | 4874 | antidepressant |
| gold | prodcode | 4907 | antidepressant |
| gold | prodcode | 5058 | anxiolytic |
| gold | prodcode | 5073 | antidepressant |
| gold | prodcode | 5187 | antidepressant |
| gold | prodcode | 5190 | antidepressant |
| gold | prodcode | 5306 | anxiolytic |
| gold | prodcode | 5352 | anxiolytic |
| gold | prodcode | 5385 | anxiolytic |
| gold | prodcode | 5459 | anxiolytic |
| gold | prodcode | 5611 | antidepressant |
| gold | prodcode | 5710 | antidepressant |
| gold | prodcode | 5832 | antidepressant |
| gold | prodcode | 5916 | anxiolytic |
| gold | prodcode | 6054 | antidepressant |
| gold | prodcode | 6218 | antidepressant |
| gold | prodcode | 6255 | antidepressant |
| gold | prodcode | 6274 | antidepressant |
| gold | prodcode | 6312 | antidepressant |
| gold | prodcode | 6360 | antidepressant |

| | | | |
|---|---|---|---|
| gold | prodcode | 6405 | antidepressant |
| gold | prodcode | 6421 | antidepressant |
| gold | prodcode | 6442 | antidepressant |
| gold | prodcode | 6481 | antidepressant |
| gold | prodcode | 6488 | antidepressant |
| gold | prodcode | 6795 | antidepressant |
| gold | prodcode | 6846 | antidepressant |
| gold | prodcode | 6854 | antidepressant |
| gold | prodcode | 6894 | antidepressant |
| gold | prodcode | 6895 | antidepressant |
| gold | prodcode | 7059 | antidepressant |
| gold | prodcode | 7122 | antidepressant |
| gold | prodcode | 7147 | antidepressant |
| gold | prodcode | 7153 | antidepressant |
| gold | prodcode | 7328 | antidepressant |
| gold | prodcode | 7444 | anxiolytic |
| gold | prodcode | 7468 | antidepressant |
| gold | prodcode | 7515 | antidepressant |
| gold | prodcode | 7567 | anxiolytic |
| gold | prodcode | 7569 | anxiolytic |
| gold | prodcode | 7677 | antidepressant |
| gold | prodcode | 7678 | antidepressant |
| gold | prodcode | 7693 | antidepressant |
| gold | prodcode | 7751 | antidepressant |
| gold | prodcode | 7780 | antidepressant |
| gold | prodcode | 7786 | anxiolytic |
| gold | prodcode | 7894 | antidepressant |
| gold | prodcode | 7910 | antidepressant |
| gold | prodcode | 7924 | anxiolytic |
| gold | prodcode | 8055 | antidepressant |
| gold | prodcode | 8144 | antidepressant |
| gold | prodcode | 8174 | antidepressant |
| gold | prodcode | 8332 | antidepressant |
| gold | prodcode | 8493 | antidepressant |
| gold | prodcode | 8549 | antidepressant |
| gold | prodcode | 8585 | antidepressant |
| gold | prodcode | 8640 | antidepressant |
| gold | prodcode | 8661 | antidepressant |
| gold | prodcode | 8719 | antidepressant |
| gold | prodcode | 8720 | antidepressant |
| gold | prodcode | 8721 | anxiolytic |
| gold | prodcode | 8726 | antidepressant |
| gold | prodcode | 8798 | anxiolytic |
| gold | prodcode | 8831 | antidepressant |
| gold | prodcode | 8878 | antidepressant |
| gold | prodcode | 8913 | anxiolytic |
| gold | prodcode | 8928 | antidepressant |
| gold | prodcode | 9008 | anxiolytic |
| gold | prodcode | 9048 | anxiolytic |

| | | | |
|---|---|---|---|
| gold | prodcode | 9111 | anxiolytic |
| gold | prodcode | 9182 | antidepressant |
| gold | prodcode | 9206 | antidepressant |
| gold | prodcode | 9534 | antidepressant |
| gold | prodcode | 9558 | antidepressant |
| gold | prodcode | 9598 | anxiolytic |
| gold | prodcode | 9696 | anxiolytic |
| gold | prodcode | 9814 | anxiolytic |
| gold | prodcode | 10083 | antidepressant |
| gold | prodcode | 10402 | anxiolytic |
| gold | prodcode | 10413 | antidepressant |
| gold | prodcode | 10430 | anxiolytic |
| gold | prodcode | 10645 | antidepressant |
| gold | prodcode | 10787 | antidepressant |
| gold | prodcode | 10802 | anxiolytic |
| gold | prodcode | 10948 | antidepressant |
| gold | prodcode | 10954 | anxiolytic |
| gold | prodcode | 11486 | anxiolytic |
| gold | prodcode | 11956 | antidepressant |
| gold | prodcode | 11963 | antidepressant |
| gold | prodcode | 12100 | antidepressant |
| gold | prodcode | 12123 | antidepressant |
| gold | prodcode | 12125 | antidepressant |
| gold | prodcode | 12129 | antidepressant |
| gold | prodcode | 12192 | antidepressant |
| gold | prodcode | 12207 | antidepressant |
| gold | prodcode | 12221 | antidepressant |
| gold | prodcode | 12222 | antidepressant |
| gold | prodcode | 12293 | anxiolytic |
| gold | prodcode | 12353 | antidepressant |
| gold | prodcode | 12368 | antidepressant |
| gold | prodcode | 12462 | anxiolytic |
| gold | prodcode | 12477 | anxiolytic |
| gold | prodcode | 12503 | antidepressant |
| gold | prodcode | 12549 | antidepressant |
| gold | prodcode | 12598 | anxiolytic |
| gold | prodcode | 12710 | antidepressant |
| gold | prodcode | 13151 | antidepressant |
| gold | prodcode | 13237 | antidepressant |
| gold | prodcode | 13279 | anxiolytic |
| gold | prodcode | 13558 | antidepressant |
| gold | prodcode | 13621 | antidepressant |
| gold | prodcode | 14365 | anxiolytic |
| gold | prodcode | 14398 | antidepressant |
| gold | prodcode | 14410 | antidepressant |
| gold | prodcode | 14519 | antidepressant |
| gold | prodcode | 14534 | antidepressant |
| gold | prodcode | 14578 | antidepressant |
| gold | prodcode | 14740 | antidepressant |

| | | | |
|---|---|---|---|
| gold | prodcode | 14803 | antidepressant |
| gold | prodcode | 14849 | antidepressant |
| gold | prodcode | 15110 | anxiolytic |
| gold | prodcode | 15163 | antidepressant |
| gold | prodcode | 15268 | antidepressant |
| gold | prodcode | 15380 | antidepressant |
| gold | prodcode | 15492 | anxiolytic |
| gold | prodcode | 15632 | antidepressant |
| gold | prodcode | 15852 | anxiolytic |
| gold | prodcode | 15975 | antidepressant |
| gold | prodcode | 16154 | antidepressant |
| gold | prodcode | 16169 | anxiolytic |
| gold | prodcode | 16323 | antidepressant |
| gold | prodcode | 16727 | antidepressant |
| gold | prodcode | 16969 | antidepressant |
| gold | prodcode | 17183 | antidepressant |
| gold | prodcode | 17294 | anxiolytic |
| gold | prodcode | 17319 | antidepressant |
| gold | prodcode | 17830 | anxiolytic |
| gold | prodcode | 18291 | anxiolytic |
| gold | prodcode | 18342 | antidepressant |
| gold | prodcode | 19168 | antidepressant |
| gold | prodcode | 19181 | antidepressant |
| gold | prodcode | 19183 | antidepressant |
| gold | prodcode | 19186 | antidepressant |
| gold | prodcode | 19299 | anxiolytic |
| gold | prodcode | 19450 | anxiolytic |
| gold | prodcode | 19470 | antidepressant |
| gold | prodcode | 19779 | antidepressant |
| gold | prodcode | 20026 | antidepressant |
| gold | prodcode | 20152 | antidepressant |
| gold | prodcode | 20164 | anxiolytic |
| gold | prodcode | 20245 | anxiolytic |
| gold | prodcode | 20405 | antidepressant |
| gold | prodcode | 20504 | antidepressant |
| gold | prodcode | 20571 | antidepressant |
| gold | prodcode | 20801 | anxiolytic |
| gold | prodcode | 21081 | antidepressant |
| gold | prodcode | 21157 | antidepressant |
| gold | prodcode | 21357 | antidepressant |
| gold | prodcode | 21437 | anxiolytic |
| gold | prodcode | 21454 | anxiolytic |
| gold | prodcode | 21819 | antidepressant |
| gold | prodcode | 21820 | antidepressant |
| gold | prodcode | 22070 | antidepressant |
| gold | prodcode | 23120 | anxiolytic |
| gold | prodcode | 23426 | antidepressant |
| gold | prodcode | 23874 | anxiolytic |
| gold | prodcode | 24134 | antidepressant |

| | | | |
|---|---|---|---|
| gold | prodcode | 24135 | anxiolytic |
| gold | prodcode | 24141 | antidepressant |
| gold | prodcode | 24145 | antidepressant |
| gold | prodcode | 24147 | antidepressant |
| gold | prodcode | 24152 | antidepressant |
| gold | prodcode | 24680 | antidepressant |
| gold | prodcode | 24723 | antidepressant |
| gold | prodcode | 24890 | antidepressant |
| gold | prodcode | 25273 | anxiolytic |
| gold | prodcode | 25444 | antidepressant |
| gold | prodcode | 26016 | antidepressant |
| gold | prodcode | 26056 | antidepressant |
| gold | prodcode | 26213 | antidepressant |
| gold | prodcode | 26513 | antidepressant |
| gold | prodcode | 27008 | antidepressant |
| gold | prodcode | 27367 | anxiolytic |
| gold | prodcode | 27847 | anxiolytic |
| gold | prodcode | 28703 | anxiolytic |
| gold | prodcode | 28880 | anxiolytic |
| gold | prodcode | 29219 | anxiolytic |
| gold | prodcode | 29339 | antidepressant |
| gold | prodcode | 29441 | anxiolytic |
| gold | prodcode | 29756 | antidepressant |
| gold | prodcode | 29786 | antidepressant |
| gold | prodcode | 29857 | antidepressant |
| gold | prodcode | 29869 | anxiolytic |
| gold | prodcode | 29875 | antidepressant |
| gold | prodcode | 30056 | anxiolytic |
| gold | prodcode | 30258 | antidepressant |
| gold | prodcode | 30321 | anxiolytic |
| gold | prodcode | 30375 | antidepressant |
| gold | prodcode | 30376 | antidepressant |
| gold | prodcode | 30377 | anxiolytic |
| gold | prodcode | 30779 | anxiolytic |
| gold | prodcode | 30981 | anxiolytic |
| gold | prodcode | 30983 | antidepressant |
| gold | prodcode | 30985 | anxiolytic |
| gold | prodcode | 31710 | anxiolytic |
| gold | prodcode | 31824 | antidepressant |
| gold | prodcode | 31826 | antidepressant |
| gold | prodcode | 32121 | antidepressant |
| gold | prodcode | 32231 | anxiolytic |
| gold | prodcode | 32320 | anxiolytic |
| gold | prodcode | 32401 | antidepressant |
| gold | prodcode | 32439 | antidepressant |
| gold | prodcode | 32546 | antidepressant |
| gold | prodcode | 32847 | anxiolytic |
| gold | prodcode | 32848 | antidepressant |
| gold | prodcode | 32863 | antidepressant |

| | | | |
|---|---|---|---|
| gold | prodcode | 32899 | antidepressant |
| gold | prodcode | 33045 | anxiolytic |
| gold | prodcode | 33070 | anxiolytic |
| gold | prodcode | 33071 | antidepressant |
| gold | prodcode | 33074 | antidepressant |
| gold | prodcode | 33086 | anxiolytic |
| gold | prodcode | 33090 | antidepressant |
| gold | prodcode | 33164 | antidepressant |
| gold | prodcode | 33337 | antidepressant |
| gold | prodcode | 33410 | antidepressant |
| gold | prodcode | 33624 | antidepressant |
| gold | prodcode | 33648 | anxiolytic |
| gold | prodcode | 33663 | anxiolytic |
| gold | prodcode | 33720 | antidepressant |
| gold | prodcode | 33779 | antidepressant |
| gold | prodcode | 33841 | anxiolytic |
| gold | prodcode | 33978 | antidepressant |
| gold | prodcode | 34002 | anxiolytic |
| gold | prodcode | 34003 | antidepressant |
| gold | prodcode | 34046 | antidepressant |
| gold | prodcode | 34058 | antidepressant |
| gold | prodcode | 34107 | antidepressant |
| gold | prodcode | 34129 | antidepressant |
| gold | prodcode | 34182 | antidepressant |
| gold | prodcode | 34197 | antidepressant |
| gold | prodcode | 34202 | antidepressant |
| gold | prodcode | 34216 | antidepressant |
| gold | prodcode | 34222 | antidepressant |
| gold | prodcode | 34223 | antidepressant |
| gold | prodcode | 34224 | antidepressant |
| gold | prodcode | 34245 | antidepressant |
| gold | prodcode | 34251 | antidepressant |
| gold | prodcode | 34274 | antidepressant |
| gold | prodcode | 34288 | antidepressant |
| gold | prodcode | 34292 | anxiolytic |
| gold | prodcode | 34294 | antidepressant |
| gold | prodcode | 34331 | anxiolytic |
| gold | prodcode | 34351 | antidepressant |
| gold | prodcode | 34355 | antidepressant |
| gold | prodcode | 34356 | antidepressant |
| gold | prodcode | 34361 | anxiolytic |
| gold | prodcode | 34372 | anxiolytic |
| gold | prodcode | 34401 | antidepressant |
| gold | prodcode | 34406 | anxiolytic |
| gold | prodcode | 34408 | anxiolytic |
| gold | prodcode | 34413 | antidepressant |
| gold | prodcode | 34415 | antidepressant |
| gold | prodcode | 34419 | antidepressant |
| gold | prodcode | 34421 | antidepressant |

| | | | |
|---|---|---|---|
| gold | prodcode | 34436 | antidepressant |
| gold | prodcode | 34456 | antidepressant |
| gold | prodcode | 34466 | antidepressant |
| gold | prodcode | 34470 | antidepressant |
| gold | prodcode | 34474 | antidepressant |
| gold | prodcode | 34498 | antidepressant |
| gold | prodcode | 34499 | antidepressant |
| gold | prodcode | 34503 | antidepressant |
| gold | prodcode | 34508 | anxiolytic |
| gold | prodcode | 34516 | anxiolytic |
| gold | prodcode | 34525 | antidepressant |
| gold | prodcode | 34534 | anxiolytic |
| gold | prodcode | 34555 | anxiolytic |
| gold | prodcode | 34561 | anxiolytic |
| gold | prodcode | 34572 | anxiolytic |
| gold | prodcode | 34578 | antidepressant |
| gold | prodcode | 34580 | antidepressant |
| gold | prodcode | 34586 | antidepressant |
| gold | prodcode | 34587 | antidepressant |
| gold | prodcode | 34603 | antidepressant |
| gold | prodcode | 34612 | anxiolytic |
| gold | prodcode | 34634 | antidepressant |
| gold | prodcode | 34641 | antidepressant |
| gold | prodcode | 34642 | anxiolytic |
| gold | prodcode | 34643 | antidepressant |
| gold | prodcode | 34672 | antidepressant |
| gold | prodcode | 34681 | anxiolytic |
| gold | prodcode | 34686 | anxiolytic |
| gold | prodcode | 34692 | anxiolytic |
| gold | prodcode | 34722 | antidepressant |
| gold | prodcode | 34731 | antidepressant |
| gold | prodcode | 34745 | antidepressant |
| gold | prodcode | 34770 | anxiolytic |
| gold | prodcode | 34777 | anxiolytic |
| gold | prodcode | 34782 | antidepressant |
| gold | prodcode | 34806 | anxiolytic |
| gold | prodcode | 34813 | antidepressant |
| gold | prodcode | 34822 | antidepressant |
| gold | prodcode | 34823 | anxiolytic |
| gold | prodcode | 34849 | antidepressant |
| gold | prodcode | 34856 | antidepressant |
| gold | prodcode | 34866 | antidepressant |
| gold | prodcode | 34871 | antidepressant |
| gold | prodcode | 34872 | antidepressant |
| gold | prodcode | 34874 | anxiolytic |
| gold | prodcode | 34876 | anxiolytic |
| gold | prodcode | 34892 | anxiolytic |
| gold | prodcode | 34897 | anxiolytic |
| gold | prodcode | 34916 | antidepressant |

| | | | |
|---|---|---|---|
| gold | prodcode | 34950 | antidepressant |
| gold | prodcode | 34964 | anxiolytic |
| gold | prodcode | 34966 | antidepressant |
| gold | prodcode | 34970 | antidepressant |
| gold | prodcode | 35021 | antidepressant |
| gold | prodcode | 35112 | antidepressant |
| gold | prodcode | 35258 | antidepressant |
| gold | prodcode | 35493 | antidepressant |
| gold | prodcode | 35932 | anxiolytic |
| gold | prodcode | 36200 | anxiolytic |
| gold | prodcode | 36581 | anxiolytic |
| gold | prodcode | 36602 | anxiolytic |
| gold | prodcode | 36611 | anxiolytic |
| gold | prodcode | 36746 | antidepressant |
| gold | prodcode | 36893 | antidepressant |
| gold | prodcode | 37256 | antidepressant |
| gold | prodcode | 37325 | anxiolytic |
| gold | prodcode | 37566 | anxiolytic |
| gold | prodcode | 37745 | anxiolytic |
| gold | prodcode | 38274 | antidepressant |
| gold | prodcode | 38418 | anxiolytic |
| gold | prodcode | 38424 | anxiolytic |
| gold | prodcode | 38827 | antidepressant |
| gold | prodcode | 38890 | antidepressant |
| gold | prodcode | 39145 | antidepressant |
| gold | prodcode | 39284 | anxiolytic |
| gold | prodcode | 39359 | antidepressant |
| gold | prodcode | 39360 | antidepressant |
| gold | prodcode | 39770 | antidepressant |
| gold | prodcode | 39809 | antidepressant |
| gold | prodcode | 40048 | antidepressant |
| gold | prodcode | 40049 | antidepressant |
| gold | prodcode | 40054 | antidepressant |
| gold | prodcode | 40059 | antidepressant |
| gold | prodcode | 40062 | antidepressant |
| gold | prodcode | 40092 | antidepressant |
| gold | prodcode | 40153 | anxiolytic |
| gold | prodcode | 40160 | antidepressant |
| gold | prodcode | 40165 | antidepressant |
| gold | prodcode | 40277 | antidepressant |
| gold | prodcode | 40295 | antidepressant |
| gold | prodcode | 40396 | antidepressant |
| gold | prodcode | 40407 | antidepressant |
| gold | prodcode | 40494 | antidepressant |
| gold | prodcode | 40514 | antidepressant |
| gold | prodcode | 40515 | antidepressant |
| gold | prodcode | 40517 | antidepressant |
| gold | prodcode | 40726 | antidepressant |
| gold | prodcode | 40764 | antidepressant |

| | | | |
|---|---|---|---|
| gold | prodcode | 40777 | antidepressant |
| gold | prodcode | 40815 | antidepressant |
| gold | prodcode | 40817 | antidepressant |
| gold | prodcode | 40892 | antidepressant |
| gold | prodcode | 40917 | antidepressant |
| gold | prodcode | 41033 | antidepressant |
| gold | prodcode | 41062 | antidepressant |
| gold | prodcode | 41299 | antidepressant |
| gold | prodcode | 41314 | antidepressant |
| gold | prodcode | 41385 | anxiolytic |
| gold | prodcode | 41391 | anxiolytic |
| gold | prodcode | 41408 | antidepressant |
| gold | prodcode | 41516 | anxiolytic |
| gold | prodcode | 41528 | antidepressant |
| gold | prodcode | 41539 | anxiolytic |
| gold | prodcode | 41542 | anxiolytic |
| gold | prodcode | 41562 | anxiolytic |
| gold | prodcode | 41563 | antidepressant |
| gold | prodcode | 41597 | antidepressant |
| gold | prodcode | 41601 | anxiolytic |
| gold | prodcode | 41609 | antidepressant |
| gold | prodcode | 41627 | antidepressant |
| gold | prodcode | 41628 | antidepressant |
| gold | prodcode | 41653 | anxiolytic |
| gold | prodcode | 41654 | antidepressant |
| gold | prodcode | 41681 | antidepressant |
| gold | prodcode | 41696 | anxiolytic |
| gold | prodcode | 41697 | anxiolytic |
| gold | prodcode | 41709 | antidepressant |
| gold | prodcode | 41710 | antidepressant |
| gold | prodcode | 41717 | anxiolytic |
| gold | prodcode | 41718 | anxiolytic |
| gold | prodcode | 41729 | antidepressant |
| gold | prodcode | 41731 | antidepressant |
| gold | prodcode | 41747 | antidepressant |
| gold | prodcode | 42078 | antidepressant |
| gold | prodcode | 42089 | anxiolytic |
| gold | prodcode | 42107 | antidepressant |
| gold | prodcode | 42228 | antidepressant |
| gold | prodcode | 42247 | antidepressant |
| gold | prodcode | 42387 | antidepressant |
| gold | prodcode | 42394 | antidepressant |
| gold | prodcode | 42499 | antidepressant |
| gold | prodcode | 42600 | antidepressant |
| gold | prodcode | 42660 | antidepressant |
| gold | prodcode | 42734 | antidepressant |
| gold | prodcode | 42803 | antidepressant |
| gold | prodcode | 42814 | anxiolytic |
| gold | prodcode | 43024 | antidepressant |

| | | | |
|---|---|---|---|
| gold | prodcode | 43203 | antidepressant |
| gold | prodcode | 43234 | antidepressant |
| gold | prodcode | 43235 | antidepressant |
| gold | prodcode | 43236 | antidepressant |
| gold | prodcode | 43237 | antidepressant |
| gold | prodcode | 43239 | antidepressant |
| gold | prodcode | 43240 | anxiolytic |
| gold | prodcode | 43241 | antidepressant |
| gold | prodcode | 43242 | antidepressant |
| gold | prodcode | 43246 | antidepressant |
| gold | prodcode | 43247 | antidepressant |
| gold | prodcode | 43248 | antidepressant |
| gold | prodcode | 43250 | antidepressant |
| gold | prodcode | 43253 | antidepressant |
| gold | prodcode | 43256 | antidepressant |
| gold | prodcode | 43257 | antidepressant |
| gold | prodcode | 43334 | antidepressant |
| gold | prodcode | 43445 | anxiolytic |
| gold | prodcode | 43518 | antidepressant |
| gold | prodcode | 43519 | antidepressant |
| gold | prodcode | 43534 | antidepressant |
| gold | prodcode | 43560 | anxiolytic |
| gold | prodcode | 43561 | antidepressant |
| gold | prodcode | 43673 | antidepressant |
| gold | prodcode | 43968 | antidepressant |
| gold | prodcode | 44853 | antidepressant |
| gold | prodcode | 44861 | antidepressant |
| gold | prodcode | 44936 | antidepressant |
| gold | prodcode | 44937 | antidepressant |
| gold | prodcode | 44944 | antidepressant |
| gold | prodcode | 45135 | anxiolytic |
| gold | prodcode | 45223 | antidepressant |
| gold | prodcode | 45224 | antidepressant |
| gold | prodcode | 45226 | antidepressant |
| gold | prodcode | 45233 | antidepressant |
| gold | prodcode | 45242 | antidepressant |
| gold | prodcode | 45247 | antidepressant |
| gold | prodcode | 45254 | anxiolytic |
| gold | prodcode | 45275 | anxiolytic |
| gold | prodcode | 45283 | anxiolytic |
| gold | prodcode | 45286 | antidepressant |
| gold | prodcode | 45304 | antidepressant |
| gold | prodcode | 45316 | antidepressant |
| gold | prodcode | 45318 | antidepressant |
| gold | prodcode | 45329 | antidepressant |
| gold | prodcode | 45350 | antidepressant |
| gold | prodcode | 45353 | anxiolytic |
| gold | prodcode | 45664 | antidepressant |
| gold | prodcode | 45737 | antidepressant |

| | | | |
|---|---|---|---|
| gold | prodcode | 45806 | antidepressant |
| gold | prodcode | 45818 | antidepressant |
| gold | prodcode | 45829 | anxiolytic |
| gold | prodcode | 45915 | antidepressant |
| gold | prodcode | 45959 | antidepressant |
| gold | prodcode | 46078 | anxiolytic |
| gold | prodcode | 46668 | antidepressant |
| gold | prodcode | 46799 | anxiolytic |
| gold | prodcode | 46801 | antidepressant |
| gold | prodcode | 46818 | antidepressant |
| gold | prodcode | 46847 | anxiolytic |
| gold | prodcode | 46896 | anxiolytic |
| gold | prodcode | 46926 | antidepressant |
| gold | prodcode | 46939 | anxiolytic |
| gold | prodcode | 46953 | anxiolytic |
| gold | prodcode | 46964 | anxiolytic |
| gold | prodcode | 46970 | antidepressant |
| gold | prodcode | 46977 | antidepressant |
| gold | prodcode | 47363 | antidepressant |
| gold | prodcode | 47945 | antidepressant |
| gold | prodcode | 47966 | antidepressant |
| gold | prodcode | 48026 | antidepressant |
| gold | prodcode | 48045 | antidepressant |
| gold | prodcode | 48065 | antidepressant |
| gold | prodcode | 48185 | antidepressant |
| gold | prodcode | 48199 | antidepressant |
| gold | prodcode | 48216 | antidepressant |
| gold | prodcode | 48220 | antidepressant |
| gold | prodcode | 48517 | anxiolytic |
| gold | prodcode | 48698 | antidepressant |
| gold | prodcode | 49165 | antidepressant |
| gold | prodcode | 49504 | anxiolytic |
| gold | prodcode | 49511 | antidepressant |
| gold | prodcode | 49519 | antidepressant |
| gold | prodcode | 49589 | anxiolytic |
| gold | prodcode | 49820 | antidepressant |
| gold | prodcode | 50081 | antidepressant |
| gold | prodcode | 50722 | antidepressant |
| gold | prodcode | 50892 | antidepressant |
| gold | prodcode | 50934 | antidepressant |
| gold | prodcode | 51280 | antidepressant |
| gold | prodcode | 51361 | antidepressant |
| gold | prodcode | 51383 | antidepressant |
| gold | prodcode | 51699 | antidepressant |
| gold | prodcode | 51758 | antidepressant |
| gold | prodcode | 52022 | anxiolytic |
| gold | prodcode | 52074 | antidepressant |
| gold | prodcode | 52100 | antidepressant |
| gold | prodcode | 52354 | antidepressant |

| | | | |
|---|---|---|---|
| gold | prodcode | 52408 | antidepressant |
| gold | prodcode | 52516 | antidepressant |
| gold | prodcode | 52607 | antidepressant |
| gold | prodcode | 52716 | antidepressant |
| gold | prodcode | 52824 | antidepressant |
| gold | prodcode | 52867 | antidepressant |
| gold | prodcode | 53161 | antidepressant |
| gold | prodcode | 53187 | antidepressant |
| gold | prodcode | 53321 | antidepressant |
| gold | prodcode | 53326 | antidepressant |
| gold | prodcode | 53394 | antidepressant |
| gold | prodcode | 53543 | antidepressant |
| gold | prodcode | 53648 | antidepressant |
| gold | prodcode | 53699 | antidepressant |
| gold | prodcode | 53787 | antidepressant |
| gold | prodcode | 53808 | antidepressant |
| gold | prodcode | 54012 | antidepressant |
| gold | prodcode | 54081 | antidepressant |
| gold | prodcode | 54342 | antidepressant |
| gold | prodcode | 54644 | antidepressant |
| gold | prodcode | 54686 | antidepressant |
| gold | prodcode | 54695 | anxiolytic |
| gold | prodcode | 54747 | antidepressant |
| gold | prodcode | 54792 | antidepressant |
| gold | prodcode | 54826 | antidepressant |
| gold | prodcode | 54827 | antidepressant |
| gold | prodcode | 54877 | antidepressant |
| gold | prodcode | 54933 | antidepressant |
| gold | prodcode | 55023 | antidepressant |
| gold | prodcode | 55033 | antidepressant |
| gold | prodcode | 55137 | antidepressant |
| gold | prodcode | 55138 | antidepressant |
| gold | prodcode | 55139 | antidepressant |
| gold | prodcode | 55146 | antidepressant |
| gold | prodcode | 55289 | antidepressant |
| gold | prodcode | 55424 | antidepressant |
| gold | prodcode | 55482 | antidepressant |
| gold | prodcode | 55488 | antidepressant |
| gold | prodcode | 55491 | antidepressant |
| gold | prodcode | 55501 | antidepressant |
| gold | prodcode | 55537 | antidepressant |
| gold | prodcode | 55836 | anxiolytic |
| gold | prodcode | 55970 | antidepressant |
| gold | prodcode | 56009 | antidepressant |
| gold | prodcode | 56209 | antidepressant |
| gold | prodcode | 56229 | antidepressant |
| gold | prodcode | 56292 | antidepressant |
| gold | prodcode | 56355 | antidepressant |
| gold | prodcode | 56457 | antidepressant |

| | | | |
|---|---|---|---|
| gold | prodcode | 56501 | antidepressant |
| gold | prodcode | 56551 | anxiolytic |
| gold | prodcode | 56662 | antidepressant |
| gold | prodcode | 56703 | antidepressant |
| gold | prodcode | 56811 | anxiolytic |
| gold | prodcode | 56927 | anxiolytic |
| gold | prodcode | 57107 | antidepressant |
| gold | prodcode | 57226 | antidepressant |
| gold | prodcode | 57268 | anxiolytic |
| gold | prodcode | 57532 | antidepressant |
| gold | prodcode | 57751 | antidepressant |
| gold | prodcode | 57926 | antidepressant |
| gold | prodcode | 57936 | antidepressant |
| gold | prodcode | 57937 | anxiolytic |
| gold | prodcode | 57972 | antidepressant |
| gold | prodcode | 57978 | antidepressant |
| gold | prodcode | 58291 | antidepressant |
| gold | prodcode | 58450 | antidepressant |
| gold | prodcode | 58476 | antidepressant |
| gold | prodcode | 58625 | antidepressant |
| gold | prodcode | 58664 | antidepressant |
| gold | prodcode | 58681 | antidepressant |
| gold | prodcode | 58723 | antidepressant |
| gold | prodcode | 58726 | antidepressant |
| gold | prodcode | 58837 | antidepressant |
| gold | prodcode | 59035 | antidepressant |
| gold | prodcode | 59095 | anxiolytic |
| gold | prodcode | 59161 | antidepressant |
| gold | prodcode | 59170 | anxiolytic |
| gold | prodcode | 59193 | antidepressant |
| gold | prodcode | 59288 | antidepressant |
| gold | prodcode | 59358 | antidepressant |
| gold | prodcode | 59563 | antidepressant |
| gold | prodcode | 59600 | antidepressant |
| gold | prodcode | 59640 | anxiolytic |
| gold | prodcode | 59650 | antidepressant |
| gold | prodcode | 59694 | antidepressant |
| gold | prodcode | 59753 | antidepressant |
| gold | prodcode | 59820 | antidepressant |
| gold | prodcode | 59923 | antidepressant |
| gold | prodcode | 59931 | antidepressant |
| gold | prodcode | 59953 | antidepressant |
| gold | prodcode | 59954 | antidepressant |
| gold | prodcode | 60138 | antidepressant |
| gold | prodcode | 60355 | antidepressant |
| gold | prodcode | 60370 | antidepressant |
| gold | prodcode | 60410 | antidepressant |
| gold | prodcode | 60449 | antidepressant |
| gold | prodcode | 60534 | antidepressant |

| | | | |
|---|---|---|---|
| gold | prodcode | 60538 | antidepressant |
| gold | prodcode | 60549 | antidepressant |
| gold | prodcode | 60568 | antidepressant |
| gold | prodcode | 60591 | antidepressant |
| gold | prodcode | 60619 | antidepressant |
| gold | prodcode | 60825 | anxiolytic |
| gold | prodcode | 60839 | antidepressant |
| gold | prodcode | 60843 | antidepressant |
| gold | prodcode | 60888 | antidepressant |
| gold | prodcode | 60895 | antidepressant |
| gold | prodcode | 60962 | antidepressant |
| gold | prodcode | 61236 | antidepressant |
| gold | prodcode | 61335 | antidepressant |
| gold | prodcode | 61443 | anxiolytic |
| gold | prodcode | 61450 | anxiolytic |
| gold | prodcode | 61503 | antidepressant |
| gold | prodcode | 61547 | antidepressant |
| gold | prodcode | 61657 | antidepressant |
| gold | prodcode | 61835 | antidepressant |
| gold | prodcode | 61842 | antidepressant |
| gold | prodcode | 61856 | antidepressant |
| gold | prodcode | 61886 | anxiolytic |
| gold | prodcode | 62155 | antidepressant |
| gold | prodcode | 62335 | antidepressant |
| gold | prodcode | 62427 | antidepressant |
| gold | prodcode | 62620 | antidepressant |
| gold | prodcode | 62645 | anxiolytic |
| gold | prodcode | 62681 | antidepressant |
| gold | prodcode | 62688 | antidepressant |
| gold | prodcode | 62692 | antidepressant |
| gold | prodcode | 62693 | antidepressant |
| gold | prodcode | 62734 | antidepressant |
| gold | prodcode | 62819 | antidepressant |
| gold | prodcode | 62927 | antidepressant |
| gold | prodcode | 62950 | antidepressant |
| gold | prodcode | 63216 | antidepressant |
| gold | prodcode | 63268 | antidepressant |
| gold | prodcode | 63276 | antidepressant |
| gold | prodcode | 63370 | antidepressant |
| gold | prodcode | 63403 | antidepressant |
| gold | prodcode | 63441 | antidepressant |
| gold | prodcode | 63481 | antidepressant |
| gold | prodcode | 63592 | anxiolytic |
| gold | prodcode | 63665 | anxiolytic |
| gold | prodcode | 63674 | anxiolytic |
| gold | prodcode | 63763 | antidepressant |
| gold | prodcode | 63827 | antidepressant |
| gold | prodcode | 63859 | antidepressant |
| gold | prodcode | 63916 | antidepressant |

| | | | |
|---|---|---|---|
| gold | prodcode | 63953 | antidepressant |
| gold | prodcode | 64000 | antidepressant |
| gold | prodcode | 64101 | antidepressant |
| gold | prodcode | 64139 | antidepressant |
| gold | prodcode | 64141 | antidepressant |
| gold | prodcode | 64223 | antidepressant |
| gold | prodcode | 64330 | antidepressant |
| gold | prodcode | 64423 | antidepressant |
| gold | prodcode | 64442 | antidepressant |
| gold | prodcode | 64458 | antidepressant |
| gold | prodcode | 64647 | antidepressant |
| gold | prodcode | 64729 | anxiolytic |
| gold | prodcode | 64775 | anxiolytic |
| gold | prodcode | 64785 | antidepressant |
| gold | prodcode | 64876 | anxiolytic |
| gold | prodcode | 65152 | antidepressant |
| gold | prodcode | 65165 | antidepressant |
| gold | prodcode | 65190 | anxiolytic |
| gold | prodcode | 65213 | antidepressant |
| gold | prodcode | 65237 | antidepressant |
| gold | prodcode | 65439 | antidepressant |
| gold | prodcode | 65445 | antidepressant |
| gold | prodcode | 65482 | antidepressant |
| gold | prodcode | 65483 | antidepressant |
| gold | prodcode | 65555 | antidepressant |
| gold | prodcode | 65618 | antidepressant |
| gold | prodcode | 65637 | anxiolytic |
| gold | prodcode | 65666 | antidepressant |
| gold | prodcode | 65738 | antidepressant |
| gold | prodcode | 65762 | antidepressant |
| gold | prodcode | 65771 | antidepressant |
| gold | prodcode | 65804 | antidepressant |
| gold | prodcode | 65809 | antidepressant |
| gold | prodcode | 65879 | antidepressant |
| gold | prodcode | 65888 | antidepressant |
| gold | prodcode | 65892 | antidepressant |
| gold | prodcode | 65899 | antidepressant |
| gold | prodcode | 65987 | antidepressant |
| gold | prodcode | 66100 | antidepressant |
| gold | prodcode | 66183 | antidepressant |
| gold | prodcode | 66201 | antidepressant |
| gold | prodcode | 66292 | antidepressant |
| gold | prodcode | 66405 | antidepressant |
| gold | prodcode | 66412 | antidepressant |
| gold | prodcode | 66413 | antidepressant |
| gold | prodcode | 66437 | antidepressant |
| gold | prodcode | 66493 | antidepressant |
| gold | prodcode | 66560 | antidepressant |
| gold | prodcode | 66572 | antidepressant |

| | | | |
|---|---|---|---|
| gold | prodcode | 66578 | antidepressant |
| gold | prodcode | 66579 | antidepressant |
| gold | prodcode | 66580 | antidepressant |
| gold | prodcode | 66744 | antidepressant |
| gold | prodcode | 66749 | antidepressant |
| gold | prodcode | 66752 | antidepressant |
| gold | prodcode | 66890 | antidepressant |
| gold | prodcode | 66919 | antidepressant |
| gold | prodcode | 67092 | antidepressant |
| gold | prodcode | 67097 | antidepressant |
| gold | prodcode | 67127 | antidepressant |
| gold | prodcode | 67259 | antidepressant |
| gold | prodcode | 67271 | antidepressant |
| gold | prodcode | 67272 | antidepressant |
| gold | prodcode | 67288 | antidepressant |
| gold | prodcode | 67305 | antidepressant |
| gold | prodcode | 67431 | antidepressant |
| gold | prodcode | 67496 | antidepressant |
| gold | prodcode | 67562 | antidepressant |
| gold | prodcode | 67563 | antidepressant |
| gold | prodcode | 67564 | antidepressant |
| gold | prodcode | 67728 | antidepressant |
| gold | prodcode | 67730 | antidepressant |
| gold | prodcode | 67736 | antidepressant |
| gold | prodcode | 67742 | antidepressant |
| gold | prodcode | 67757 | antidepressant |
| gold | prodcode | 67758 | antidepressant |
| gold | prodcode | 67769 | antidepressant |
| gold | prodcode | 67874 | antidepressant |
| gold | prodcode | 67888 | antidepressant |
| gold | prodcode | 67928 | antidepressant |
| gold | prodcode | 67935 | antidepressant |
| gold | prodcode | 67990 | antidepressant |
| gold | prodcode | 68050 | antidepressant |
| gold | prodcode | 68052 | antidepressant |
| gold | prodcode | 68096 | antidepressant |
| gold | prodcode | 68228 | antidepressant |
| gold | prodcode | 68266 | antidepressant |
| gold | prodcode | 68325 | antidepressant |
| gold | prodcode | 68544 | antidepressant |
| gold | prodcode | 68657 | antidepressant |
| gold | prodcode | 68665 | antidepressant |
| gold | prodcode | 68680 | antidepressant |
| gold | prodcode | 68756 | antidepressant |
| gold | prodcode | 68876 | antidepressant |
| gold | prodcode | 68933 | antidepressant |
| gold | prodcode | 69005 | antidepressant |
| gold | prodcode | 69317 | antidepressant |
| gold | prodcode | 69355 | antidepressant |

| | | | |
|---|---|---|---|
| gold | prodcode | 69420 | antidepressant |
| gold | prodcode | 69428 | antidepressant |
| gold | prodcode | 69525 | antidepressant |
| gold | prodcode | 69542 | antidepressant |
| gold | prodcode | 69571 | antidepressant |
| gold | prodcode | 69685 | antidepressant |
| gold | prodcode | 69712 | antidepressant |
| gold | prodcode | 69725 | antidepressant |
| gold | prodcode | 69726 | antidepressant |
| gold | prodcode | 69752 | antidepressant |
| gold | prodcode | 69819 | antidepressant |
| gold | prodcode | 69898 | antidepressant |
| gold | prodcode | 69941 | antidepressant |
| gold | prodcode | 69965 | antidepressant |
| gold | prodcode | 69991 | antidepressant |
| gold | prodcode | 69992 | antidepressant |
| gold | prodcode | 70063 | antidepressant |
| gold | prodcode | 70287 | antidepressant |
| gold | prodcode | 70300 | antidepressant |
| gold | prodcode | 70315 | antidepressant |
| gold | prodcode | 70353 | antidepressant |
| gold | prodcode | 70405 | antidepressant |
| gold | prodcode | 70420 | antidepressant |
| gold | prodcode | 70495 | antidepressant |
| gold | prodcode | 70521 | antidepressant |
| gold | prodcode | 70593 | antidepressant |
| gold | prodcode | 70728 | antidepressant |
| gold | prodcode | 70790 | antidepressant |
| gold | prodcode | 70806 | antidepressant |
| gold | prodcode | 70838 | antidepressant |
| gold | prodcode | 70931 | antidepressant |
| gold | prodcode | 70991 | antidepressant |
| gold | prodcode | 71005 | antidepressant |
| gold | prodcode | 71023 | antidepressant |
| gold | prodcode | 71031 | antidepressant |
| gold | prodcode | 71042 | antidepressant |
| gold | prodcode | 71059 | antidepressant |
| gold | prodcode | 71067 | antidepressant |
| gold | prodcode | 71253 | antidepressant |
| gold | prodcode | 71257 | antidepressant |
| gold | prodcode | 71543 | antidepressant |
| gold | prodcode | 71669 | antidepressant |
| gold | prodcode | 71782 | antidepressant |
| gold | prodcode | 71806 | antidepressant |
| gold | prodcode | 71848 | antidepressant |
| gold | prodcode | 71852 | antidepressant |
| gold | prodcode | 71932 | antidepressant |
| gold | prodcode | 72124 | antidepressant |
| gold | prodcode | 72211 | antidepressant |

| | | | |
|---|---|---|---|
| gold | prodcode | 72291 | antidepressant |
| gold | prodcode | 72373 | antidepressant |
| gold | prodcode | 72626 | antidepressant |
| gold | prodcode | 72773 | antidepressant |
| gold | prodcode | 73298 | antidepressant |
| gold | prodcode | 73363 | antidepressant |
| gold | prodcode | 73414 | antidepressant |
| gold | prodcode | 73417 | antidepressant |
| gold | prodcode | 73419 | antidepressant |
| gold | prodcode | 73540 | antidepressant |
| gold | prodcode | 73589 | antidepressant |
| gold | prodcode | 73636 | antidepressant |
| gold | prodcode | 73639 | antidepressant |
| gold | prodcode | 73658 | antidepressant |
| gold | prodcode | 73667 | antidepressant |
| gold | prodcode | 73668 | antidepressant |
| gold | prodcode | 73759 | antidepressant |
| gold | prodcode | 73868 | antidepressant |
| gold | prodcode | 73962 | antidepressant |
| gold | prodcode | 74010 | antidepressant |
| gold | prodcode | 74011 | antidepressant |
| gold | prodcode | 74190 | antidepressant |
| gold | prodcode | 74516 | antidepressant |
| gold | prodcode | 74557 | antidepressant |
| gold | prodcode | 74586 | antidepressant |
| gold | prodcode | 74588 | antidepressant |
| gold | prodcode | 74753 | antidepressant |
| gold | prodcode | 74774 | antidepressant |
| gold | prodcode | 74785 | antidepressant |
| gold | prodcode | 74858 | antidepressant |
| gold | prodcode | 74886 | antidepressant |
| gold | prodcode | 74907 | antidepressant |
| gold | prodcode | 74993 | antidepressant |
| gold | prodcode | 75054 | antidepressant |
| gold | prodcode | 75068 | antidepressant |
| gold | prodcode | 75075 | antidepressant |
| gold | prodcode | 75247 | antidepressant |
| gold | prodcode | 75263 | antidepressant |
| gold | prodcode | 75405 | antidepressant |
| gold | prodcode | 75525 | antidepressant |
| gold | prodcode | 75645 | antidepressant |
| gold | prodcode | 75688 | antidepressant |
| gold | prodcode | 75697 | antidepressant |
| gold | prodcode | 75702 | antidepressant |
| gold | prodcode | 75799 | antidepressant |
| gold | prodcode | 75848 | antidepressant |
| gold | prodcode | 75894 | antidepressant |
| gold | prodcode | 75943 | antidepressant |
| gold | prodcode | 75952 | antidepressant |

# Supplement C Supplemental tables primary care-recorded CMD, CPRD

Supplement C table 1 Extended primary care sample characteristics with cases and person-years: 2009, 2014, 2019, CPRD

Supplement C table 2 Primary care-recorded CMD, incidence (per 1,000 person-years) and 95%CI, 2009-2019, CPRD

Supplement C table 3 Primary care-recorded CMD over time, incidence rate ratios from 2009 to 2014; 2014 to 2019, and 2009 to 2019, CPRD

Supplement C table 4 Incidence rate ratios of primary care-recorded CMD by sociodemographic group, 2009-2019, CPRD

**Supplement C table 1 Extended primary care sample characteristics with cases and person-years: 2009, 2014, 2019, CPRD**

| | 2009 | | | | | 2014 | | | | | 2019 | | | | |
|---|---|---|---|---|---|---|---|---|---|---|---|---|---|---|---|
| | **n** | **%** | **Cases** | **%** | **PY** | **n** | **%** | **Cases** | **%** | **PY** | **n** | **%** | **Cases** | **%** | **PY** |
| Total | | | | | | | | | | | | | | | |
| | 2,179,380 | 100 | 117,893 | 5.41 | 1,732,370 | 3,061,418 | 100 | 195,756 | 6.39 | 2,419,918 | 3,935,301 | 100 | 281,258 | 7.15 | 3,183,874 |
| Sex | | | | | | | | | | | | | | | |
| Female | 1,144,514 | 52.5 | 78,949 | 6.9 | 883,163 | 1,614,301 | 52.73 | 127,213 | 7.88 | 1,238,635 | 2,034,723 | 51.7 | 177,253 | 8.71 | 1,601,960 |
| Male | 1,034,866 | 47.5 | 38,944 | 3.8 | 849,207 | 1,447,117 | 47.27 | 68,543 | 4.74 | 1,181,283 | 1,900,578 | 48.3 | 104,005 | 5.47 | 1,581,914 |
| Age group | | | | | | | | | | | | | | | |
| 16-19 | 169,508 | 7.78 | 8,772 | 5.2 | 101,778 | 163,530 | 5.34 | 10,699 | 6.54 | 97,054 | 158,905 | 4.04 | 13,499 | 8.5 | 93,571 |
| 20-24 | 702,825 | 32.25 | 40,456 | 5.76 | 528,896 | 691,587 | 22.59 | 44,315 | 6.41 | 515,204 | 680,305 | 17.29 | 51,686 | 7.6 | 510,600 |
| 25-29 | 1,307,047 | 59.97 | 68,665 | 5.25 | 1,101,696 | 925,879 | 30.24 | 62,926 | 6.8 | 724,881 | 877,798 | 22.31 | 65,109 | 7.42 | 694,700 |
| 30-34 | - | - | - | - | - | 1,280,422 | 41.82 | 77,816 | 6.08 | 1,082,779 | 1,018,290 | 25.88 | 74,095 | 7.28 | 839,986 |
| 35-39 | - | - | - | - | - | - | - | - | - | - | 1,200,003 | 30.49 | 76,869 | 6.41 | 1,045,018 |
| Cohort | | | | | | | | | | | | | | | |
| 1980-84 | 1,328,153 | 60.94 | 75,946 | 5.72 | 1,106,864 | 1,304,076 | 42.6 | 86,372 | 6.62 | 1,088,448 | 1,219,774 | 31 | 85,808 | 7.03 | 1,049,482 |
| 1985-89 | 687,754 | 31.56 | 35,728 | 5.19 | 525,185 | 921,962 | 30.12 | 59,653 | 6.47 | 723,833 | 1,016,325 | 25.83 | 72,250 | 7.11 | 839,524 |
| 1990-94 | 163,473 | 7.5 | 6,219 | 3.8 | 100,321 | 678,220 | 22.15 | 42,068 | 6.2 | 512,022 | 879,112 | 22.34 | 63,633 | 7.24 | 695,034 |
| 1995-99 | - | - | - | - | - | 157,160 | 5.13 | 7,663 | 4.88 | 95,615 | 668,104 | 16.98 | 50,255 | 7.52 | 507,793 |
| 2000-03 | - | - | - | - | - | - | - | - | - | - | 151,986 | 3.86 | 9,312 | 6.13 | 92,041 |
| Ethnicity | | | | | | | | | | | | | | | |
| Asian | 192,098 | 13.92 | 6,016 | 3.13 | 145,743 | 340,259 | 15.76 | 11,707 | 3.44 | 271,663 | 461,591 | 16.1 | 20,251 | 4.39 | 378,113 |
| Black | 76,413 | 5.54 | 3,308 | 4.33 | 60,200 | 119,025 | 5.5 | 5,905 | 4.96 | 95,988 | 160,523 | 5.6 | 9,734 | 6.06 | 131,364 |
| Mixed | 51,072 | 3.7 | 2,680 | 5.25 | 38,622 | 88,510 | 4.1 | 5,205 | 5.88 | 69,043 | 139,285 | 4.86 | 9,707 | 6.97 | 110,407 |
| Other | 26,924 | 1.95 | 977 | 3.63 | 20,077 | 47,058 | 2.2 | 1,938 | 4.12 | 36,481 | 76,870 | 2.68 | 3,650 | 4.75 | 61,864 |
| White | 1,033,148 | 74.88 | 74,323 | 7.19 | 790,645 | 1,564,471 | 72.45 | 123,190 | 7.87 | 1,224,082 | 2,028,898 | 70.76 | 172,983 | 8.53 | 1,623,478 |
| Country | | | | | | | | | | | | | | | |
| England | 1,882,792 | 86.39 | 101,197 | 5.37 | 1,490,553 | 2,649,545 | 86.55 | 168,560 | 6.36 | 2,083,265 | 3,413,569 | 86.74 | 244,592 | 7.17 | 2,753,525 |
| Scotland | 162,414 | 7.45 | 8,557 | 5.27 | 131,049 | 230,273 | 7.52 | 14,140 | 6.14 | 187,198 | 297,645 | 7.56 | 19,010 | 6.39 | 244,215 |
| Northern Ireland | 31,305 | 1.44 | 2,119 | 6.77 | 27,192 | 40,106 | 1.31 | 3,054 | 7.61 | 34,455 | 50,927 | 1.29 | 3,872 | 7.6 | 43,668 |
| Wales | 102,869 | 4.72 | 6,020 | 5.85 | 83,576 | 141,494 | 4.62 | 10,002 | 7.07 | 115,000 | 173,160 | 4.4 | 13,784 | 7.96 | 142,467 |
| Region † | | | | | | | | | | | | | | | |
| North East | 54,434 | 2.5 | 3,823 | 7.02 | 43,257 | 76,163 | 2.49 | 5,880 | 7.72 | 58,361 | 92,089 | 2.34 | 7,958 | 8.64 | 72,323 |
| North West | 305,487 | 14.02 | 18,931 | 6.2 | 243,767 | 406,230 | 13.27 | 30,661 | 7.55 | 321,731 | 541,028 | 13.75 | 46,407 | 8.58 | 436,862 |
| Yorksh. & the Humber | 84,607 | 3.88 | 4,167 | 4.93 | 66,159 | 108,575 | 3.55 | 6,689 | 6.16 | 83,564 | 126,587 | 3.22 | 9,404 | 7.43 | 101,038 |
| East Midlands | 85,335 | 3.92 | 3,685 | 4.32 | 65,765 | 92,306 | 3.02 | 4,717 | 5.11 | 70,625 | 117,900 | 3 | 7,384 | 6.26 | 88,541 |
| West Midlands | 240,884 | 11.05 | 13,940 | 5.79 | 194,231 | 344,809 | 11.26 | 24,557 | 7.12 | 274,437 | 445,681 | 11.33 | 36,809 | 8.26 | 364,141 |
| East of England | 83,903 | 3.85 | 5,208 | 6.21 | 67,591 | 98,123 | 3.21 | 6,737 | 6.87 | 78,484 | 103,118 | 2.62 | 7,492 | 7.27 | 83,147 |
| London | 431,105 | 19.78 | 18,016 | 4.18 | 338,501 | 711,831 | 23.25 | 34,032 | 4.78 | 560,363 | 973,897 | 24.75 | 51,397 | 5.28 | 795,168 |
| South East | 380,293 | 17.45 | 20,674 | 5.44 | 299,575 | 521,410 | 17.03 | 34,217 | 6.56 | 412,321 | 659,028 | 16.75 | 47,738 | 7.24 | 533,734 |
| South West | 216,744 | 9.95 | 12,753 | 5.88 | 171,707 | 290,098 | 9.48 | 21,070 | 7.26 | 223,378 | 354,241 | 9 | 30,003 | 8.47 | 278,572 |
| Deprivation † | | | | | | | | | | | | | | | |
| Fifth 1 (Least deprived) | 273,508 | 12.55 | 14,355 | 5.25 | 218,870 | 401,528 | 13.12 | 25,291 | 6.3 | 317,500 | 542,754 | 13.79 | 38,497 | 7.09 | 436,050 |
| Fifth 2 | 395,052 | 18.13 | 19,340 | 4.9 | 312,553 | 554,444 | 18.11 | 32,545 | 5.87 | 435,448 | 716,215 | 18.2 | 48,463 | 6.77 | 576,555 |
| Fifth 3 | 401,383 | 18.42 | 21,018 | 5.24 | 320,101 | 557,730 | 18.22 | 34,435 | 6.17 | 442,198 | 705,587 | 17.93 | 49,106 | 6.96 | 574,852 |
| Fifth 4 | 573,390 | 26.31 | 29,875 | 5.21 | 450,943 | 804,201 | 26.27 | 49,947 | 6.21 | 631,674 | 1,029,073 | 26.15 | 71,844 | 6.98 | 828,953 |
| Fifth (Most deprived) | 536,047 | 24.6 | 33,305 | 6.21 | 429,903 | 743,515 | 24.29 | 53,538 | 7.2 | 593,098 | 941,672 | 23.93 | 73,348 | 7.79 | 767,464 |

† England only

**Supplement C table 2** **Primary care-recorded CMD, incidence (per 1,000 person-years) and 95%CI, 2009-2019, CPRD**

| Variable | 2009 | | | | | 2010 | | | | | 2011 | | | | |
|---|---|---|---|---|---|---|---|---|---|---|---|---|---|---|---|
| | Cases | PY | IR | 95% CI | | Cases | PY | IR | 95% CI | | Cases | PY | IR | 95% CI | |
| Total | | | | | | | | | | | | | | | |
| | 117,893 | 1,732,370 | 68.05 | 67.66 | 68.44 | 133,379 | 1,881,449 | 69.15 | 68.77 | 69.53 | 148,920 | 2,027,315 | 67.71 | 67.34 | 68.09 |
| Sex | | | | | | | | | | | | | | | |
| Female | 78,949 | 883,163 | 89.39 | 88.77 | 90.01 | 89,453 | 958,907 | 91.38 | 90.77 | 92.00 | 99,060 | 1,035,698 | 89.09 | 88.49 | 89.69 |
| Male | 38,944 | 849,207 | 45.86 | 45.40 | 46.31 | 43,926 | 922,542 | 46.40 | 45.96 | 46.85 | 49,860 | 991,618 | 46.26 | 45.83 | 46.70 |
| Age group | | | | | | | | | | | | | | | |
| 16-19 | 8,772 | 101,778 | 86.18 | 84.40 | 88.01 | 9,475 | 100,494 | 93.31 | 91.43 | 95.23 | 9,207 | 99,599 | 89.83 | 87.98 | 91.73 |
| 20-24 | 40,456 | 528,896 | 76.49 | 75.75 | 77.24 | 39,638 | 530,690 | 73.28 | 72.55 | 74.02 | 41,233 | 533,566 | 72.40 | 71.67 | 73.15 |
| 25-29 | 68,665 | 1,101,696 | 62.32 | 61.86 | 62.79 | 76,028 | 1,021,194 | 72.48 | 71.95 | 73.01 | 72,898 | 939,613 | 70.88 | 70.32 | 71.44 |
| 30-34 | - | - | - | - | - | 8,238 | 229,071 | 33.80 | 33.04 | 34.57 | 25,582 | 454,537 | 50.28 | 49.61 | 50.97 |
| 35-39 | - | - | - | - | - | - | - | - | - | - | - | - | - | - | - |
| Cohort | | | | | | | | | | | | | | | |
| 1980-84 | 75,946 | 1,106,864 | 68.61 | 68.12 | 69.10 | 79,279 | 1,118,659 | 68.85 | 68.36 | 69.34 | 82,425 | 1,122,277 | 66.57 | 66.07 | 67.07 |
| 1985-89 | 35,728 | 525,185 | 68.03 | 67.32 | 68.73 | 41,856 | 582,097 | 70.49 | 69.80 | 71.18 | 47,292 | 628,890 | 70.09 | 69.41 | 70.76 |
| 1990-94 | 6,219 | 100,321 | 61.99 | 60.47 | 63.55 | 12,244 | 180,692 | 66.74 | 65.55 | 67.95 | 19,023 | 272,095 | 67.25 | 66.27 | 68.25 |
| 1995-99 | - | - | - | - | - | - | - | - | - | - | 180 | 4,053 | 44.41 | 38.38 | 51.40 |
| 2000-03 | - | - | - | - | - | - | - | - | - | - | - | - | - | - | - |
| Ethnicity | | | | | | | | | | | | | | | |
| Asian | 6,016 | 145,743 | 41.28 | 40.25 | 42.33 | 7,186 | 182,833 | 38.09 | 37.20 | 39.00 | 8,796 | 216,923 | 37.11 | 36.30 | 37.94 |
| Black | 3,308 | 60,200 | 54.95 | 53.11 | 56.85 | 3,903 | 69,029 | 55.61 | 53.87 | 57.42 | 4,399 | 77,539 | 52.58 | 50.95 | 54.26 |
| Mixed | 2,680 | 38,622 | 69.39 | 66.81 | 72.06 | 3,340 | 46,445 | 70.65 | 68.24 | 73.13 | 3,733 | 53,252 | 65.75 | 63.55 | 68.03 |
| Other | 977 | 20,077 | 48.66 | 45.70 | 51.81 | 1,186 | 23,910 | 48.85 | 46.11 | 51.76 | 1,365 | 27,173 | 47.59 | 45.02 | 50.31 |
| White | 74,323 | 20,077 | 94.00 | 93.32 | 94.68 | 86,993 | 910,100 | 94.09 | 93.45 | 94.73 | 97,908 | 1,006,564 | 91.76 | 91.15 | 92.38 |
| Not stated | 30,589 | 677,082 | 45.18 | 44.67 | 45.68 | 30,771 | 649,132 | 45.53 | 45.01 | 46.06 | 32,719 | 645,863 | 44.76 | 44.23 | 45.29 |
| Country | | | | | | | | | | | | | | | |
| England | 101,197 | 1,490,553 | 67.89 | 67.47 | 68.31 | 114,420 | 1,622,018 | 68.87 | 68.46 | 69.28 | 128,079 | 1,748,187 | 67.78 | 67.38 | 68.18 |
| Scotland | 8,557 | 131,049 | 65.29 | 63.92 | 66.69 | 9,836 | 140,394 | 68.24 | 66.87 | 69.64 | 10,784 | 151,456 | 64.14 | 62.84 | 65.48 |
| Northern Ireland | 2,119 | 27,192 | 77.92 | 74.68 | 81.31 | 2,484 | 28,394 | 84.17 | 80.80 | 87.67 | 2,473 | 29,907 | 74.69 | 71.51 | 78.01 |
| Wales | 6,020 | 83,576 | 72.03 | 70.23 | 73.87 | 6,639 | 90,643 | 70.93 | 69.20 | 72.72 | 7,584 | 97,766 | 70.08 | 68.38 | 71.82 |
| Region † | | | | | | | | | | | | | | | |
| North East | 3,823 | 43,257 | 88.37 | 85.62 | 91.22 | 4,330 | 48,775 | 86.26 | 83.65 | 88.96 | 4,475 | 53,169 | 77.44 | 75.01 | 79.95 |
| North West | 18,931 | 243,767 | 77.66 | 76.56 | 78.77 | 21,353 | 261,280 | 80.00 | 78.91 | 81.11 | 23,433 | 278,784 | 77.97 | 76.90 | 79.05 |
| Yorksh. & Humb. | 4,167 | 66,159 | 62.98 | 61.10 | 64.92 | 4,664 | 69,823 | 65.48 | 63.59 | 67.43 | 5,282 | 72,270 | 67.44 | 65.52 | 69.42 |
| East Midlands | 3,685 | 66,159 | 56.03 | 54.25 | 57.87 | 3,884 | 67,397 | 56.53 | 54.75 | 58.38 | 4,206 | 68,227 | 57.55 | 55.74 | 59.43 |
| West Midlands | 13,940 | 194,231 | 71.77 | 70.58 | 72.97 | 15,845 | 207,956 | 74.47 | 73.29 | 75.67 | 18,102 | 222,952 | 75.17 | 74.00 | 76.36 |
| East of England | 5,208 | 67,591 | 77.05 | 74.98 | 79.17 | 5,710 | 71,249 | 77.89 | 75.84 | 80.00 | 5,944 | 74,297 | 73.02 | 71.03 | 75.07 |
| London | 18,016 | 338,501 | 53.22 | 52.45 | 54.00 | 21,199 | 389,867 | 53.07 | 52.34 | 53.80 | 24,422 | 437,505 | 51.95 | 51.27 | 52.65 |
| South East | 20,674 | 299,575 | 69.01 | 68.07 | 69.95 | 23,106 | 322,200 | 70.03 | 69.11 | 70.96 | 26,173 | 344,285 | 70.51 | 69.60 | 71.44 |
| South West | 12,753 | 171,707 | 74.27 | 72.99 | 75.57 | 14,329 | 322,200 | 76.31 | 75.04 | 77.61 | 16,042 | 196,699 | 75.53 | 74.28 | 76.80 |
| Deprivation † | | | | | | | | | | | | | | | |
| Fifth 1 (least deprived) | 14,355 | 218,870 | 65.58 | 64.52 | 66.67 | 16,196 | 238,090 | 66.30 | 65.26 | 67.36 | 18,565 | 257,640 | 66.48 | 65.46 | 67.52 |
| Fifth 2 | 19,340 | 312,553 | 61.87 | 61.01 | 62.75 | 21,750 | 337,238 | 62.91 | 62.06 | 63.77 | 24,222 | 361,609 | 61.79 | 60.96 | 62.63 |
| Fifth 3 | 21,018 | 320,101 | 65.66 | 64.78 | 66.55 | 24,100 | 337,238 | 67.58 | 66.71 | 68.46 | 26,747 | 373,408 | 66.05 | 65.20 | 66.91 |
| Fifth 4 | 29,875 | 450,943 | 66.25 | 65.50 | 67.00 | 34,115 | 494,255 | 67.44 | 66.71 | 68.18 | 38,081 | 532,770 | 65.76 | 65.05 | 66.48 |
| Fifth 5 (most deprived) | 33,305 | 429,903 | 77.47 | 76.64 | 78.30 | 37,218 | 464,446 | 78.19 | 77.37 | 79.01 | 41,305 | 501,889 | 76.05 | 75.26 | 76.84 |

PY: person-years; IR: incidence rate (per 1,000 person-years); 95% CI: 95% confidence intervals

† England only

**Supplement C table 2** **(pg 2) Primary care-recorded CMD, incidence (per 1,000 person-years) and 95%CI, 2009-2019, CPRD**

| Variable | 2012 | | | | | 2013 | | | | | 2014 | | | | |
|---|---|---|---|---|---|---|---|---|---|---|---|---|---|---|---|
| | Cases | PY | IR | 95% CI | | Cases | PY | IR | 95% CI | | Cases | PY | IR | 95% CI | |
| Total | | | | | | | | | | | | | | | |
| | 163,928 | 2,185,619 | 66.39 | 66.03 | 66.75 | 177,547 | 2,299,737 | 66.76 | 66.41 | 67.12 | 195,756 | 2,419,918 | 69.36 | 69.01 | 69.72 |
| Sex | | | | | | | | | | | | | | | |
| Female | 107,818 | 1,119,865 | 86.10 | 85.52 | 86.68 | 116,400 | 1,181,737 | 86.18 | 85.61 | 86.75 | 127,213 | 1,238,635 | 89.11 | 88.53 | 89.69 |
| Male | 56,110 | 1,065,754 | 46.81 | 46.38 | 47.23 | 61,147 | 1,118,000 | 47.58 | 47.16 | 48.00 | 68,543 | 1,181,283 | 50.18 | 49.75 | 50.61 |
| Age group | | | | | | | | | | | | | | | |
| 16-19 | 9,586 | 98,833 | 93.73 | 91.82 | 95.67 | 9,741 | 97,336 | 96.80 | 94.85 | 98.80 | 10,699 | 97,054 | 107.42 | 105.36 | 109.52 |
| 20-24 | 41,842 | 534,919 | 71.87 | 71.13 | 72.62 | 42,348 | 521,397 | 73.81 | 73.05 | 74.58 | 44,315 | 515,204 | 78.07 | 77.28 | 78.87 |
| 25-29 | 69,444 | 870,737 | 69.26 | 68.68 | 69.85 | 65,179 | 797,274 | 69.69 | 69.07 | 70.31 | 62,926 | 724,881 | 73.78 | 73.12 | 74.46 |
| 30-34 | 43,056 | 681,130 | 53.69 | 53.11 | 54.28 | 60,279 | 883,729 | 55.70 | 55.17 | 56.23 | 77,816 | 1,082,779 | 57.54 | 57.05 | 58.04 |
| 35-39 | - | - | - | - | - | - | - | - | - | - | - | - | - | - | - |
| Cohort | | | | | | | | | | | | | | | |
| 1980-84 | 84,594 | 1,129,125 | 64.01 | 63.51 | 64.50 | 85,362 | 1,107,869 | 63.22 | 62.72 | 63.73 | 86,372 | 1,088,448 | 63.61 | 63.09 | 64.13 |
| 1985-89 | 51,308 | 674,391 | 68.35 | 67.71 | 69.01 | 54,894 | 702,836 | 68.78 | 68.13 | 69.43 | 59,653 | 723,833 | 71.40 | 70.75 | 72.07 |
| 1990-94 | 27,049 | 364,967 | 70.20 | 69.33 | 71.09 | 34,382 | 446,780 | 71.65 | 70.84 | 72.46 | 42,068 | 512,022 | 75.74 | 74.96 | 76.53 |
| 1995-99 | 977 | 17,135 | 56.90 | 53.43 | 60.59 | 2,909 | 42,252 | 67.59 | 65.15 | 70.13 | 7,663 | 95,615 | 78.61 | 76.84 | 80.42 |
| 2000-03 | - | - | - | - | - | - | - | - | - | - | - | - | - | - | - |
| Ethnicity | | | | | | | | | | | | | | | |
| Asian | 9,774 | 244,742 | 35.45 | 34.69 | 36.22 | 10,591 | 260,119 | 35.33 | 34.60 | 36.09 | 11,707 | 271,663 | 36.56 | 35.82 | 37.31 |
| Black | 4,971 | 85,311 | 52.18 | 50.62 | 53.79 | 5,296 | 90,596 | 50.74 | 49.23 | 52.30 | 5,905 | 95,988 | 53.31 | 51.79 | 54.87 |
| Mixed | 4,409 | 59,709 | 67.74 | 65.59 | 69.95 | 4,710 | 63,966 | 66.04 | 63.98 | 68.17 | 5,205 | 69,043 | 66.89 | 64.87 | 68.98 |
| Other | 1,616 | 30,813 | 48.19 | 45.73 | 50.78 | 1,847 | 33,630 | 49.67 | 47.26 | 52.20 | 1,938 | 36,481 | 47.67 | 45.39 | 50.07 |
| White | 106,267 | 1,092,493 | 88.72 | 88.13 | 89.32 | 113,970 | 1,157,671 | 87.58 | 87.00 | 88.16 | 123,190 | 1,224,082 | 88.43 | 87.85 | 89.01 |
| Not stated | 36,891 | 672,552 | 45.93 | 45.40 | 46.47 | 41,133 | 693,755 | 49.27 | 48.72 | 49.82 | 47,811 | 722,661 | 55.51 | 54.94 | 56.09 |
| Country | | | | | | | | | | | | | | | |
| England | 141,090 | 1,887,091 | 66.70 | 66.31 | 67.08 | 152,902 | 1,982,344 | 67.29 | 66.91 | 67.68 | 168,560 | 2,083,265 | 70.09 | 69.71 | 70.48 |
| Scotland | 11,906 | 163,376 | 61.40 | 60.15 | 62.68 | 12,875 | 174,896 | 59.84 | 58.63 | 61.07 | 14,140 | 187,198 | 60.64 | 59.45 | 61.85 |
| Northern Ireland | 2,736 | 31,226 | 70.98 | 67.87 | 74.23 | 2,752 | 32,726 | 65.74 | 62.76 | 68.87 | 3,054 | 34,455 | 67.10 | 64.11 | 70.23 |
| Wales | 8,196 | 103,927 | 67.27 | 65.62 | 68.95 | 9,018 | 109,771 | 68.50 | 66.86 | 70.18 | 10,002 | 115,000 | 70.95 | 69.29 | 72.64 |
| Region † | | | | | | | | | | | | | | | |
| North East | 4,987 | 56,204 | 78.88 | 76.45 | 81.39 | 5,330 | 58,797 | 77.83 | 75.43 | 80.30 | 5,880 | 58,361 | 88.03 | 85.44 | 90.71 |
| North West | 25,342 | 294,749 | 76.51 | 75.45 | 77.57 | 27,766 | 306,471 | 79.03 | 77.97 | 80.11 | 30,661 | 321,731 | 82.62 | 81.55 | 83.72 |
| Yorksh. & Humb. | 5,663 | 75,907 | 66.03 | 64.14 | 67.96 | 6,054 | 78,540 | 67.59 | 65.69 | 69.54 | 6,689 | 83,564 | 69.16 | 67.28 | 71.09 |
| East Midlands | 4,352 | 71,306 | 54.84 | 53.09 | 56.65 | 4,405 | 70,127 | 55.68 | 53.88 | 57.53 | 4,717 | 70,625 | 59.22 | 57.36 | 61.13 |
| West Midlands | 19,917 | 241,541 | 74.02 | 72.88 | 75.17 | 21,720 | 257,254 | 73.23 | 72.12 | 74.36 | 24,557 | 274,437 | 76.56 | 75.45 | 77.69 |
| East of England | 6,443 | 78,825 | 71.46 | 69.52 | 73.46 | 6,487 | 77,408 | 73.52 | 71.51 | 75.60 | 6,737 | 78,484 | 73.70 | 71.66 | 75.78 |
| London | 28,050 | 492,283 | 51.58 | 50.93 | 52.24 | 30,915 | 528,678 | 51.90 | 51.27 | 52.55 | 34,032 | 560,363 | 53.55 | 52.91 | 54.19 |
| South East | 28,661 | 366,860 | 69.42 | 68.53 | 70.33 | 31,026 | 386,877 | 69.98 | 69.10 | 70.87 | 34,217 | 412,321 | 71.96 | 71.09 | 72.85 |
| South West | 17,675 | 209,417 | 76.18 | 74.94 | 77.43 | 19,199 | 218,191 | 77.29 | 76.05 | 78.55 | 21,070 | 223,378 | 83.21 | 81.92 | 84.52 |
| Deprivation † | | | | | | | | | | | | | | | |
| Fifth 1 (least deprived) | 20,638 | 278,683 | 65.76 | 64.77 | 66.76 | 22,600 | 297,632 | 66.39 | 65.41 | 67.38 | 25,291 | 317,500 | 69.01 | 68.04 | 70.00 |
| Fifth 2 | 27,059 | 388,425 | 62.14 | 61.32 | 62.96 | 29,388 | 411,493 | 62.37 | 61.57 | 63.18 | 32,545 | 435,448 | 64.56 | 63.76 | 65.37 |
| Fifth 3 | 29,477 | 401,986 | 64.62 | 63.81 | 65.45 | 31,807 | 424,334 | 64.74 | 63.93 | 65.56 | 34,435 | 442,198 | 66.43 | 65.62 | 67.25 |
| Fifth 4 | 42,050 | 573,815 | 64.93 | 64.25 | 65.63 | 45,676 | 600,982 | 65.59 | 64.90 | 66.28 | 49,947 | 631,674 | 67.97 | 67.28 | 68.66 |
| Fifth 5 (most deprived) | 44,704 | 542,711 | 72.73 | 71.97 | 73.49 | 48,076 | 565,297 | 73.12 | 72.36 | 73.88 | 53,538 | 593,098 | 77.01 | 76.24 | 77.78 |

PY: person-years; IR: incidence rate (per 1,000 person-years); 95% CI: 95% confidence intervals

† England only

**Supplement C table 2** **(pg 3) Primary care-recorded CMD, incidence (per 1,000 person-years) and 95%CI, 2009-2019, CPRD**

| Variable | 2015 | | | | | 2016 | | | | | 2017 | | | | |
|---|---|---|---|---|---|---|---|---|---|---|---|---|---|---|---|
| | Cases | PY | IR | 95% CI | | Cases | PY | IR | 95% CI | | Cases | PY | IR | 95% CI | |
| Total | | | | | | | | | | | | | | | |
| | 216,065 | 2,549,888 | 72.49 | 72.14 | 72.85 | 226,714 | 2,703,312 | 71.50 | 71.16 | 71.85 | 240,729 | 2,885,606 | 70.35 | 70.02 | 70.69 |
| Sex | | | | | | | | | | | | | | | |
| Female | 138,858 | 1,302,846 | 92.55 | 91.97 | 93.13 | 145,361 | 1,378,628 | 91.57 | 91.00 | 92.14 | 152,930 | 1,467,573 | 89.53 | 88.98 | 90.08 |
| Male | 77,207 | 1,247,042 | 53.22 | 52.79 | 53.65 | 81,353 | 1,324,684 | 52.41 | 52.00 | 52.83 | 87,799 | 1,418,033 | 52.34 | 51.93 | 52.74 |
| Age group | | | | | | | | | | | | | | | |
| 16-19 | 12,158 | 97,302 | 122.14 | 119.94 | 124.38 | 12,614 | 97,390 | 126.54 | 124.30 | 128.82 | 12,483 | 97,235 | 124.93 | 122.70 | 127.20 |
| 20-24 | 46,758 | 505,570 | 84.80 | 83.97 | 85.64 | 46,862 | 504,090 | 85.19 | 84.35 | 86.04 | 46,915 | 508,741 | 84.76 | 83.92 | 85.61 |
| 25-29 | 63,026 | 710,930 | 75.94 | 75.26 | 76.64 | 61,669 | 707,749 | 74.90 | 74.21 | 75.60 | 62,423 | 711,275 | 74.75 | 74.06 | 75.45 |
| 30-34 | 85,163 | 1,020,463 | 65.66 | 65.11 | 66.21 | 80,225 | 966,799 | 65.16 | 64.59 | 65.73 | 76,747 | 927,786 | 64.79 | 64.21 | 65.37 |
| 35-39 | 8,960 | 215,623 | 31.91 | 31.08 | 32.77 | 25,344 | 427,284 | 45.10 | 44.38 | 45.83 | 42,161 | 640,569 | 48.81 | 48.19 | 49.44 |
| Cohort | | | | | | | | | | | | | | | |
| 1980-84 | 87,171 | 1,077,886 | 63.51 | 62.98 | 64.05 | 85,322 | 1,071,350 | 61.48 | 60.95 | 62.01 | 84,717 | 1,072,154 | 59.54 | 59.01 | 60.07 |
| 1985-89 | 64,360 | 745,864 | 73.78 | 73.11 | 74.45 | 64,903 | 775,685 | 70.97 | 70.32 | 71.62 | 66,936 | 808,635 | 68.70 | 68.07 | 69.34 |
| 1990-94 | 48,743 | 553,545 | 80.68 | 79.90 | 81.47 | 51,526 | 590,865 | 78.83 | 78.07 | 79.59 | 54,604 | 634,674 | 76.55 | 75.82 | 77.28 |
| 1995-99 | 15,791 | 172,594 | 89.36 | 87.94 | 90.80 | 24,644 | 261,369 | 90.95 | 89.78 | 92.14 | 33,228 | 353,383 | 89.78 | 88.76 | 90.80 |
| 2000-03 | - | - | - | - | - | 319 | 4,042 | 78.91 | 70.71 | 88.07 | 1,244 | 16,760 | 73.82 | 69.81 | 78.07 |
| Ethnicity | | | | | | | | | | | | | | | |
| Asian | 13,140 | 285,062 | 38.83 | 38.08 | 39.59 | 14,317 | 304,302 | 39.41 | 38.67 | 40.16 | 16,054 | 328,619 | 40.48 | 39.76 | 41.21 |
| Black | 6,651 | 101,995 | 56.44 | 54.91 | 58.02 | 6,901 | 109,890 | 53.31 | 51.86 | 54.79 | 7,636 | 117,858 | 54.50 | 53.08 | 55.96 |
| Mixed | 6,320 | 75,365 | 75.43 | 73.36 | 77.56 | 6,737 | 83,606 | 71.80 | 69.86 | 73.78 | 7,627 | 93,609 | 72.43 | 70.58 | 74.32 |
| Other | 2,255 | 39,850 | 51.29 | 49.01 | 53.68 | 2,551 | 44,445 | 50.91 | 48.75 | 53.17 | 2,874 | 50,065 | 51.07 | 49.01 | 53.20 |
| White | 135,437 | 1,298,763 | 91.87 | 91.30 | 92.45 | 141,712 | 1,390,620 | 89.60 | 89.05 | 90.16 | 150,188 | 1,492,748 | 87.94 | 87.41 | 88.48 |
| Not stated | 52,262 | 748,853 | 57.65 | 57.07 | 58.24 | 54,496 | 770,449 | 58.26 | 57.68 | 58.84 | 56,350 | 802,707 | 56.26 | 55.70 | 56.83 |
| Country | | | | | | | | | | | | | | | |
| England | 186,522 | 2,195,209 | 73.58 | 73.19 | 73.97 | 195,967 | 2,331,269 | 72.65 | 72.28 | 73.03 | 208,280 | 2,495,952 | 71.33 | 70.97 | 71.70 |
| Scotland | 15,299 | 199,401 | 61.00 | 59.84 | 62.19 | 16,069 | 210,674 | 58.55 | 57.43 | 59.69 | 16,665 | 219,416 | 57.97 | 56.87 | 59.09 |
| Northern Ireland | 3,297 | 36,225 | 66.56 | 63.62 | 69.64 | 3,368 | 37,188 | 68.22 | 65.24 | 71.34 | 3,550 | 39,213 | 66.84 | 63.93 | 69.88 |
| Wales | 10,947 | 119,053 | 73.37 | 71.70 | 75.08 | 11,310 | 124,181 | 72.84 | 71.19 | 74.53 | 12,234 | 131,024 | 73.57 | 71.94 | 75.24 |
| Region † | | | | | | | | | | | | | | | |
| North East | 6,246 | 61,250 | 89.22 | 86.63 | 91.88 | 6,667 | 64,913 | 88.25 | 85.72 | 90.86 | 6,690 | 66,951 | 85.60 | 83.12 | 88.16 |
| North West | 33,504 | 341,833 | 84.90 | 83.83 | 85.99 | 35,644 | 366,110 | 83.91 | 82.87 | 84.97 | 38,173 | 391,867 | 82.97 | 81.96 | 84.00 |
| Yorksh. & Humb. | 7,669 | 88,119 | 75.63 | 73.70 | 77.61 | 8,297 | 91,797 | 78.42 | 76.47 | 80.42 | 8,885 | 96,617 | 80.70 | 78.75 | 82.70 |
| East Midlands | 5,378 | 73,307 | 64.63 | 62.71 | 66.61 | 5,939 | 76,625 | 69.77 | 67.80 | 71.79 | 6,682 | 80,647 | 74.01 | 72.01 | 76.06 |
| West Midlands | 27,484 | 295,237 | 79.98 | 78.87 | 81.11 | 28,714 | 316,148 | 78.36 | 77.29 | 79.45 | 30,724 | 340,236 | 76.33 | 75.30 | 77.37 |
| East of England | 6,973 | 76,795 | 77.94 | 75.80 | 80.13 | 6,755 | 75,648 | 77.69 | 75.53 | 79.91 | 7,066 | 80,312 | 74.50 | 72.42 | 76.63 |
| London | 38,601 | 596,357 | 57.45 | 56.81 | 58.10 | 40,850 | 645,166 | 55.40 | 54.79 | 56.02 | 44,064 | 700,385 | 54.57 | 53.99 | 55.16 |
| South East | 37,844 | 433,736 | 75.42 | 74.54 | 76.31 | 38,904 | 453,453 | 74.16 | 73.30 | 75.03 | 40,335 | 482,758 | 71.30 | 70.48 | 72.14 |
| South West | 22,823 | 228,576 | 87.04 | 85.73 | 88.38 | 24,197 | 241,410 | 88.53 | 87.22 | 89.85 | 25,661 | 256,180 | 88.17 | 86.90 | 89.47 |
| Deprivation † | | | | | | | | | | | | | | | |
| Fifth 1 (least deprived) | 28,594 | 339,436 | 72.69 | 71.72 | 73.68 | 30,817 | 364,171 | 73.59 | 72.63 | 74.55 | 32,641 | 392,370 | 70.85 | 69.95 | 71.78 |
| Fifth 2 | 35,915 | 461,416 | 67.12 | 66.32 | 67.93 | 38,104 | 487,418 | 67.40 | 66.61 | 68.19 | 40,905 | 520,182 | 67.18 | 66.41 | 67.95 |
| Fifth 3 | 37,451 | 464,224 | 68.46 | 67.65 | 69.28 | 39,886 | 493,593 | 68.45 | 67.66 | 69.25 | 42,326 | 523,869 | 67.32 | 66.55 | 68.10 |
| Fifth 4 | 55,713 | 661,404 | 72.45 | 71.75 | 73.15 | 57,868 | 698,850 | 70.53 | 69.86 | 71.22 | 62,007 | 747,249 | 70.49 | 69.83 | 71.16 |
| Fifth 5 (most deprived) | 58,392 | 623,408 | 79.70 | 78.94 | 80.48 | 60,039 | 659,279 | 76.94 | 76.19 | 77.68 | 62,850 | 747,249 | 74.73 | 74.02 | 75.45 |

PY: person-years; IR: incidence rate (per 1,000 person-years); 95% CI: 95% confidence intervals

† England only

**Supplement C table 2** **(pg 4) Incidence rate of primary care-recorded CMD (per 1,000 person-years), 2009-**

| Variable | 2018 | | | | | 2019 | | | | |
|---|---|---|---|---|---|---|---|---|---|---|
| | Cases | PY | IR | 95% CI | | Cases | PY | IR | 95% CI | |
| Total | | | | | | | | | | |
| | 260,757 | 3,058,949 | 71.70 | 71.37 | 72.04 | 281,258 | 3,183,874 | 74.79 | 74.45 | 75.13 |
| Sex | | | | | | | | | | |
| Female | 164,219 | 1,548,472 | 91.07 | 90.53 | 91.61 | 177,253 | 1,601,960 | 96.23 | 95.68 | 96.78 |
| Male | 96,538 | 1,510,477 | 53.80 | 53.40 | 54.20 | 104,005 | 1,581,914 | 55.31 | 54.91 | 55.71 |
| Age group | | | | | | | | | | |
| 16-19 | 13,056 | 95,736 | 133.27 | 130.95 | 135.63 | 13,499 | 93,571 | 140.74 | 138.33 | 143.20 |
| 20-24 | 48,905 | 513,887 | 87.09 | 86.24 | 87.94 | 51,686 | 510,600 | 93.52 | 92.63 | 94.40 |
| 25-29 | 63,625 | 707,170 | 76.72 | 76.02 | 77.43 | 65,109 | 694,700 | 80.79 | 80.06 | 81.52 |
| 30-34 | 75,421 | 891,331 | 66.58 | 65.98 | 67.19 | 74,095 | 839,986 | 70.63 | 69.99 | 71.27 |
| 35-39 | 59,750 | 850,826 | 52.12 | 51.56 | 52.69 | 76,869 | 1,045,018 | 54.45 | 53.93 | 54.98 |
| Cohort | | | | | | | | | | |
| 1980-84 | 85,535 | 1,069,571 | 59.57 | 59.04 | 60.11 | 85,808 | 1,049,482 | 60.73 | 60.17 | 61.28 |
| 1985-89 | 69,926 | 832,508 | 69.06 | 68.43 | 69.70 | 72,250 | 839,524 | 70.39 | 69.75 | 71.03 |
| 1990-94 | 58,922 | 673,559 | 76.48 | 75.77 | 77.20 | 63,633 | 695,034 | 80.00 | 79.27 | 80.73 |
| 1995-99 | 42,481 | 442,065 | 90.81 | 89.88 | 91.74 | 50,255 | 507,793 | 93.02 | 92.14 | 93.91 |
| 2000-03 | 3,893 | 41,247 | 93.16 | 90.24 | 96.18 | 9,312 | 92,041 | 99.31 | 97.27 | 101.39 |
| Ethnicity | | | | | | | | | | |
| Asian | 18,559 | 355,049 | 43.65 | 42.92 | 44.38 | 20,251 | 378,113 | 44.57 | 43.86 | 45.29 |
| Black | 8,610 | 125,373 | 58.00 | 56.57 | 59.46 | 9,734 | 131,364 | 62.19 | 60.74 | 63.68 |
| Mixed | 8,640 | 103,083 | 73.83 | 72.05 | 75.66 | 9,707 | 110,407 | 78.25 | 76.46 | 80.08 |
| Other | 3,218 | 56,048 | 49.85 | 47.92 | 51.85 | 3,650 | 61,864 | 52.42 | 50.53 | 54.37 |
| White | 161,496 | 1,576,943 | 89.46 | 88.93 | 89.99 | 172,983 | 1,623,478 | 93.97 | 93.43 | 94.51 |
| Not stated | 60,234 | 842,453 | 56.87 | 56.31 | 57.43 | 64,933 | 878,648 | 59.27 | 58.71 | 59.84 |
| Country | | | | | | | | | | |
| England | 226,260 | 2,648,713 | 72.80 | 72.44 | 73.16 | 244,592 | 2,753,525 | 76.27 | 75.91 | 76.64 |
| Scotland | 17,977 | 231,718 | 59.76 | 58.67 | 60.87 | 19,010 | 244,215 | 59.44 | 58.37 | 60.52 |
| Northern Ireland | 3,763 | 41,416 | 64.26 | 61.46 | 67.19 | 3,872 | 43,668 | 64.19 | 61.44 | 67.06 |
| Wales | 12,757 | 137,102 | 73.06 | 71.46 | 74.70 | 13,784 | 142,467 | 75.65 | 74.03 | 77.30 |
| Region † | | | | | | | | | | |
| North East | 7,360 | 69,579 | 91.15 | 88.61 | 93.76 | 7,958 | 72,323 | 96.92 | 94.34 | 99.57 |
| North West | 41,899 | 416,160 | 84.63 | 83.62 | 85.64 | 46,407 | 436,862 | 91.57 | 90.54 | 92.61 |
| Yorksh. & Humb. | 9,455 | 101,542 | 80.56 | 78.64 | 82.52 | 9,404 | 101,038 | 80.12 | 78.19 | 82.10 |
| East Midlands | 7,216 | 87,463 | 73.87 | 71.94 | 75.86 | 7,384 | 88,541 | 73.12 | 71.21 | 75.08 |
| West Midlands | 33,892 | 357,913 | 80.56 | 79.52 | 81.61 | 36,809 | 364,141 | 86.88 | 85.80 | 87.98 |
| East of England | 7,238 | 83,277 | 74.48 | 72.42 | 76.60 | 7,492 | 83,147 | 78.52 | 76.39 | 80.70 |
| London | 47,394 | 751,393 | 54.32 | 53.76 | 54.89 | 51,397 | 795,168 | 55.49 | 54.94 | 56.05 |
| South East | 44,242 | 513,186 | 73.92 | 73.10 | 74.75 | 47,738 | 533,734 | 77.76 | 76.92 | 78.60 |
| South West | 27,564 | 268,200 | 90.55 | 89.27 | 91.84 | 30,003 | 278,572 | 95.64 | 94.35 | 96.95 |
| Deprivation † | | | | | | | | | | |
| Fifth 1 (least deprived) | 35,715 | 418,652 | 73.19 | 72.29 | 74.11 | 38,497 | 436,050 | 76.30 | 75.39 | 77.22 |
| Fifth 2 | 44,536 | 551,688 | 68.72 | 67.97 | 69.49 | 48,463 | 576,555 | 71.68 | 70.92 | 72.45 |
| Fifth 3 | 45,516 | 552,548 | 68.67 | 67.91 | 69.44 | 49,106 | 574,852 | 71.91 | 71.14 | 72.69 |
| Fifth 4 | 66,613 | 796,571 | 70.57 | 69.93 | 71.22 | 71,844 | 828,953 | 73.35 | 72.70 | 74.00 |
| Fifth 5 (most deprived) | 68,377 | 739,490 | 76.80 | 76.09 | 77.51 | 73,348 | 767,464 | 80.22 | 79.50 | 80.94 |

PY: person-years; IR: incidence rate (per 1,000 person-years); 95% CI: 95% confidence intervals

† England only

**Supplement C table 3** **Primary care-recorded CMD over time, incidence rate ratios from 2009 to 2014; 2014 to 2019, and 2009 to 2019, CPRD**

| | 2014 : 2009 | | | 2019 : 2014 | | | 2019 : 2009 | | | Interaction‡ chi-square (df*); |
|---|---|---|---|---|---|---|---|---|---|---|
| | IRR | 95% CI | | IRR | 95% CI | | IRR | 95% CI | | p-value |
| Total | | | | | | | | | | |
| Overall | 1.02 | 1.01 | 1.03 | 1.08 | 1.07 | 1.09 | 1.10 | 1.09 | 1.11 | |
| Sex | | | | | | | | | | 1,687 (1); |
| Female | 1.00 | 0.99 | 1.01 | 1.08 | 1.07 | 1.09 | 1.08 | 1.07 | 1.09 | p>0.001 |
| Male | 1.09 | 1.08 | 1.11 | 1.10 | 1.09 | 1.11 | 1.21 | 1.19 | 1.22 | |
| Age group | | | | | | | | | | 754 (4); |
| 16-19 | 1.25 | 1.21 | 1.28 | 1.31 | 1.28 | 1.34 | 1.63 | 1.59 | 1.68 | p>0.001 |
| 20-24 | 1.02 | 1.01 | 1.04 | 1.20 | 1.18 | 1.21 | 1.22 | 1.21 | 1.24 | |
| 25-29 | 1.18 | 1.17 | 1.20 | 1.09 | 1.08 | 1.11 | 1.30 | 1.28 | 1.31 | |
| 30-34˜ | 1.03 | 1.02 | 1.05 | 1.23 | 1.21 | 1.24 | 1.27 | 1.25 | 1.28 ˜ | |
| 35-39° | - | - | - | 1.71 | 1.66 | 1.76 | 1.71 | 1.66 | 1.76 ° | |
| Cohort | | | | | | | | | | 794 (4); |
| 1980-84 | 0.93 | 0.92 | 0.94 | 0.95 | 0.94 | 0.97 | 0.89 | 0.87 | 0.90 | p>0.001 |
| 1985-89 | 1.05 | 1.04 | 1.06 | 0.99 | 0.97 | 1.00 | 1.03 | 1.02 | 1.05 | |
| 1990-94 | 1.22 | 1.19 | 1.26 | 1.06 | 1.04 | 1.07 | 1.29 | 1.26 | 1.33 | |
| 1995-99^ | 1.77 | 1.53 | 2.06 | 1.18 | 1.15 | 1.21 | 2.09 | 1.81 | 2.44 ^ | |
| 2000-03± | - | - | - | 1.26 | 1.13 | 1.41 | 1.26 | 1.13 | 1.41 ± | |
| Ethnicity | | | | | | | | | | 3,753 (5); |
| Asian | 0.89 | 0.86 | 0.92 | 1.22 | 1.19 | 1.25 | 1.08 | 1.05 | 1.11 | p>0.001 |
| Black | 0.97 | 0.93 | 1.01 | 1.17 | 1.12 | 1.21 | 1.13 | 1.09 | 1.18 | |
| Mixed | 0.96 | 0.92 | 1.01 | 1.17 | 1.13 | 1.22 | 1.13 | 1.08 | 1.18 | |
| Other | 0.98 | 0.90 | 1.06 | 1.10 | 1.03 | 1.17 | 1.08 | 1.00 | 1.16 | |
| White | 0.94 | 0.93 | 0.95 | 1.06 | 1.05 | 1.07 | 1.00 | 0.99 | 1.01 | |
| Not stated | 1.23 | 1.21 | 1.25 | 1.07 | 1.05 | 1.08 | 1.31 | 1.29 | 1.33 | |
| Country | | | | | | | | | | 443 (3); |
| England | 1.03 | 1.02 | 1.04 | 1.09 | 1.08 | 1.10 | 1.12 | 1.11 | 1.13 | p>0.001 |
| Scotland | 0.93 | 0.90 | 0.96 | 0.98 | 0.95 | 1.01 | 0.91 | 0.89 | 0.94 | |
| Northern Ireland | 0.86 | 0.81 | 0.92 | 0.96 | 0.90 | 1.02 | 0.82 | 0.77 | 0.88 | |
| Wales | 0.99 | 0.95 | 1.02 | 1.07 | 1.03 | 1.10 | 1.05 | 1.02 | 1.09 | |
| Region Ϯ | | | | | | | | | | 1,280 (11); |
| North East | 1.00 | 0.95 | 1.04 | 1.10 | 1.06 | 1.15 | 1.10 | 1.05 | 1.14 | p>0.001 |
| North West | 1.06 | 1.04 | 1.08 | 1.11 | 1.09 | 1.13 | 1.18 | 1.16 | 1.20 | |
| Yorksh. & Humb. | 1.10 | 1.05 | 1.14 | 1.16 | 1.12 | 1.20 | 1.27 | 1.22 | 1.32 | |
| East Midlands | 1.06 | 1.01 | 1.11 | 1.23 | 1.18 | 1.29 | 1.31 | 1.25 | 1.36 | |
| West Midlands | 1.07 | 1.04 | 1.09 | 1.13 | 1.11 | 1.16 | 1.21 | 1.19 | 1.24 | |
| East of England | 0.96 | 0.92 | 0.99 | 1.07 | 1.02 | 1.11 | 1.02 | 0.98 | 1.06 | |
| London | 1.01 | 0.99 | 1.03 | 1.04 | 1.02 | 1.05 | 1.04 | 1.02 | 1.06 | |
| South East | 1.04 | 1.02 | 1.06 | 1.08 | 1.06 | 1.10 | 1.13 | 1.11 | 1.15 | |
| South West | 1.12 | 1.09 | 1.15 | 1.15 | 1.13 | 1.17 | 1.29 | 1.26 | 1.32 | |
| Deprivation Ϯ | | | | | | | | | | 220 (4); |
| Fifth 1 (least deprived) | 1.05 | 1.03 | 1.08 | 1.11 | 1.09 | 1.13 | 1.16 | 1.14 | 1.19 | p>0.001 |
| Fifth 2 | 1.04 | 1.02 | 1.06 | 1.11 | 1.09 | 1.13 | 1.16 | 1.14 | 1.18 | |
| Fifth 3 | 1.01 | 0.99 | 1.03 | 1.08 | 1.06 | 1.10 | 1.10 | 1.08 | 1.11 | |
| Fifth 4 | 1.03 | 1.01 | 1.04 | 1.08 | 1.06 | 1.09 | 1.11 | 1.09 | 1.12 | |
| Fifth 5 (most deprived) | 0.99 | 0.98 | 1.01 | 1.04 | 1.03 | 1.06 | 1.04 | 1.02 | 1.05 | |

Ϯ England only

˜Used earliest estimate (2013); °Used earliest estimate (2015); ^ Used earliest estimate (2011); ±Used earliest estimate (2016)

‡ Wald test for interaction

* df=degrees of freedom

**Supplement C table 3** **Incidence rate ratios of primary care-recorded CMD by sociodemographic group, 2009-2019, CPRD**

| Variable | 2009 | | | 2010 | | | 2011 | | | 2012 | | |
|---|---|---|---|---|---|---|---|---|---|---|---|---|
| | IRR | 95% CI | | IRR | 95% CI | | IRR | 95% CI | | IRR | 95% CI | |
| Sex | | | | | | | | | | | | |
| Female | 1.95 | 1.93 | 1.97 | 1.97 | 1.95 | 1.99 | 1.93 | 1.90 | 1.95 | 1.84 | 1.82 | 1.86 |
| Male (ref.) | 1.00 | | | 1.00 | | | 1.00 | | | 1.00 | | |
| Age group | | | | | | | | | | | | |
| 16-19 | 1.38 | 1.35 | 1.41 | 1.29 | 1.26 | 1.32 | 1.27 | 1.24 | 1.30 | 1.35 | 1.32 | 1.38 |
| 20-24 | 1.23 | 1.21 | 1.24 | 1.01 | 1.00 | 1.02 | 1.02 | 1.01 | 1.03 | 1.04 | 1.02 | 1.05 |
| 25-29 (ref.) | 1.00 | | | 1.00 | | | 1.00 | | | 1.00 | | |
| 30-34 | - | - | - | 0.47 | 0.46 | 0.48 | 0.71 | 0.70 | 0.72 | 0.78 | 0.76 | 0.79 |
| 35-39 | - | - | - | - | - | - | - | - | - | - | - | - |
| Cohort | | | | | | | | | | | | |
| 1980-84 (ref.) | 1.00 | | | 1.00 | | | 1.00 | | | 1.00 | | |
| 1985-89 | 0.99 | 0.98 | 1.00 | 1.02 | 1.01 | 1.04 | 1.05 | 1.04 | 1.07 | 1.07 | 1.05 | 1.08 |
| 1990-94 | 0.90 | 0.88 | 0.93 | 0.97 | 0.95 | 0.99 | 1.01 | 0.99 | 1.03 | 1.10 | 1.08 | 1.11 |
| 1995-99 | - | - | - | - | - | - | 0.67 | 0.57 | 0.77 | 0.89 | 0.83 | 0.95 |
| 2000-03 | - | - | - | - | - | - | - | - | - | - | - | - |
| Ethnicity | | | | | | | | | | | | |
| Asian | 0.48 | 0.47 | 0.49 | 0.46 | 0.45 | 0.47 | 0.45 | 0.44 | 0.46 | 0.45 | 0.44 | 0.46 |
| Black | 0.71 | 0.69 | 0.73 | 0.73 | 0.71 | 0.75 | 0.71 | 0.69 | 0.73 | 0.75 | 0.73 | 0.76 |
| Mixed | 0.73 | 0.70 | 0.75 | 0.75 | 0.72 | 0.77 | 0.73 | 0.71 | 0.75 | 0.78 | 0.76 | 0.80 |
| Other | 0.57 | 0.53 | 0.60 | 0.58 | 0.54 | 0.61 | 0.58 | 0.55 | 0.62 | 0.62 | 0.58 | 0.65 |
| White (ref.) | 1.00 | | | 1.00 | | | 1.00 | | | 1.00 | | |
| Not stated | 0.48 | 0.47 | 0.49 | 0.48 | 0.48 | 0.49 | 0.49 | 0.48 | 0.49 | 0.52 | 0.51 | 0.52 |
| Country | | | | | | | | | | | | |
| England (ref.) | 1.00 | | | 1.00 | | | 1.00 | | | 1.00 | | |
| Scotland | 0.96 | 0.94 | 0.98 | 0.99 | 0.97 | 1.01 | 0.95 | 0.93 | 0.97 | 0.92 | 0.90 | 0.94 |
| Northern Ireland | 1.15 | 1.10 | 1.20 | 1.22 | 1.17 | 1.27 | 1.10 | 1.05 | 1.15 | 1.06 | 1.02 | 1.11 |
| Wales | 1.06 | 1.03 | 1.09 | 1.03 | 1.00 | 1.06 | 1.03 | 1.01 | 1.06 | 1.01 | 0.98 | 1.03 |
| Region Ϯ | | | | | | | | | | | | |
| North East | 1.66 | 1.60 | 1.72 | 1.63 | 1.57 | 1.68 | 1.49 | 1.44 | 1.54 | 1.53 | 1.48 | 1.58 |
| North West | 1.46 | 1.43 | 1.49 | 1.51 | 1.48 | 1.54 | 1.50 | 1.47 | 1.53 | 1.48 | 1.46 | 1.51 |
| Yorkshire & Humb. | 1.18 | 1.14 | 1.22 | 1.23 | 1.19 | 1.27 | 1.30 | 1.26 | 1.34 | 1.28 | 1.24 | 1.32 |
| East Midlands | 1.05 | 1.02 | 1.09 | 1.07 | 1.03 | 1.10 | 1.11 | 1.07 | 1.15 | 1.06 | 1.03 | 1.10 |
| West Midlands | 1.35 | 1.32 | 1.38 | 1.40 | 1.37 | 1.43 | 1.45 | 1.42 | 1.48 | 1.44 | 1.41 | 1.46 |
| East of England | 1.45 | 1.40 | 1.49 | 1.47 | 1.42 | 1.51 | 1.41 | 1.36 | 1.45 | 1.39 | 1.34 | 1.43 |
| London (ref.) | 1.00 | | | 1.00 | | | 1.00 | | | 1.00 | | |
| South East | 1.30 | 1.27 | 1.32 | 1.32 | 1.29 | 1.35 | 1.36 | 1.33 | 1.38 | 1.35 | 1.32 | 1.37 |
| South West | 1.40 | 1.36 | 1.43 | 1.44 | 1.41 | 1.47 | 1.45 | 1.42 | 1.49 | 1.48 | 1.45 | 1.51 |
| Deprivation Ϯ | | | | | | | | | | | | |
| Fifth 1 (least deprived, ref.) | 1.00 | | | 1.00 | | | 1.00 | | | 1.00 | | |
| Fifth 2 | 0.94 | 0.92 | 0.96 | 0.95 | 0.93 | 0.97 | 0.93 | 0.91 | 0.95 | 0.94 | 0.93 | 0.96 |
| Fifth 3 | 1.00 | 0.98 | 1.02 | 1.02 | 1.00 | 1.04 | 0.99 | 0.97 | 1.01 | 0.98 | 0.96 | 1.00 |
| Fifth 4 | 1.01 | 0.99 | 1.03 | 1.02 | 1.00 | 1.04 | 0.99 | 0.97 | 1.01 | 0.99 | 0.97 | 1.01 |
| Fifth 5 (most deprived) | 1.18 | 1.16 | 1.20 | 1.18 | 1.16 | 1.20 | 1.14 | 1.12 | 1.17 | 1.11 | 1.09 | 1.13 |

IRR: incidence rate ratios; 95% CI: 95% confidence intervals

Ϯ England only

## Supplement C table 3 (pg 2) Incidence rate ratios of primary care-recorded CMD by sociodemographic group, 2009-2019, CPRD

| Variable | 2013 | | | 2014 | | | 2015 | | | 2016 | | |
|---|---|---|---|---|---|---|---|---|---|---|---|---|
| | IRR | 95% CI | | IRR | 95% CI | | IRR | 95% CI | | IRR | 95% CI | |
| Sex | | | | | | | | | | | | |
| Female | 1.81 | 1.79 | 1.83 | 1.78 | 1.76 | 1.79 | 1.74 | 1.72 | 1.76 | 1.75 | 1.73 | 1.76 |
| Male (ref.) | 1.00 | | | 1.00 | | | 1.00 | | | 1.00 | | |
| Age group | | | | | | | | | | | | |
| 16-19 | 1.39 | 1.36 | 1.42 | 1.46 | 1.42 | 1.49 | 1.61 | 1.58 | 1.64 | 1.69 | 1.66 | 1.72 |
| 20-24 | 1.06 | 1.04 | 1.07 | 1.06 | 1.04 | 1.07 | 1.12 | 1.10 | 1.13 | 1.14 | 1.12 | 1.15 |
| 25-29 (ref.) | 1.00 | | | 1.00 | | | 1.00 | | | 1.00 | | |
| 30-34 | 0.80 | 0.79 | 0.81 | 0.78 | 0.77 | 0.79 | 0.86 | 0.85 | 0.88 | 0.87 | 0.86 | 0.88 |
| 35-39 | - | - | - | - | - | - | 0.42 | 0.41 | 0.43 | 0.60 | 0.59 | 0.61 |
| Cohort | | | | | | | | | | | | |
| 1980-84 (ref.) | 1.00 | | | 1.00 | | | 1.00 | | | 1.00 | | |
| 1985-89 | 1.09 | 1.07 | 1.10 | 1.12 | 1.11 | 1.14 | 1.16 | 1.15 | 1.18 | 1.15 | 1.14 | 1.17 |
| 1990-94 | 1.13 | 1.12 | 1.15 | 1.19 | 1.18 | 1.21 | 1.27 | 1.25 | 1.29 | 1.28 | 1.27 | 1.30 |
| 1995-99 | 1.07 | 1.03 | 1.11 | 1.24 | 1.21 | 1.27 | 1.41 | 1.38 | 1.43 | 1.48 | 1.46 | 1.50 |
| 2000-03 | - | - | - | - | - | - | - | - | - | 1.28 | 1.16 | 1.42 |
| Ethnicity | | | | | | | | | | | | |
| Asian | 0.45 | 0.44 | 0.46 | 0.46 | 0.45 | 0.47 | 0.47 | 0.46 | 0.48 | 0.48 | 0.47 | 0.49 |
| Black | 0.74 | 0.72 | 0.76 | 0.75 | 0.74 | 0.77 | 0.79 | 0.78 | 0.81 | 0.77 | 0.75 | 0.78 |
| Mixed | 0.78 | 0.76 | 0.80 | 0.77 | 0.75 | 0.79 | 0.82 | 0.80 | 0.84 | 0.81 | 0.79 | 0.83 |
| Other | 0.64 | 0.61 | 0.68 | 0.61 | 0.58 | 0.64 | 0.63 | 0.60 | 0.66 | 0.64 | 0.61 | 0.67 |
| White (ref.) | 1.00 | | | 1.00 | | | 1.00 | | | 1.00 | | |
| Not stated | 0.56 | 0.56 | 0.57 | 0.63 | 0.62 | 0.64 | 0.63 | 0.62 | 0.64 | 0.65 | 0.64 | 0.66 |
| Country | | | | | | | | | | | | |
| England (ref.) | 1.00 | | | 1.00 | | | 1.00 | | | 1.00 | | |
| Scotland | 0.89 | 0.87 | 0.91 | 0.87 | 0.85 | 0.88 | 0.83 | 0.81 | 0.85 | 0.81 | 0.79 | 0.82 |
| Northern Ireland | 0.98 | 0.93 | 1.02 | 0.96 | 0.91 | 1.00 | 0.90 | 0.86 | 0.95 | 0.94 | 0.90 | 0.98 |
| Wales | 1.02 | 0.99 | 1.04 | 1.01 | 0.99 | 1.04 | 1.00 | 0.97 | 1.02 | 1.00 | 0.98 | 1.03 |
| Region † | | | | | | | | | | | | |
| North East | 1.50 | 1.45 | 1.55 | 1.64 | 1.59 | 1.70 | 1.55 | 1.50 | 1.60 | 1.59 | 1.54 | 1.64 |
| North West | 1.52 | 1.49 | 1.55 | 1.54 | 1.52 | 1.57 | 1.48 | 1.45 | 1.50 | 1.51 | 1.49 | 1.54 |
| Yorkshire & Humb. | 1.30 | 1.26 | 1.34 | 1.29 | 1.25 | 1.33 | 1.32 | 1.28 | 1.35 | 1.42 | 1.38 | 1.45 |
| East Midlands | 1.07 | 1.04 | 1.11 | 1.11 | 1.07 | 1.14 | 1.13 | 1.09 | 1.16 | 1.26 | 1.22 | 1.30 |
| West Midlands | 1.41 | 1.38 | 1.44 | 1.43 | 1.40 | 1.46 | 1.39 | 1.37 | 1.42 | 1.41 | 1.39 | 1.44 |
| East of England | 1.42 | 1.37 | 1.46 | 1.38 | 1.33 | 1.42 | 1.36 | 1.32 | 1.40 | 1.40 | 1.36 | 1.45 |
| London (ref.) | 1.00 | | | 1.00 | | | 1.00 | | | 1.00 | | |
| South East | 1.35 | 1.32 | 1.37 | 1.34 | 1.32 | 1.37 | 1.31 | 1.29 | 1.33 | 1.34 | 1.32 | 1.36 |
| South West | 1.49 | 1.46 | 1.52 | 1.55 | 1.52 | 1.58 | 1.52 | 1.49 | 1.54 | 1.60 | 1.57 | 1.63 |
| Deprivation † | | | | | | | | | | | | |
| Fifth 1 (least deprived, ref.) | 1.00 | | | 1.00 | | | 1.00 | | | 1.00 | | |
| Fifth 2 | 0.94 | 0.92 | 0.96 | 0.94 | 0.92 | 0.95 | 0.92 | 0.91 | 0.94 | 0.92 | 0.90 | 0.93 |
| Fifth 3 | 0.98 | 0.96 | 0.99 | 0.96 | 0.94 | 0.98 | 0.94 | 0.92 | 0.96 | 0.93 | 0.91 | 0.95 |
| Fifth 4 | 0.99 | 0.97 | 1.01 | 0.98 | 0.97 | 1.00 | 1.00 | 0.98 | 1.01 | 0.96 | 0.94 | 0.97 |
| Fifth 5 (most deprived) | 1.10 | 1.08 | 1.12 | 1.12 | 1.10 | 1.14 | 1.10 | 1.08 | 1.11 | 1.05 | 1.03 | 1.06 |

IRR: incidence rate ratios; 95% CI: 95% confidence intervals

† England only

## Supplement C table 3 (pg 3) Incidence rate ratios of primary care-recorded CMD by sociodemographic group, 2009-2019, CPRD

| Variable | 2017 | | | 2018 | | | 2019 | | |
|---|---|---|---|---|---|---|---|---|---|
| | IRR | 95% CI | | IRR | 95% CI | | IRR | 95% CI | |
| Sex | | | | | | | | | |
| Female | 1.71 | 1.69 | 1.73 | 1.69 | 1.68 | 1.71 | 1.74 | 1.72 | 1.76 |
| Male (ref.) | 1.00 | | 1.75 | 1.00 | | | 1.00 | | |
| Age group | | | | | | | | | |
| 16-19 | 1.67 | 1.64 | 1.706 | 1.74 | 1.70 | 1.77 | 1.74 | 1.71 | 1.78 |
| 20-24 | 1.13 | 1.12 | 1.15 | 1.14 | 1.12 | 1.15 | 1.16 | 1.14 | 1.17 |
| 25-29 (ref.) | 1.00 | | | 1.00 | | | 1.00 | | |
| 30-34 | 0.87 | 0.86 | 0.88 | 0.87 | 0.86 | 0.88 | 0.87 | 0.86 | 0.89 |
| 35-39 | 0.65 | 0.64 | 0.66 | 0.68 | 0.67 | 0.69 | 0.67 | 0.67 | 0.68 |
| Cohort | | | | | | | | | |
| 1980-84 (ref.) | 1.00 | | | 1.00 | | | 1.00 | | |
| 1985-89 | 1.15 | 1.14 | 1.17 | 1.16 | 1.14 | 1.17 | 1.16 | 1.14 | 1.17 |
| 1990-94 | 1.29 | 1.27 | 1.30 | 1.28 | 1.27 | 1.30 | 1.32 | 1.30 | 1.33 |
| 1995-99 | 1.51 | 1.49 | 1.53 | 1.52 | 1.50 | 1.55 | 1.53 | 1.51 | 1.55 |
| 2000-03 | 1.24 | 1.18 | 1.30 | 1.56 | 1.53 | 1.60 | 1.64 | 1.62 | 1.65 |
| Ethnicity | | | | | | | | | |
| Asian | 0.50 | 0.49 | 0.51 | 0.53 | 0.52 | 0.54 | 0.52 | 0.51 | 0.53 |
| Black | 0.80 | 0.78 | 0.81 | 0.81 | 0.80 | 0.83 | 0.83 | 0.82 | 0.85 |
| Mixed | 0.83 | 0.81 | 0.84 | 0.81 | 0.80 | 0.83 | 0.82 | 0.81 | 0.84 |
| Other | 0.65 | 0.62 | 0.68 | 0.62 | 0.60 | 0.65 | 0.63 | 0.61 | 0.65 |
| White (ref.) | 1.00 | | | 1.00 | | | 1.00 | | |
| Not stated | 0.64 | 0.63 | 0.65 | 0.64 | 0.63 | 0.64 | 0.63 | 0.62 | 0.64 |
| Country | | | | | | | | | |
| England (ref.) | 1.00 | | | 1.00 | | | 1.00 | | |
| Scotland | 0.81 | 0.80 | 0.83 | 0.82 | 0.81 | 0.84 | 0.78 | 0.76 | 0.79 |
| Northern Ireland | 0.94 | 0.90 | 0.98 | 0.88 | 0.84 | 0.92 | 0.84 | 0.80 | 0.88 |
| Wales | 1.03 | 1.01 | 1.06 | 1.00 | 0.98 | 1.03 | 0.99 | 0.97 | 1.01 |
| Region † | | | | | | | | | |
| North East | 1.57 | 1.52 | 1.62 | 1.68 | 1.63 | 1.73 | 1.75 | 1.70 | 1.80 |
| North West | 1.52 | 1.50 | 1.55 | 1.56 | 1.53 | 1.58 | 1.65 | 1.63 | 1.68 |
| Yorkshire & Humb. | 1.48 | 1.44 | 1.52 | 1.48 | 1.44 | 1.52 | 1.44 | 1.41 | 1.48 |
| East Midlands | 1.36 | 1.32 | 1.40 | 1.36 | 1.32 | 1.40 | 1.32 | 1.28 | 1.36 |
| West Midlands | 1.40 | 1.37 | 1.42 | 1.48 | 1.46 | 1.51 | 1.57 | 1.54 | 1.59 |
| East of England | 1.37 | 1.32 | 1.41 | 1.37 | 1.33 | 1.41 | 1.41 | 1.37 | 1.46 |
| London (ref.) | 1.00 | | | 1.00 | | | 1.00 | | |
| South East | 1.31 | 1.29 | 1.33 | 1.36 | 1.34 | 1.38 | 1.40 | 1.38 | 1.42 |
| South West | 1.62 | 1.59 | 1.65 | 1.67 | 1.64 | 1.70 | 1.72 | 1.69 | 1.75 |
| Deprivation † | | | | | | | | | |
| Fifth 1 (least deprived, ref.) | 1.00 | | | 1.00 | | | 1.00 | | |
| Fifth 2 | 0.95 | 0.93 | 0.96 | 0.94 | 0.92 | 0.95 | 0.94 | 0.92 | 0.95 |
| Fifth 3 | 0.95 | 0.93 | 0.97 | 0.94 | 0.92 | 0.95 | 0.94 | 0.93 | 0.96 |
| Fifth 4 | 0.99 | 0.98 | 1.01 | 0.96 | 0.95 | 0.98 | 0.96 | 0.95 | 0.98 |
| Fifth 5 (most deprived) | 1.05 | 1.04 | 1.07 | 1.05 | 1.03 | 1.07 | 1.05 | 1.04 | 1.07 |

IRR: incidence rate ratios; 95% CI: 95% confidence intervals

† England only

# Supplement D Supplemental tables for self-reported CMD symptoms (USoc)

Supplement D table 1 Extended sample characteristics of complete case and full sample, 2009-10; 2014-15; 2019-20, USoc

Supplement D table 2 Self-reported CMD symptoms (mean and 95%CI), 2009-10 to 2019-20, Usoc

Supplement D table 3 Ratio of self-reported CMD symptoms over time, from 2009-10 to 2014-15, 2014-15 to 2019-20, and 2009-10 to 2019-20, Usoc

Supplement D table 4 Ratio of self-reported CMD symptoms across sociodemographic group, 2009-10 to 2019-20, Usoc

Supplement D table 5 Weighted proportion exceeding psychological distress symptom cut-off (14 or over), 2009-10 to 2019-20, Usoc

## Supplement D table 1 Sample characteristics of complete case and full sample, 2009-10; 2014-15; and 2019-20, USoc

| | 2009-10 (Wave A) | | | | | 2014-15 (Wave F) | | | | | 2019-20 (Wave K) | | | | |
|---|---|---|---|---|---|---|---|---|---|---|---|---|---|---|---|
| | Complete case | | Full sample | | p- | Complete case | | Full sample | | p- | Complete case | | Full sample | | p- |
| | n | % | n | % | value | n | % | n | % | value | n | % | n | % | value |
| Total | | | | | | | | | | | | | | | |
| | 8,248 | 100 | 10,245 | 100 | | 10,173 | 100 | 10,668 | 100 | | 6,809 | 100 | 7,081 | 100 | |
| Sex | | | | | 0.77 | | | | | 0.81 | | | | | 0.169 |
| Female | 4,619 | 56 | 5,730 | 55.9 | | 5,604 | 55.09 | 5,874 | 55.06 | | 3,842 | 56.4 | 3,984 | 56.26 | |
| Male | 3,629 | 44 | 4,515 | 44.1 | | 4,569 | 44.91 | 4,794 | 44.94 | | 2,967 | 43.6 | 3,097 | 43.74 | |
| Age group | | | | | <0.001 | | | | | <0.001 | | | | | 0.005 |
| 16-19 | 2,468 | 29.92 | 2,936 | 28.66 | | 2,334 | 22.94 | 2,419 | 22.68 | | 1,270 | 18.65 | 1,337 | 18.88 | |
| 20-24 | 2,771 | 33.6 | 3,397 | 33.16 | | 2,626 | 28.81 | 2,743 | 25.71 | | 1,434 | 21.06 | 1,506 | 21.27 | |
| 25-29 | 2,826 | 34.3 | 3,679 | 35.91 | | 2,314 | 22.75 | 2,432 | 22.8 | | 1,240 | 18.21 | 1,285 | 18.15 | |
| 30-34 | 183 | 2.2 | 233 | 2.27 | | 2,681 | 26.35 | 2,835 | 26.57 | | 1,272 | 18.68 | 1,311 | 18.51 | |
| 35-39 - | - | - | - | | | 218 | 2.14 | 239 | 2.24 | | 1,593 | 23.4 | 1,642 | 23.19 | |
| Cohort | | | | | <0.001 | | | | | 0.004 | | | | | 0.003 |
| 1980-84 | 3,016 | 36.57 | 3,899 | 38.1 | | 2,834 | 27.86 | 3,000 | 28.12 | | 1,600 | 23.5 | 1,650 | 23.3 | |
| 1985-89 | 2,762 | 33.49 | 3,400 | 33.2 | | 2,340 | 23 | 2,467 | 23.13 | | 1,263 | 18.55 | 1,301 | 18.37 | |
| 1990-94 | 2,470 | 29.95 | 2,946 | 28.8 | | 2,621 | 25.76 | 2,726 | 25.55 | | 1,241 | 18.23 | 1,284 | 18.13 | |
| 1995-99 - | - | - | - | | | 2,377 | 23.37 | 2,475 | 23.2 | | 1,439 | 21.13 | 1,515 | 21.4 | |
| 2000-03 - | - | - | - | | | | | | | | 1,266 | 18.59 | 1,331 | 18.8 | |
| Ethnicity | | | | | <0.001 | | | | | <0.001 | | | | | <0.001 |
| Asian | 1,317 | 15.97 | 1,927 | 18.81 | | 1,707 | 16.78 | 1,869 | 17.52 | | 1,066 | 15.66 | 1,152 | 16.27 | |
| Black | 491 | 5.95 | 724 | 7.07 | | 714 | 7.02 | 787 | 7.38 | | 246 | 3.61 | 256 | 3.62 | |
| Mixed | 280 | 3.39 | 354 | 3.46 | | 355 | 3.49 | 369 | 3.46 | | 230 | 3.38 | 243 | 3.43 | |
| Other | 103 | 1.25 | 155 | 1.51 | | 139 | 1.37 | 153 | 1.43 | | 50 | 0.73 | 54 | 0.76 | |
| White | 6,057 | 73.44 | 7,084 | 69.15 | | 7,258 | 71.35 | 7,460 | 69.93 | | 5,217 | 76.62 | 5,363 | 75.74 | |
| Missing | 0 | 0 | 1 | 0.01 | | 0 | 0 | 30 | 0.28 | | 0 | 0 | 13 | 0.18 | |
| Country | | | | | <0.001 | | | | | 0.001 | | | | | <0.001 |
| England | 6,934 | 84.07 | 8,767 | 85.57 | | 8,319 | 81.78 | 8,741 | 81.94 | | 5,530 | 81.22 | 5,742 | 81.09 | |
| Scotland | 576 | 6.98 | 638 | 6.23 | | 678 | 6.66 | 693 | 5.84 | | 464 | 6.81 | 470 | 5.66 | |
| Northern Ireland | 323 | 3.92 | 368 | 3.59 | | 573 | 5.63 | 611 | 5.73 | | 424 | 6.23 | 468 | 6.61 | |
| Wales | 415 | 5.03 | 472 | 4.61 | | 603 | 5.93 | 623 | 5.84 | | 391 | 5.74 | 401 | 5.66 | |
| Region † | | | | | <0.001 | | | | | <0.001 | | | | | 0.05 |
| North East | 331 | 4.77 | 427 | 4.87 | | 334 | 4.01 | 359 | 4.11 | | 211 | 3.82 | 218 | 3.8 | |
| North West | 904 | 13.04 | 1,092 | 12.46 | | 1,098 | 13.2 | 1,128 | 12.9 | | 778 | 14.07 | 802 | 13.97 | |
| Yorksh. & Humb. | 731 | 10.54 | 918 | 10.47 | | 935 | 11.24 | 977 | 11.18 | | 610 | 11.03 | 639 | 11.13 | |
| East Midlands | 629 | 9.07 | 765 | 8.73 | | 735 | 8.84 | 750 | 8.58 | | 495 | 8.95 | 512 | 8.92 | |
| West Midlands | 678 | 9.78 | 936 | 10.68 | | 954 | 11.47 | 978 | 11.19 | | 630 | 11.39 | 660 | 11.49 | |
| East of England | 688 | 9.92 | 797 | 9.09 | | 737 | 8.86 | 757 | 8.66 | | 590 | 10.67 | 609 | 10.61 | |
| London | 1,513 | 21.82 | 2,127 | 24.26 | | 1,787 | 21.48 | 2,030 | 23.22 | | 983 | 17.78 | 1,036 | 18.04 | |
| South East | 911 | 13.14 | 1,066 | 12.16 | | 1,055 | 12.68 | 1,074 | 12.29 | | 747 | 13.51 | 770 | 13.41 | |
| South West | 549 | 7.92 | 639 | 7.29 | | 681 | 8.19 | 688 | 7.87 | | 486 | 8.79 | 496 | 8.64 | |
| Deprivation † | | | | | <0.001 | | | | | <0.001 | | | | | <0.001 |
| Fifth 1 (least deprived) | 2,102 | 30.31 | 2,781 | 31.72 | | 1,053 | 110.38 | 1,074 | 12.29 | | 922 | 16.67 | 943 | 16.42 | |
| Fifth 2 | 1,583 | 22.83 | 1,932 | 22.04 | | 1,349 | 141.4 | 1,372 | 15.7 | | 1,042 | 18.84 | 1,072 | 18.67 | |
| Fifth 3 | 1,290 | 18.6 | 1,448 | 16.52 | | 1,433 | 150.21 | 1,489 | 17.03 | | 1,039 | 18.79 | 1,072 | 18.67 | |
| Fifth 4 | 1,113 | 16.05 | 1,250 | 14.26 | | 1,879 | 196.96 | 2,003 | 22.91 | | 1,207 | 21.83 | 1,254 | 21.84 | |
| Fifth 5 (most deprived) | 846 | 12.2 | 946 | 10.79 | | 2,605 | 273.06 | 2,803 | 32.07 | | 1,320 | 23.87 | 1,401 | 24.4 | |
| Missing | 0 | 0 | 410 | 4.68 | | 0 | 0 | 422 | 4.83 | | 0 | 0 | 422 | 7.35 | |

† England only

**Supplement D table 2** **Self-reported psychological distress symptoms (mean and 95%CI), 2009-10 to 2019-20, Usoc**

| Variable | 2009-10 | | | 2010-11 | | | 2011-12 | | | 2012-13 | | |
|---|---|---|---|---|---|---|---|---|---|---|---|---|
| | Mean | 95% CI | | Mean | 95% CI | | Mean | 95% CI | | Mean | 95% CI | |
| Total | 10.47 | 10.34 | 10.59 | 10.60 | 10.47 | 10.74 | 10.93 | 10.80 | 11.07 | 10.90 | 10.75 | 11.05 |
| Sex | | | | | | | | | | | | |
| Female | 11.12 | 10.96 | 11.28 | 11.19 | 11.01 | 11.37 | 11.83 | 11.64 | 12.02 | 11.83 | 11.62 | 12.04 |
| Male | 9.85 | 9.67 | 10.03 | 9.93 | 9.73 | 10.12 | 10.07 | 9.88 | 10.27 | 9.99 | 9.79 | 10.20 |
| Age group | | | | | | | | | | | | |
| 16-19 | 9.63 | 9.42 | 9.84 | 9.86 | 9.63 | 10.09 | 10.42 | 10.18 | 10.67 | 10.46 | 10.19 | 10.73 |
| 20-24 | 10.83 | 10.62 | 11.04 | 10.66 | 10.41 | 10.92 | 11.08 | 10.82 | 11.34 | 10.76 | 10.47 | 11.05 |
| 25-29 | 10.75 | 10.54 | 10.97 | 11.03 | 10.78 | 11.27 | 11.18 | 10.92 | 11.43 | 11.28 | 10.98 | 11.58 |
| 30-34 | 10.56 | 9.89 | 11.22 | 11.05 | 10.58 | 11.52 | 11.17 | 10.81 | 11.54 | 11.14 | 10.81 | 11.47 |
| 35-39 | - | - | - | - | - | - | - | - | - | - | - | - |
| Cohort | | | | | | | | | | | | |
| 1980-84 | 10.72 | 10.52 | 10.92 | 11.06 | 10.83 | 11.29 | 11.16 | 10.92 | 11.40 | 11.12 | 10.86 | 11.38 |
| 1985-89 | 10.81 | 10.60 | 11.03 | 10.78 | 10.53 | 11.03 | 11.19 | 10.92 | 11.46 | 11.34 | 11.03 | 11.65 |
| 1990-94 | 9.70 | 9.48 | 9.92 | 9.97 | 9.75 | 10.19 | 10.62 | 10.39 | 10.85 | 10.50 | 10.24 | 10.76 |
| 1995-99 | - | - | - | 9.61 | 8.89 | 10.32 | 10.29 | 9.80 | 10.78 | 10.38 | 9.99 | 10.76 |
| 2000-03 | - | - | - | - | - | - | - | - | - | - | - | - |
| Ethnicity | | | | | | | | | | | | |
| Asian | 10.21 | 9.86 | 10.56 | 10.14 | 9.70 | 10.58 | 10.54 | 10.13 | 10.95 | 10.83 | 10.33 | 11.32 |
| Black | 9.93 | 9.39 | 10.47 | 11.02 | 10.39 | 11.65 | 10.92 | 10.26 | 11.58 | 10.89 | 10.17 | 11.60 |
| Mixed | 11.66 | 10.94 | 12.38 | 11.10 | 10.23 | 11.97 | 11.46 | 10.69 | 12.22 | 10.64 | 9.70 | 11.58 |
| Other | 11.30 | 10.06 | 12.53 | 10.96 | 9.61 | 12.31 | 10.93 | 9.49 | 12.37 | 9.89 | 8.34 | 11.44 |
| White | 10.47 | 10.34 | 10.61 | 10.61 | 10.47 | 10.76 | 10.95 | 10.80 | 11.10 | 10.91 | 10.75 | 11.08 |
| Country | | | | | | | | | | | | |
| England | 10.48 | 10.35 | 10.62 | 10.61 | 10.46 | 10.75 | 10.95 | 10.79 | 11.10 | 10.96 | 10.79 | 11.12 |
| Scotland | 10.37 | 9.93 | 10.80 | 10.53 | 10.06 | 11.00 | 10.91 | 10.45 | 11.36 | 10.50 | 9.99 | 11.00 |
| Northern Ireland | 10.30 | 9.71 | 10.88 | 10.18 | 9.57 | 10.79 | 10.29 | 9.86 | 10.71 | 9.88 | 9.43 | 10.33 |
| Wales | 10.49 | 9.99 | 11.00 | 10.89 | 10.20 | 11.58 | 11.06 | 10.59 | 11.54 | 11.09 | 10.59 | 11.60 |
| Region † | | | | | | | | | | | | |
| North East | 10.02 | 9.51 | 10.53 | 10.79 | 10.09 | 11.49 | 10.59 | 9.87 | 11.31 | 10.87 | 10.18 | 11.57 |
| North West | 10.56 | 10.20 | 10.93 | 10.61 | 10.24 | 10.99 | 10.98 | 10.58 | 11.38 | 11.22 | 10.76 | 11.69 |
| Yorksh. & Humb. | 10.01 | 9.63 | 10.39 | 10.84 | 10.38 | 11.30 | 10.89 | 10.45 | 11.34 | 10.89 | 10.38 | 11.39 |
| East Midlands | 10.54 | 10.13 | 10.95 | 10.54 | 10.11 | 10.97 | 10.86 | 10.42 | 11.30 | 10.81 | 10.36 | 11.25 |
| West Midlands | 10.84 | 10.41 | 11.26 | 10.55 | 10.08 | 11.02 | 11.02 | 10.53 | 11.51 | 11.00 | 10.47 | 11.53 |
| East of England | 10.75 | 10.35 | 11.15 | 10.59 | 10.16 | 11.03 | 10.93 | 10.47 | 11.39 | 11.34 | 10.82 | 11.86 |
| London | 10.58 | 10.22 | 10.93 | 10.56 | 10.15 | 10.96 | 10.83 | 10.40 | 11.25 | 10.72 | 10.27 | 11.17 |
| South East | 10.35 | 10.01 | 10.68 | 10.69 | 10.33 | 11.05 | 11.22 | 10.83 | 11.61 | 10.83 | 10.42 | 11.23 |
| South West | 10.49 | 10.05 | 10.92 | 10.30 | 9.84 | 10.75 | 10.94 | 10.43 | 11.46 | 10.93 | 10.42 | 11.44 |
| Deprivation † | | | | | | | | | | | | |
| Fifth 1 (least deprived) | 10.24 | 9.90 | 10.58 | 10.22 | 9.87 | 10.57 | 10.62 | 10.25 | 10.98 | 10.53 | 10.15 | 10.92 |
| Fifth 2 | 10.34 | 10.04 | 10.64 | 10.40 | 10.07 | 10.72 | 10.94 | 10.60 | 11.27 | 11.07 | 10.72 | 11.42 |
| Fifth 3 | 10.53 | 10.23 | 10.84 | 10.33 | 10.01 | 10.65 | 10.80 | 10.46 | 11.14 | 10.90 | 10.55 | 11.25 |
| Fifth 4 | 10.62 | 10.34 | 10.90 | 10.73 | 10.42 | 11.04 | 10.78 | 10.46 | 11.11 | 11.01 | 10.62 | 11.41 |
| Fifth 5 (most deprived) | 10.57 | 10.30 | 10.84 | 11.12 | 10.79 | 11.45 | 11.43 | 11.09 | 11.76 | 11.13 | 10.77 | 11.49 |

Mean: weighted mean CMD score on imputed data; 95% CI: 95% confidence intervals;
† England only

## Supplement D table 2 (pg 2) Self-reported psychological distress symptoms (mean and 95%CI), 2009-10 to 2019-20, Usoc

| Variable | 2013-14 | | | 2014-15 | | | 2015-16 | | | 2016-17 | | |
|---|---|---|---|---|---|---|---|---|---|---|---|---|
| | Mean | 95% CI | | Mean | 95% CI | | Mean | 95% CI | | Mean | 95% CI | |
| Total | | | | | | | | | | | | |
| | 10.91 | 10.76 | 11.05 | 10.79 | 10.66 | 10.92 | 11.00 | 10.86 | 11.15 | 11.28 | 11.12 | 11.43 |
| Sex | | | | | | | | | | | | |
| Female | 11.71 | 11.51 | 11.91 | 11.49 | 11.30 | 11.68 | 11.76 | 11.56 | 11.96 | 12.00 | 11.79 | 12.22 |
| Male | 10.13 | 9.94 | 10.33 | 10.09 | 9.90 | 10.27 | 10.23 | 10.03 | 10.44 | 10.52 | 10.30 | 10.74 |
| Age group | | | | | | | | | | | | |
| 16-19 | 10.54 | 10.28 | 10.81 | 10.56 | 10.28 | 10.83 | 10.66 | 10.37 | 10.95 | 10.92 | 10.62 | 11.23 |
| 20-24 | 10.78 | 10.51 | 11.05 | 10.80 | 10.54 | 11.06 | 10.87 | 10.58 | 11.17 | 11.22 | 10.90 | 11.55 |
| 25-29 | 11.37 | 11.07 | 11.68 | 11.02 | 10.73 | 11.31 | 11.26 | 10.92 | 11.61 | 11.43 | 11.07 | 11.79 |
| 30-34 | 10.95 | 10.68 | 11.23 | 10.78 | 10.52 | 11.03 | 11.11 | 10.82 | 11.39 | 11.38 | 11.05 | 11.71 |
| 35-39 | - | - | - | 10.58 | 9.58 | 11.58 | 11.41 | 10.83 | 11.98 | 11.59 | 11.12 | 12.06 |
| Cohort | | | | | | | | | | | | |
| 1980-84 | 11.02 | 10.77 | 11.27 | 10.82 | 10.57 | 11.07 | 11.25 | 10.98 | 11.53 | 11.53 | 11.21 | 11.84 |
| 1985-89 | 11.20 | 10.90 | 11.51 | 10.94 | 10.66 | 11.23 | 11.14 | 10.80 | 11.49 | 11.48 | 11.12 | 11.83 |
| 1990-94 | 10.71 | 10.45 | 10.97 | 10.82 | 10.56 | 11.09 | 10.82 | 10.54 | 11.11 | 11.16 | 10.83 | 11.50 |
| 1995-99 | 10.64 | 10.33 | 10.95 | 10.55 | 10.28 | 10.82 | 10.83 | 10.55 | 11.10 | 11.08 | 10.78 | 11.37 |
| 2000-03 | - | - | - | - | - | - | 10.54 | 9.37 | 11.72 | 10.71 | 10.07 | 11.35 |
| Ethnicity | | | | | | | | | | | | |
| Asian | 10.62 | 10.13 | 11.12 | 9.86 | 9.48 | 10.24 | 10.18 | 9.75 | 10.61 | 10.04 | 9.60 | 10.49 |
| Black | 11.09 | 10.34 | 11.83 | 9.90 | 9.20 | 10.59 | 10.04 | 9.36 | 10.73 | 9.99 | 9.27 | 10.71 |
| Mixed | 12.14 | 11.13 | 13.14 | 11.66 | 10.71 | 12.61 | 12.31 | 11.27 | 13.35 | 12.36 | 11.33 | 13.40 |
| Other | 10.21 | 8.77 | 11.65 | 11.16 | 9.71 | 12.61 | 11.32 | 9.65 | 12.99 | 12.50 | 10.75 | 14.26 |
| White | 10.89 | 10.74 | 11.05 | 10.87 | 10.72 | 11.01 | 11.07 | 10.91 | 11.23 | 11.37 | 11.20 | 11.53 |
| Country | | | | | | | | | | | | |
| England | 10.90 | 10.75 | 11.06 | 10.78 | 10.64 | 10.93 | 11.04 | 10.88 | 11.20 | 11.26 | 11.09 | 11.43 |
| Scotland | 10.84 | 10.36 | 11.31 | 11.04 | 10.56 | 11.52 | 11.00 | 10.50 | 11.49 | 11.66 | 11.06 | 12.25 |
| Northern Ireland | 10.39 | 9.89 | 10.89 | 10.04 | 9.61 | 10.46 | 9.94 | 9.45 | 10.43 | 9.72 | 9.18 | 10.26 |
| Wales | 11.31 | 10.78 | 11.83 | 10.90 | 10.40 | 11.40 | 11.03 | 10.50 | 11.57 | 11.92 | 11.30 | 12.53 |
| Region † | | | | | | | | | | | | |
| North East | 10.79 | 10.06 | 11.52 | 10.66 | 10.04 | 11.28 | 10.70 | 9.94 | 11.46 | 11.40 | 10.53 | 12.27 |
| North West | 11.15 | 10.70 | 11.61 | 10.90 | 10.48 | 11.33 | 11.07 | 10.63 | 11.51 | 11.48 | 10.98 | 11.98 |
| Yorksh. & Humb. | 10.77 | 10.32 | 11.22 | 10.62 | 10.21 | 11.04 | 10.85 | 10.32 | 11.37 | 11.01 | 10.51 | 11.52 |
| East Midlands | 10.74 | 10.28 | 11.19 | 11.08 | 10.61 | 11.56 | 10.96 | 10.51 | 11.41 | 11.11 | 10.59 | 11.63 |
| West Midlands | 11.03 | 10.55 | 11.52 | 10.80 | 10.35 | 11.25 | 11.32 | 10.82 | 11.82 | 11.50 | 11.00 | 12.00 |
| East of England | 10.72 | 10.28 | 11.15 | 10.85 | 10.39 | 11.31 | 11.43 | 10.90 | 11.96 | 11.48 | 10.92 | 12.04 |
| London | 10.85 | 10.40 | 11.30 | 10.42 | 10.05 | 10.79 | 10.63 | 10.20 | 11.06 | 10.65 | 10.21 | 11.10 |
| South East | 11.13 | 10.73 | 11.53 | 10.93 | 10.54 | 11.32 | 11.17 | 10.79 | 11.56 | 11.53 | 11.13 | 11.94 |
| South West | 10.78 | 10.29 | 11.26 | 10.86 | 10.38 | 11.35 | 11.20 | 10.67 | 11.73 | 11.32 | 10.78 | 11.86 |
| Deprivation † | | | | | | | | | | | | |
| Fifth 1 (least deprived) | 10.53 | 10.17 | 10.89 | 10.31 | 9.95 | 10.67 | 10.70 | 10.35 | 11.05 | 11.11 | 10.74 | 11.48 |
| Fifth 2 | 10.50 | 10.17 | 10.82 | 10.63 | 10.33 | 10.94 | 10.55 | 10.23 | 10.88 | 10.64 | 10.30 | 10.97 |
| Fifth 3 | 11.03 | 10.68 | 11.38 | 10.77 | 10.43 | 11.10 | 11.18 | 10.81 | 11.56 | 11.15 | 10.80 | 11.51 |
| Fifth 4 | 10.97 | 10.63 | 11.30 | 10.95 | 10.61 | 11.28 | 11.11 | 10.72 | 11.50 | 11.56 | 11.16 | 11.96 |
| Fifth 5 (most deprived) | 11.36 | 11.00 | 11.73 | 11.09 | 10.78 | 11.41 | 11.47 | 11.12 | 11.82 | 11.69 | 11.29 | 12.10 |

Mean: weighted mean CMD score on imputed data; 95% CI: 95% confidence intervals;
† England only

**Supplement D table 2 (pg 3) Self-reported psychological distress symptoms (mean and 95%CI), 2009-10 to 2019-20, Usoc**

| Variable | 2017-18 Mean | 95% CI | | 2018-19 Mean | 95% CI | | 2019-20 Mean | 95% CI | |
|---|---|---|---|---|---|---|---|---|---|
| Total | | | | | | | | | |
| | 11.87 | 11.70 | 12.04 | 12.10 | 11.92 | 12.28 | 12.49 | 12.30 | 12.68 |
| Sex | | | | | | | | | |
| Female | 12.64 | 12.40 | 12.87 | 12.87 | 12.63 | 13.11 | 13.23 | 12.97 | 13.48 |
| Male | 11.05 | 10.80 | 11.30 | 11.28 | 11.02 | 11.53 | 11.67 | 11.40 | 11.95 |
| Age group | | | | | | | | | |
| 16-19 | 11.79 | 11.43 | 12.16 | 12.19 | 11.80 | 12.58 | 11.96 | 11.56 | 12.36 |
| 20-24 | 11.85 | 11.51 | 12.20 | 11.94 | 11.58 | 12.31 | 12.76 | 12.36 | 13.17 |
| 25-29 | 11.68 | 11.31 | 12.06 | 11.88 | 11.50 | 12.26 | 12.42 | 11.98 | 12.85 |
| 30-34 | 12.16 | 11.76 | 12.57 | 12.45 | 12.00 | 12.89 | 12.77 | 12.31 | 13.24 |
| 35-39 | 11.84 | 11.40 | 12.28 | 12.05 | 11.65 | 12.45 | 12.46 | 12.05 | 12.87 |
| Cohort | | | | | | | | | |
| 1980-84 | 11.95 | 11.61 | 12.29 | 12.07 | 11.72 | 12.42 | 12.42 | 12.01 | 12.82 |
| 1985-89 | 12.19 | 11.75 | 12.62 | 12.42 | 11.96 | 12.88 | 12.81 | 12.34 | 13.28 |
| 1990-94 | 11.64 | 11.30 | 11.98 | 11.84 | 11.46 | 12.21 | 12.37 | 11.95 | 12.79 |
| 1995-99 | 11.81 | 11.47 | 12.15 | 12.00 | 11.64 | 12.37 | 12.81 | 12.39 | 13.22 |
| 2000-03 | 11.70 | 11.18 | 12.22 | 12.24 | 11.80 | 12.69 | 11.96 | 11.57 | 12.36 |
| Ethnicity | | | | | | | | | |
| Asian | 10.50 | 10.01 | 10.99 | 10.88 | 10.39 | 11.37 | 11.59 | 11.10 | 12.09 |
| Black | 10.26 | 9.30 | 11.23 | 10.61 | 9.65 | 11.57 | 11.33 | 10.07 | 12.59 |
| Mixed | 12.53 | 11.37 | 13.69 | 12.52 | 11.23 | 13.81 | 14.08 | 12.72 | 15.45 |
| Other | 13.12 | 10.57 | 15.68 | 12.85 | 10.43 | 15.27 | 10.69 | 8.62 | 12.75 |
| White | 11.99 | 11.80 | 12.18 | 12.21 | 12.02 | 12.40 | 12.56 | 12.35 | 12.76 |
| Country | | | | | | | | | |
| England | 11.91 | 11.72 | 12.10 | 12.13 | 11.94 | 12.33 | 12.54 | 12.33 | 12.75 |
| Scotland | 11.91 | 11.33 | 12.50 | 11.90 | 11.29 | 12.50 | 12.06 | 11.37 | 12.75 |
| Northern Ireland | 10.41 | 9.80 | 11.03 | 11.22 | 10.43 | 12.02 | 11.50 | 10.87 | 12.13 |
| Wales | 12.03 | 11.39 | 12.68 | 12.33 | 11.65 | 13.00 | 12.89 | 12.13 | 13.66 |
| Region † | | | | | | | | | |
| North East | 12.08 | 11.20 | 12.96 | 12.20 | 11.34 | 13.06 | 12.46 | 11.51 | 13.42 |
| North West | 12.28 | 11.71 | 12.86 | 12.06 | 11.56 | 12.57 | 12.53 | 11.97 | 13.09 |
| Yorksh. & Humb. | 12.07 | 11.48 | 12.67 | 12.25 | 11.64 | 12.86 | 12.58 | 11.94 | 13.22 |
| East Midlands | 11.34 | 10.85 | 11.82 | 11.77 | 11.21 | 12.33 | 12.40 | 11.80 | 12.99 |
| West Midlands | 11.70 | 11.14 | 12.27 | 12.16 | 11.52 | 12.81 | 12.63 | 12.01 | 13.25 |
| East of England | 11.75 | 11.17 | 12.33 | 12.18 | 11.60 | 12.76 | 12.24 | 11.60 | 12.88 |
| London | 11.31 | 10.79 | 11.83 | 11.59 | 11.04 | 12.14 | 12.34 | 11.74 | 12.95 |
| South East | 12.29 | 11.82 | 12.76 | 12.81 | 12.29 | 13.33 | 12.93 | 12.39 | 13.47 |
| South West | 12.39 | 11.79 | 12.99 | 12.08 | 11.54 | 12.63 | 12.62 | 12.01 | 13.23 |
| Deprivation † | | | | | | | | | |
| Fifth 1 (least deprived) | 11.52 | 11.11 | 11.93 | 11.88 | 11.47 | 12.30 | 12.10 | 11.64 | 12.56 |
| Fifth 2 | 11.59 | 11.22 | 11.97 | 11.96 | 11.56 | 12.35 | 12.24 | 11.85 | 12.64 |
| Fifth 3 | 12.13 | 11.67 | 12.59 | 11.88 | 11.44 | 12.32 | 12.72 | 12.25 | 13.18 |
| Fifth 4 | 12.07 | 11.64 | 12.51 | 12.44 | 11.97 | 12.91 | 12.81 | 12.34 | 13.28 |
| Fifth 5 (most deprived) | 12.14 | 11.70 | 12.58 | 12.41 | 11.96 | 12.86 | 12.75 | 12.24 | 13.26 |

Mean: weighted mean CMD score on imputed data; 95% CI: 95% confidence intervals;
† England only

**Supplement D Table 3** **Ratio of self-reported psychological distress symptoms (mean and 95%CI) over time, from 2009-10 to 2014-15; 2014-15 to 2019-20; and 2009-10 to 2019-20, USoc**

| | 2014 : 2009 | | | 2019 : 2014 | | | 2019 : 2009 | | | Interaction‡ |
|---|---|---|---|---|---|---|---|---|---|---|
| | Ratio | 95% CI | | Ratio | 95% CI | | Ratio | 95% CI | | chi-square (df*); p-value |
| Total | | | | | | | | | | |
| Overall | 1.03 | 1.01 | 1.05 | 1.16 | 1.14 | 1.18 | 1.19 | 1.17 | 1.22 | |
| Sex | | | | | | | | | | 0.35 (1); |
| Female | 1.03 | 1.01 | 1.06 | 1.15 | 1.12 | 1.18 | 1.19 | 1.16 | 1.22 | 0.55 |
| Male | 1.02 | 1.00 | 1.05 | 1.16 | 1.12 | 1.19 | 1.19 | 1.15 | 1.22 | |
| Age group | | | | | | | | | | 35 (4); |
| 16-19 | 1.10 | 1.06 | 1.13 | 1.13 | 1.09 | 1.18 | 1.24 | 1.19 | 1.29 | <0.001 |
| 20-24 | 1.00 | 0.97 | 1.03 | 1.18 | 1.13 | 1.23 | 1.18 | 1.13 | 1.22 | |
| 25-29 | 1.02 | 0.99 | 1.06 | 1.13 | 1.08 | 1.18 | 1.15 | 1.11 | 1.20 | |
| 30-34 | 1.02 | 0.95 | 1.09 | 1.19 | 1.13 | 1.24 | 1.21 | 1.12 | 1.30 | |
| 35-39° | - | - | - | 1.18 | 1.06 | 1.30 | 1.18 | 1.06 | 1.30 ° | |
| Cohort | | | | | | | | | | 105 (4); |
| 1980-84 | 1.01 | 0.98 | 1.04 | 1.15 | 1.10 | 1.19 | 1.16 | 1.12 | 1.20 | <0.001 |
| 1985-89 | 1.01 | 0.98 | 1.04 | 1.17 | 1.12 | 1.22 | 1.18 | 1.14 | 1.23 | |
| 1990-94 | 1.12 | 1.08 | 1.15 | 1.14 | 1.10 | 1.19 | 1.28 | 1.22 | 1.33 | |
| 1995-99^ | 1.10 | 1.01 | 1.19 | 1.21 | 1.16 | 1.26 | 1.33 | 1.22 | 1.44 ^ | |
| 2000-03± | - | - | - | 1.13 | 1.00 | 1.27 | 1.13 | 1.00 | 1.27 ± | |
| Ethnicity | | | | | | | | | | 46 (4); |
| Asian | 0.97 | 0.92 | 1.02 | 1.18 | 1.11 | 1.24 | 1.14 | 1.07 | 1.20 | <0.001 |
| Black | 1.00 | 0.91 | 1.09 | 1.14 | 0.99 | 1.30 | 1.14 | 1.00 | 1.28 | |
| Mixed | 1.00 | 0.90 | 1.10 | 1.21 | 1.06 | 1.36 | 1.21 | 1.07 | 1.35 | |
| Other | 0.99 | 0.82 | 1.16 | 0.96 | 0.74 | 1.18 | 0.95 | 0.74 | 1.16 | |
| White | 1.04 | 1.02 | 1.06 | 1.16 | 1.13 | 1.18 | 1.20 | 1.17 | 1.22 | |
| Country | | | | | | | | | | 8 (3); |
| England | 1.03 | 1.01 | 1.05 | 1.16 | 1.14 | 1.19 | 1.20 | 1.17 | 1.22 | 0.05 |
| Scotland | 1.07 | 1.00 | 1.14 | 1.09 | 1.01 | 1.17 | 1.17 | 1.08 | 1.26 | |
| Northern Ireland | 0.96 | 0.90 | 1.01 | 1.15 | 1.07 | 1.23 | 1.10 | 1.02 | 1.17 | |
| Wales | 1.05 | 0.98 | 1.13 | 1.18 | 1.09 | 1.27 | 1.24 | 1.14 | 1.34 | |
| Region † | | | | | | | | | | 39 (11); |
| North East | 1.06 | 0.98 | 1.15 | 1.17 | 1.06 | 1.28 | 1.24 | 1.13 | 1.36 | <0.001 |
| North West | 1.03 | 0.98 | 1.09 | 1.15 | 1.08 | 1.22 | 1.19 | 1.12 | 1.25 | |
| Yorksh. & Humb. | 1.06 | 1.00 | 1.12 | 1.18 | 1.11 | 1.26 | 1.26 | 1.18 | 1.34 | |
| East Midlands | 1.05 | 0.99 | 1.11 | 1.12 | 1.05 | 1.19 | 1.18 | 1.10 | 1.25 | |
| West Midlands | 1.00 | 0.94 | 1.05 | 1.17 | 1.09 | 1.24 | 1.17 | 1.09 | 1.24 | |
| East of England | 1.01 | 0.95 | 1.07 | 1.13 | 1.05 | 1.20 | 1.14 | 1.07 | 1.21 | |
| London | 0.99 | 0.94 | 1.03 | 1.18 | 1.11 | 1.26 | 1.17 | 1.10 | 1.24 | |
| South East | 1.06 | 1.01 | 1.11 | 1.18 | 1.12 | 1.25 | 1.25 | 1.18 | 1.32 | |
| South West | 1.04 | 0.97 | 1.10 | 1.16 | 1.09 | 1.24 | 1.20 | 1.13 | 1.28 | |
| Deprivation † | | | | | | | | | | 9 (5); |
| Fifth 1 (least deprived) | 1.01 | 0.96 | 1.05 | 1.17 | 1.11 | 1.23 | 1.18 | 1.12 | 1.24 | 0.10 |
| Fifth 2 | 1.03 | 0.99 | 1.07 | 1.15 | 1.10 | 1.20 | 1.18 | 1.13 | 1.24 | |
| Fifth 3 | 1.02 | 0.98 | 1.07 | 1.18 | 1.12 | 1.24 | 1.21 | 1.15 | 1.26 | |
| Fifth 4 | 1.03 | 0.99 | 1.07 | 1.17 | 1.11 | 1.23 | 1.21 | 1.15 | 1.26 | |
| Fifth 5 (most deprived) | 1.05 | 1.01 | 1.09 | 1.15 | 1.09 | 1.21 | 1.21 | 1.15 | 1.26 | |

RR: ratio of mean scores; 95% CI: 95% confidence intervals;

° Used earliest estimate (2014-15); ^ Used earliest estimate (2010-11); ± Used earliest estimate (2015-16)

† England only

‡ Likelihood ratio test (LRT) for interaction

* Degrees of freedom (df)

## Supplement D table 4 — Ratio of self-reported psychological distress symptoms across sociodemographic group, 2009-10 to 2019-20, Usoc

| Variable | 2009-10 | | | 2010-11 | | | 2011-12 | | | 2012-13 | | |
|---|---|---|---|---|---|---|---|---|---|---|---|---|
| | Ratio | 95% CI | | Ratio | 95% CI | | Ratio | 95% CI | | Ratio | 95% CI | |
| Sex | | | | | | | | | | | | |
| Female | 1.13 | 1.10 | 1.16 | 1.13 | 1.10 | 1.16 | 1.17 | 1.15 | 1.20 | 1.18 | 1.15 | 1.22 |
| Male (ref.) | 1.00 | | | 1.00 | | | 1.00 | | | 1.00 | | |
| Age group | | | | | | | | | | | | |
| 16-19 | 0.90 | 0.87 | 0.92 | 0.89 | 0.87 | 0.92 | 0.93 | 0.90 | 0.96 | 0.93 | 0.89 | 0.96 |
| 20-24 | 1.01 | 0.98 | 1.04 | 0.97 | 0.94 | 1.00 | 0.99 | 0.96 | 1.02 | 0.95 | 0.92 | 0.99 |
| 25-29 (ref.) | 1.00 | | | 1.00 | | | 1.00 | | | 1.00 | | |
| 30-34 | 0.98 | 0.92 | 1.05 | 1.00 | 0.95 | 1.05 | 1.00 | 0.96 | 1.04 | 0.99 | 0.95 | 1.03 |
| 35-39 | - | - | - | - | - | - | - | - | - | | | |
| Cohort | | | | | | | | | | | | |
| 1980-84 (ref.) | 1.00 | | | 1.00 | | | 1.00 | | | 1.00 | | |
| 1985-89 | 1.01 | 0.98 | 1.04 | 0.97 | 0.94 | 1.00 | 1.00 | 0.97 | 1.03 | 1.02 | 0.98 | 1.06 |
| 1990-94 | 0.90 | 0.88 | 0.93 | 0.90 | 0.87 | 0.93 | 0.95 | 0.92 | 0.98 | 0.94 | 0.91 | 0.98 |
| 1995-99 | - | - | - | 0.87 | 0.80 | 0.94 | 0.92 | 0.87 | 0.97 | 0.93 | 0.89 | 0.97 |
| 2000-03 | - | - | - | - | - | - | - | - | - | - | - | - |
| Ethnicity | | | | | | | | | | | | |
| Asian | 0.97 | 0.94 | 1.01 | 0.96 | 0.91 | 1.00 | 0.96 | 0.92 | 1.00 | 0.99 | 0.94 | 1.04 |
| Black | 0.95 | 0.89 | 1.00 | 1.04 | 0.98 | 1.10 | 1.00 | 0.94 | 1.06 | 1.00 | 0.93 | 1.06 |
| Mixed | 1.11 | 1.04 | 1.18 | 1.05 | 0.96 | 1.13 | 1.05 | 0.97 | 1.12 | 0.97 | 0.89 | 1.06 |
| Other | 1.08 | 0.96 | 1.20 | 1.03 | 0.90 | 1.16 | 1.00 | 0.87 | 1.13 | 0.91 | 0.76 | 1.05 |
| White (ref.) | 1.00 | | | 1.00 | | | 1.00 | | | 1.00 | | |
| Country | | | | | | | | | | | | |
| England (ref.) | 1.00 | | | 1.00 | | | 1.00 | | | 1.00 | | |
| Scotland | 0.98 | 0.93 | 1.03 | 0.99 | 0.95 | 1.04 | 1.00 | 0.95 | 1.04 | 0.96 | 0.91 | 1.01 |
| Northern Ireland | 1.00 | 0.96 | 1.04 | 0.96 | 0.90 | 1.02 | 0.94 | 0.90 | 0.98 | 0.90 | 0.86 | 0.94 |
| Wales | 0.99 | 0.93 | 1.05 | 1.03 | 0.96 | 1.09 | 1.01 | 0.96 | 1.06 | 1.01 | 0.96 | 1.06 |
| Region † | | | | | | | | | | | | |
| North East | 0.95 | 0.89 | 1.00 | 1.02 | 0.94 | 1.10 | 0.98 | 0.90 | 1.05 | 1.01 | 0.94 | 1.09 |
| North West | 1.00 | 0.95 | 1.05 | 1.01 | 0.95 | 1.06 | 1.01 | 0.96 | 1.07 | 1.05 | 0.98 | 1.11 |
| Yorksh. & Humb. | 1.00 | 0.95 | 1.05 | 1.00 | 0.95 | 1.06 | 1.03 | 0.97 | 1.09 | 1.00 | 0.94 | 1.06 |
| East Midlands | 1.05 | 1.00 | 1.10 | 0.98 | 0.92 | 1.03 | 1.03 | 0.97 | 1.08 | 0.99 | 0.93 | 1.05 |
| West Midlands | 1.08 | 1.03 | 1.13 | 0.98 | 0.92 | 1.04 | 1.04 | 0.98 | 1.10 | 1.01 | 0.95 | 1.08 |
| East of England | 1.07 | 1.02 | 1.12 | 0.98 | 0.93 | 1.04 | 1.03 | 0.97 | 1.09 | 1.04 | 0.98 | 1.11 |
| London (ref.) | 1.00 | | 1.00 | 1.00 | | | 1.00 | | | 1.00 | | |
| South East | 1.03 | 0.99 | 1.08 | 0.99 | 0.94 | 1.04 | 1.06 | 1.01 | 1.11 | 1.00 | 0.94 | 1.05 |
| South West | 1.05 | 0.99 | 1.10 | 0.95 | 0.90 | 1.01 | 1.03 | 0.97 | 1.09 | 1.01 | 0.94 | 1.07 |
| Deprivation † | | | | | | | | | | | | |
| ref.) | 1.00 | | | 1.00 | | | 1.00 | | | 1.00 | | |
| Fifth 2 | 1.01 | 0.96 | 1.05 | 1.02 | 0.97 | 1.06 | 1.03 | 0.98 | 1.08 | 1.05 | 1.00 | 1.10 |
| Fifth 3 | 1.03 | 0.98 | 1.07 | 1.01 | 0.96 | 1.06 | 1.02 | 0.97 | 1.06 | 1.03 | 0.98 | 1.09 |
| Fifth 4 | 1.04 | 0.99 | 1.08 | 1.05 | 1.00 | 1.10 | 1.02 | 0.97 | 1.06 | 1.05 | 0.99 | 1.10 |
| Fifth 5 (most deprived) | 1.03 | 0.99 | 1.08 | 1.09 | 1.04 | 1.14 | 1.08 | 1.03 | 1.13 | 1.06 | 1.01 | 1.11 |

95% CI: 95% confidence intervals;

† England only

**Supplement D table 4** **(pg 2) Ratio of self-reported psychological distress symptoms across sociodemographic group, 2009-10 to 2019-20, Usoc**

| Variable | 2013-14 | | | 2014-15 | | | 2015-16 | | | 2016-17 | | |
|---|---|---|---|---|---|---|---|---|---|---|---|---|
| | Ratio | 95% CI | | Ratio | 95% CI | | Ratio | 95% CI | | Ratio | 95% CI | |
| Sex | | | | | | | | | | | | |
| Female | 1.16 | 1.13 | 1.18 | 1.14 | 1.11 | 1.17 | 1.15 | 1.12 | 1.18 | 1.14 | 1.11 | 1.17 |
| Male (ref.) | 1.00 | | | 1.00 | | | 1.00 | | | 1.00 | | |
| Age group | | | | | | | | | | | | |
| 16-19 | 0.93 | 0.89 | 0.96 | 0.96 | 0.92 | 0.99 | 0.95 | 0.91 | 0.99 | 0.96 | 0.92 | 1.00 |
| 20-24 | 0.95 | 0.91 | 0.98 | 0.98 | 0.95 | 1.02 | 0.97 | 0.93 | 1.00 | 0.98 | 0.94 | 1.02 |
| 25-29 (ref.) | 1.00 | | | 1.00 | | | 1.00 | | | 1.00 | | |
| 30-34 | 0.96 | 0.93 | 1.00 | 0.98 | 0.94 | 1.01 | 0.99 | 0.95 | 1.03 | 1.00 | 0.95 | 1.04 |
| 35-39 | - | - | - | 0.96 | 0.87 | 1.05 | 1.01 | 0.95 | 1.07 | 1.01 | 0.96 | 1.07 |
| Cohort | | | | | | | | | | | | |
| 1980-84 (ref.) | 1.00 | | | 1.00 | | | 1.00 | | | 1.00 | | |
| 1985-89 | 1.02 | 0.98 | 1.05 | 1.01 | 0.98 | 1.05 | 0.99 | 0.95 | 1.03 | 1.00 | 0.95 | 1.04 |
| 1990-94 | 0.97 | 0.94 | 1.00 | 1.00 | 0.97 | 1.03 | 0.96 | 0.93 | 1.00 | 0.97 | 0.93 | 1.01 |
| 1995-99 | 0.97 | 0.93 | 1.00 | 0.97 | 0.94 | 1.01 | 0.96 | 0.93 | 1.00 | 0.96 | 0.92 | 1.00 |
| 2000-03 | - | - | - | - | - | - | 0.94 | 0.83 | 1.04 | 0.93 | 0.87 | 0.99 |
| Ethnicity | | | | | | | | | | | | |
| Asian | 0.98 | 0.93 | 1.02 | 0.91 | 0.87 | 0.94 | 0.92 | 0.88 | 0.96 | 0.88 | 0.84 | 0.92 |
| Black | 1.02 | 0.95 | 1.09 | 0.91 | 0.85 | 0.98 | 0.91 | 0.84 | 0.97 | 0.88 | 0.81 | 0.94 |
| Mixed | 1.11 | 1.02 | 1.21 | 1.07 | 0.98 | 1.16 | 1.11 | 1.02 | 1.21 | 1.09 | 1.00 | 1.18 |
| Other | 0.94 | 0.80 | 1.07 | 1.03 | 0.89 | 1.16 | 1.02 | 0.87 | 1.17 | 1.10 | 0.94 | 1.26 |
| White (ref.) | 1.00 | | | 1.00 | | | 1.00 | | | 1.00 | | |
| Country | | | | | | | | | | | | |
| England (ref.) | 1.00 | | | 1.00 | | | 1.00 | | | 1.00 | | |
| Scotland | 0.99 | 0.95 | 1.04 | 1.02 | 0.98 | 1.07 | 1.00 | 0.95 | 1.04 | 1.04 | 0.98 | 1.09 |
| Northern Ireland | 0.95 | 0.91 | 1.00 | 0.93 | 0.89 | 0.97 | 0.90 | 0.85 | 0.95 | 0.86 | 0.81 | 0.91 |
| Wales | 1.04 | 0.99 | 1.09 | 1.01 | 0.96 | 1.06 | 1.00 | 0.95 | 1.05 | 1.06 | 1.00 | 1.12 |
| Region † | | | | | | | | | | | | |
| North East | 0.99 | 0.99 | 0.99 | 1.02 | 0.95 | 1.09 | 1.01 | 0.92 | 1.09 | 1.07 | 0.98 | 1.16 |
| North West | 1.03 | 1.03 | 1.03 | 1.05 | 0.99 | 1.10 | 1.04 | 0.98 | 1.10 | 1.08 | 1.01 | 1.14 |
| Yorksh. & Humb. | 1.00 | 1.00 | 1.00 | 1.00 | 0.94 | 1.05 | 1.01 | 0.95 | 1.08 | 0.97 | 0.90 | 1.03 |
| East Midlands | 1.00 | 1.00 | 1.00 | 1.04 | 0.98 | 1.10 | 1.02 | 0.96 | 1.08 | 0.97 | 0.91 | 1.04 |
| West Midlands | 1.02 | 1.02 | 1.02 | 1.01 | 0.96 | 1.07 | 1.06 | 0.99 | 1.12 | 1.01 | 0.94 | 1.07 |
| East of England | 0.99 | 0.99 | 0.99 | 1.02 | 0.96 | 1.08 | 1.07 | 1.00 | 1.13 | 1.01 | 0.94 | 1.08 |
| London (ref.) | 1.00 | | | 1.00 | | | 1.00 | | | 1.00 | | |
| South East | 1.03 | 1.03 | 1.03 | 1.03 | 0.97 | 1.08 | 1.04 | 0.99 | 1.10 | 1.01 | 0.95 | 1.07 |
| South West | 1.00 | 1.00 | 1.00 | 1.02 | 0.96 | 1.08 | 1.05 | 0.98 | 1.11 | 0.99 | 0.93 | 1.06 |
| Deprivation † | | | | | | | | | | | | |
| ref.) | 1.00 | | | 1.00 | | | 1.00 | | | 1.00 | | |
| Fifth 2 | 1.00 | 0.95 | 1.04 | 1.03 | 0.98 | 1.08 | 0.99 | 0.94 | 1.03 | 0.96 | 0.91 | 1.00 |
| Fifth 3 | 1.05 | 1.00 | 1.10 | 1.04 | 1.00 | 1.09 | 1.05 | 1.00 | 1.09 | 1.00 | 0.96 | 1.05 |
| Fifth 4 | 1.04 | 0.99 | 1.09 | 1.06 | 1.01 | 1.11 | 1.04 | 0.99 | 1.09 | 1.04 | 0.99 | 1.09 |
| Fifth 5 (most deprived) | 1.08 | 1.03 | 1.13 | 1.08 | 1.03 | 1.12 | 1.07 | 1.02 | 1.12 | 1.05 | 1.00 | 1.10 |

95% CI: 95% confidence intervals;

† England only

**Supplement D table 4** **(pg 3) Ratio of self-reported psychological distress symptoms across sociodemographic group, 2009-10 to 2019-20, Usoc**

| Variable | 2017-18 | | | 2018-19 | | | 2019-20 | | |
|---|---|---|---|---|---|---|---|---|---|
| | Ratio | 95% CI | | Ratio | 95% CI | | Ratio | 95% CI | |
| Sex | | | | | | | | | |
| Female | 1.14 | 1.11 | 1.18 | 1.14 | 1.11 | 1.17 | 1.13 | 1.10 | 1.17 |
| Male (ref.) | 1.00 | | | | 1.00 | | | | |
| Age group | | | | | | | | | |
| 16-19 | 1.01 | 0.96 | 1.05 | 1.03 | 0.98 | 1.07 | 0.96 | 0.92 | 1.01 |
| 20-24 | 1.01 | 0.97 | 1.06 | 1.01 | 0.96 | 1.05 | 1.03 | 0.98 | 1.08 |
| 25-29 (ref.) | 1.00 | | | 1.00 | | | 1.00 | | |
| 30-34 | 1.04 | 0.99 | 1.09 | 1.05 | 1.00 | 1.10 | 1.03 | 0.98 | 1.08 |
| 35-39 | 1.01 | 0.96 | 1.06 | 1.01 | 0.97 | 1.06 | 1.00 | 0.96 | 1.05 |
| Cohort | | | | | | | | | |
| 1980-84 (ref.) | 1.00 | | | 1.00 | | | 1.00 | | |
| 1985-89 | 1.02 | 0.97 | 1.07 | 1.03 | 0.98 | 1.08 | 1.03 | 0.98 | 1.08 |
| 1990-94 | 0.97 | 0.93 | 1.01 | 0.98 | 0.94 | 1.02 | 1.00 | 0.95 | 1.04 |
| 1995-99 | 0.99 | 0.95 | 1.03 | 0.99 | 0.95 | 1.04 | 1.03 | 0.98 | 1.08 |
| 2000-03 | 0.98 | 0.93 | 1.03 | 1.01 | 0.97 | 1.06 | 0.96 | 0.92 | 1.01 |
| Ethnicity | | | | | | | | | |
| Asian | 0.88 | 0.83 | 0.92 | 0.89 | 0.85 | 0.93 | 0.92 | 0.88 | 0.97 |
| Black | 0.86 | 0.77 | 0.94 | 0.87 | 0.79 | 0.95 | 0.90 | 0.80 | 1.00 |
| Mixed | 1.05 | 0.95 | 1.14 | 1.03 | 0.92 | 1.13 | 1.12 | 1.01 | 1.23 |
| Other | 1.09 | 0.88 | 1.31 | 1.05 | 0.85 | 1.25 | 0.85 | 0.69 | 1.02 |
| White (ref.) | 1.00 | | | 1.00 | | | 1.00 | | |
| Country | | | | | | | | | |
| England (ref.) | 1.00 | | | 1.00 | | | 1.00 | | |
| Scotland | 1.00 | 0.95 | 1.05 | 0.98 | 0.93 | 1.03 | 0.96 | 0.90 | 1.02 |
| Northern Ireland | 0.87 | 0.82 | 0.93 | 0.92 | 0.86 | 0.99 | 0.92 | 0.86 | 0.97 |
| Wales | 1.01 | 0.95 | 1.07 | 1.02 | 0.96 | 1.07 | 1.03 | 0.96 | 1.09 |
| Region Ϯ | | | | | | | | | |
| North East | 1.07 | 0.98 | 1.16 | 1.05 | 0.96 | 1.14 | 1.01 | 0.92 | 1.10 |
| North West | 1.09 | 1.01 | 1.16 | 1.04 | 0.98 | 1.11 | 1.02 | 0.95 | 1.08 |
| Yorksh. & Humb. | 1.00 | 0.93 | 1.07 | 1.00 | 0.93 | 1.08 | 1.01 | 0.94 | 1.08 |
| East Midlands | 0.94 | 0.88 | 1.00 | 0.96 | 0.90 | 1.03 | 0.99 | 0.93 | 1.06 |
| West Midlands | 0.97 | 0.90 | 1.04 | 1.00 | 0.92 | 1.07 | 1.01 | 0.94 | 1.08 |
| East of England | 0.97 | 0.90 | 1.04 | 1.00 | 0.93 | 1.07 | 0.98 | 0.91 | 1.05 |
| London (ref.) | 1.00 | | | 1.00 | | | 1.00 | | |
| South East | 1.02 | 0.95 | 1.08 | 1.05 | 0.98 | 1.12 | 1.04 | 0.97 | 1.11 |
| South West | 1.03 | 0.95 | 1.10 | 0.99 | 0.92 | 1.06 | 1.01 | 0.94 | 1.08 |
| Deprivation Ϯ | | | | | | | | | |
| ref.) | 1.00 | | | 1.00 | | | 1.00 | | |
| Fifth 2 | 1.01 | 0.96 | 1.05 | 1.01 | 0.96 | 1.05 | 1.01 | 0.96 | 1.06 |
| Fifth 3 | 1.05 | 1.00 | 1.11 | 1.00 | 0.95 | 1.05 | 1.05 | 1.00 | 1.11 |
| Fifth 4 | 1.05 | 1.00 | 1.10 | 1.05 | 0.99 | 1.10 | 1.06 | 1.00 | 1.11 |
| Fifth 5 (most deprived) | 1.05 | 1.00 | 1.11 | 1.04 | 0.99 | 1.10 | 1.05 | 1.00 | 1.11 |

95% CI: 95% confidence intervals;

Ϯ England only

**Supplement D table 5** **Weighted proportion exceeding psychological distress symptom cut-off (14 or over), 2009-10 to 2019-20, Usoc**

| Variable | 2009-10 | | | 2010-11 | | | 2011-12 | | | 2012-13 | | |
|---|---|---|---|---|---|---|---|---|---|---|---|---|
| | Prop. | 95% CI | | Prop. | 95% CI | | Prop. | 95% CI | | Prop. | 95% CI | |
| Overall | | | | | | | | | | | | |
| Case (14 or over) | 0.208 | 0.198 | 0.218 | 0.210 | 0.201 | 0.220 | 0.236 | 0.225 | 0.246 | 0.232 | 0.222 | 0.243 |
| Sex | | | | | | | | | | | | |
| Female | 0.241 | 0.228 | 0.254 | 0.256 | 0.242 | 0.270 | 0.288 | 0.273 | 0.302 | 0.290 | 0.276 | 0.305 |
| Male | 0.176 | 0.162 | 0.190 | 0.167 | 0.154 | 0.180 | 0.186 | 0.172 | 0.200 | 0.178 | 0.164 | 0.191 |
| Age group | | | | | | | | | | | | |
| 16-19 | 0.167 | 0.150 | 0.183 | 0.190 | 0.174 | 0.207 | 0.212 | 0.194 | 0.230 | 0.231 | 0.212 | 0.250 |
| 20-24 | 0.238 | 0.220 | 0.256 | 0.216 | 0.199 | 0.233 | 0.251 | 0.231 | 0.271 | 0.228 | 0.209 | 0.248 |
| 25-29 | 0.211 | 0.195 | 0.228 | 0.220 | 0.204 | 0.236 | 0.241 | 0.223 | 0.260 | 0.238 | 0.218 | 0.258 |
| 30-34 | 0.165 | 0.108 | 0.222 | 0.221 | 0.186 | 0.256 | 0.239 | 0.212 | 0.266 | 0.233 | 0.210 | 0.256 |
| 35-39 | - | - | - | - | - | - | - | - | - | - | - | - |
| Cohort | | | | | | | | | | | | |
| 1980-84 | 0.209 | 0.193 | 0.225 | 0.222 | 0.205 | 0.238 | 0.239 | 0.221 | 0.257 | 0.229 | 0.210 | 0.247 |
| 1985-89 | 0.236 | 0.219 | 0.254 | 0.218 | 0.201 | 0.236 | 0.250 | 0.231 | 0.269 | 0.246 | 0.225 | 0.266 |
| 1990-94 | 0.170 | 0.153 | 0.187 | 0.193 | 0.178 | 0.209 | 0.222 | 0.205 | 0.240 | 0.224 | 0.206 | 0.242 |
| 1995-99 | - | - | - | 0.169 | 0.109 | 0.230 | 0.221 | 0.186 | 0.257 | 0.233 | 0.206 | 0.260 |
| 2000-03 | - | - | - | - | - | - | - | - | - | - | - | - |
| Ethnicity | | | | | | | | | | | | |
| Asian | 0.196 | 0.171 | 0.221 | 0.206 | 0.174 | 0.239 | 0.220 | 0.188 | 0.252 | 0.235 | 0.202 | 0.269 |
| Black | 0.200 | 0.159 | 0.240 | 0.242 | 0.187 | 0.297 | 0.269 | 0.223 | 0.315 | 0.232 | 0.187 | 0.277 |
| Mixed | 0.285 | 0.222 | 0.348 | 0.270 | 0.207 | 0.333 | 0.278 | 0.216 | 0.340 | 0.267 | 0.207 | 0.328 |
| Other | 0.235 | 0.151 | 0.319 | 0.266 | 0.181 | 0.351 | 0.225 | 0.139 | 0.311 | 0.195 | 0.116 | 0.274 |
| White | 0.207 | 0.196 | 0.218 | 0.207 | 0.197 | 0.217 | 0.235 | 0.224 | 0.246 | 0.232 | 0.220 | 0.243 |
| Country | | | | | | | | | | | | |
| England | 0.209 | 0.199 | 0.220 | 0.210 | 0.200 | 0.221 | 0.238 | 0.227 | 0.250 | 0.236 | 0.224 | 0.248 |
| Scotland | 0.205 | 0.161 | 0.248 | 0.230 | 0.197 | 0.263 | 0.240 | 0.207 | 0.274 | 0.257 | 0.223 | 0.292 |
| Northern Ireland | 0.195 | 0.162 | 0.228 | 0.204 | 0.169 | 0.238 | 0.218 | 0.186 | 0.250 | 0.202 | 0.170 | 0.234 |
| Wales | 0.201 | 0.152 | 0.249 | 0.189 | 0.156 | 0.221 | 0.195 | 0.159 | 0.230 | 0.173 | 0.138 | 0.209 |
| Region † | | | | | | | | | | | | |
| North East | 0.177 | 0.140 | 0.214 | 0.219 | 0.172 | 0.265 | 0.195 | 0.154 | 0.236 | 0.257 | 0.195 | 0.319 |
| North West | 0.211 | 0.184 | 0.237 | 0.207 | 0.181 | 0.233 | 0.243 | 0.211 | 0.276 | 0.242 | 0.211 | 0.274 |
| Yorksh. & Humb. | 0.172 | 0.141 | 0.202 | 0.214 | 0.177 | 0.250 | 0.246 | 0.210 | 0.282 | 0.235 | 0.202 | 0.268 |
| East Midlands | 0.224 | 0.187 | 0.260 | 0.196 | 0.164 | 0.227 | 0.227 | 0.194 | 0.261 | 0.240 | 0.205 | 0.275 |
| West Midlands | 0.232 | 0.199 | 0.266 | 0.218 | 0.185 | 0.251 | 0.242 | 0.203 | 0.281 | 0.223 | 0.188 | 0.259 |
| East of England | 0.222 | 0.188 | 0.256 | 0.212 | 0.177 | 0.246 | 0.222 | 0.187 | 0.257 | 0.260 | 0.226 | 0.294 |
| London (ref.) | 0.205 | 0.178 | 0.232 | 0.223 | 0.194 | 0.251 | 0.236 | 0.205 | 0.266 | 0.217 | 0.187 | 0.248 |
| South East | 0.210 | 0.182 | 0.239 | 0.212 | 0.186 | 0.239 | 0.265 | 0.234 | 0.295 | 0.228 | 0.198 | 0.258 |
| South West | 0.224 | 0.187 | 0.261 | 0.186 | 0.154 | 0.218 | 0.233 | 0.201 | 0.265 | 0.241 | 0.200 | 0.283 |
| Deprivation † | | | | | | | | | | | | |
| Fifth 1 (least deprived) | 0.202 | 0.181 | 0.224 | 0.246 | 0.223 | 0.269 | 0.276 | 0.251 | 0.300 | 0.205 | 0.177 | 0.233 |
| Fifth 2 | 0.226 | 0.202 | 0.249 | 0.216 | 0.193 | 0.238 | 0.225 | 0.200 | 0.250 | 0.248 | 0.221 | 0.275 |
| Fifth 3 | 0.214 | 0.188 | 0.241 | 0.211 | 0.187 | 0.234 | 0.233 | 0.207 | 0.258 | 0.235 | 0.209 | 0.261 |
| Fifth 4 | 0.199 | 0.173 | 0.225 | 0.177 | 0.153 | 0.201 | 0.238 | 0.210 | 0.266 | 0.228 | 0.203 | 0.252 |
| Fifth 5 (most deprived) | 0.203 | 0.174 | 0.232 | 0.187 | 0.162 | 0.212 | 0.210 | 0.182 | 0.238 | 0.253 | 0.229 | 0.276 |

Prop: Proportion (weighted) 95% CI: 95% confidence intervals;
† England only

**Supplement D table 5** **(pg 2) Weighted proportion exceeding psychological distress symptom cut-off (14 or over), 2009-10 to 2019-20, Usoc**

| Variable | 2013-14 | | | 2014-15 | | | 2015-16 | | | 2016-17 | | |
|---|---|---|---|---|---|---|---|---|---|---|---|---|
| | Prop. | 95% CI | | Prop. | 95% CI | | Prop. | 95% CI | | Prop. | 95% CI | |
| Overall | | | | | | | | | | | | |
| Case (14 or over) | 0.240 | 0.230 | 0.250 | 0.225 | 0.214 | 0.236 | 0.239 | 0.228 | 0.250 | 0.249 | 0.237 | 0.260 |
| Sex | | | | | | | | | | | | |
| Female | 0.289 | 0.274 | 0.303 | 0.269 | 0.254 | 0.284 | 0.284 | 0.269 | 0.298 | 0.300 | 0.284 | 0.317 |
| Male | 0.193 | 0.179 | 0.207 | 0.182 | 0.168 | 0.196 | 0.194 | 0.179 | 0.210 | 0.196 | 0.180 | 0.211 |
| Age group | | | | | | | | | | | | |
| 16-19 | 0.242 | 0.222 | 0.262 | 0.225 | 0.204 | 0.245 | 0.240 | 0.218 | 0.261 | 0.237 | 0.214 | 0.261 |
| 20-24 | 0.237 | 0.218 | 0.256 | 0.225 | 0.206 | 0.245 | 0.241 | 0.220 | 0.262 | 0.250 | 0.228 | 0.273 |
| 25-29 | 0.249 | 0.227 | 0.271 | 0.236 | 0.214 | 0.259 | 0.237 | 0.214 | 0.260 | 0.249 | 0.225 | 0.273 |
| 30-34 | 0.231 | 0.210 | 0.252 | 0.214 | 0.195 | 0.234 | 0.235 | 0.214 | 0.257 | 0.263 | 0.239 | 0.286 |
| 35-39 | - | - | - | 0.233 | 0.152 | 0.314 | 0.251 | 0.208 | 0.293 | 0.237 | 0.205 | 0.269 |
| Cohort | | | | | | | | | | | | |
| 1980-84 | 0.235 | 0.216 | 0.254 | 0.218 | 0.198 | 0.237 | 0.248 | 0.226 | 0.269 | 0.258 | 0.236 | 0.281 |
| 1985-89 | 0.241 | 0.219 | 0.263 | 0.234 | 0.212 | 0.256 | 0.223 | 0.201 | 0.245 | 0.257 | 0.232 | 0.281 |
| 1990-94 | 0.239 | 0.220 | 0.257 | 0.227 | 0.208 | 0.247 | 0.238 | 0.216 | 0.259 | 0.240 | 0.218 | 0.262 |
| 1995-99 | 0.247 | 0.224 | 0.271 | 0.222 | 0.201 | 0.243 | 0.242 | 0.222 | 0.262 | 0.244 | 0.222 | 0.265 |
| 2000-03 | - | - | - | - | - | - | 0.302 | 0.203 | 0.401 | 0.233 | 0.186 | 0.280 |
| Ethnicity | | | | | | | | | | | | |
| Asian | 0.244 | 0.213 | 0.276 | 0.181 | 0.150 | 0.212 | 0.183 | 0.154 | 0.212 | 0.186 | 0.158 | 0.214 |
| Black | 0.220 | 0.172 | 0.267 | 0.200 | 0.153 | 0.247 | 0.192 | 0.142 | 0.243 | 0.234 | 0.179 | 0.290 |
| Mixed | 0.317 | 0.252 | 0.381 | 0.295 | 0.233 | 0.358 | 0.298 | 0.231 | 0.365 | 0.329 | 0.259 | 0.398 |
| Other | 0.275 | 0.186 | 0.365 | 0.265 | 0.162 | 0.368 | 0.205 | 0.124 | 0.287 | 0.203 | 0.104 | 0.302 |
| White | 0.238 | 0.226 | 0.249 | 0.227 | 0.215 | 0.239 | 0.244 | 0.232 | 0.256 | 0.253 | 0.240 | 0.265 |
| Country | | | | | | | | | | | | |
| England | 0.241 | 0.229 | 0.252 | 0.226 | 0.215 | 0.238 | 0.242 | 0.230 | 0.254 | 0.248 | 0.236 | 0.261 |
| Scotland | 0.244 | 0.206 | 0.282 | 0.227 | 0.190 | 0.265 | 0.239 | 0.200 | 0.278 | 0.280 | 0.234 | 0.325 |
| Northern Ireland | 0.234 | 0.196 | 0.271 | 0.234 | 0.196 | 0.273 | 0.240 | 0.202 | 0.277 | 0.271 | 0.226 | 0.315 |
| Wales | 0.221 | 0.183 | 0.259 | 0.157 | 0.124 | 0.189 | 0.158 | 0.123 | 0.193 | 0.167 | 0.130 | 0.204 |
| Region ͳ | | | | | | | | | | | | |
| North East | 0.234 | 0.182 | 0.285 | 0.208 | 0.158 | 0.257 | 0.224 | 0.171 | 0.278 | 0.265 | 0.203 | 0.328 |
| North West | 0.250 | 0.216 | 0.284 | 0.244 | 0.208 | 0.280 | 0.241 | 0.202 | 0.281 | 0.256 | 0.217 | 0.295 |
| Yorksh. & Humb. | 0.240 | 0.206 | 0.274 | 0.228 | 0.194 | 0.262 | 0.206 | 0.175 | 0.238 | 0.237 | 0.201 | 0.273 |
| East Midlands | 0.238 | 0.205 | 0.271 | 0.241 | 0.204 | 0.277 | 0.247 | 0.206 | 0.288 | 0.258 | 0.219 | 0.297 |
| West Midlands | 0.255 | 0.219 | 0.291 | 0.213 | 0.182 | 0.245 | 0.265 | 0.228 | 0.302 | 0.252 | 0.216 | 0.288 |
| East of England | 0.232 | 0.196 | 0.268 | 0.240 | 0.198 | 0.281 | 0.279 | 0.241 | 0.317 | 0.263 | 0.221 | 0.304 |
| London (ref.) | 0.215 | 0.185 | 0.245 | 0.222 | 0.193 | 0.252 | 0.215 | 0.183 | 0.247 | 0.209 | 0.179 | 0.239 |
| South East | 0.268 | 0.239 | 0.297 | 0.231 | 0.200 | 0.262 | 0.248 | 0.216 | 0.280 | 0.249 | 0.219 | 0.279 |
| South West | 0.233 | 0.196 | 0.269 | 0.195 | 0.163 | 0.228 | 0.255 | 0.220 | 0.290 | 0.273 | 0.232 | 0.313 |
| Deprivation ͳ | | | | | | | | | | | | |
| Fifth 1 (least deprived) | 0.211 | 0.184 | 0.237 | 0.201 | 0.173 | 0.229 | 0.216 | 0.189 | 0.244 | 0.230 | 0.202 | 0.258 |
| Fifth 2 | 0.222 | 0.198 | 0.246 | 0.228 | 0.202 | 0.254 | 0.209 | 0.181 | 0.237 | 0.207 | 0.181 | 0.233 |
| Fifth 3 | 0.248 | 0.221 | 0.275 | 0.207 | 0.180 | 0.235 | 0.250 | 0.223 | 0.278 | 0.237 | 0.209 | 0.264 |
| Fifth 4 | 0.242 | 0.217 | 0.268 | 0.231 | 0.205 | 0.257 | 0.243 | 0.217 | 0.268 | 0.274 | 0.246 | 0.301 |
| Fifth 5 (most deprived) | 0.272 | 0.247 | 0.297 | 0.255 | 0.230 | 0.280 | 0.278 | 0.252 | 0.304 | 0.280 | 0.253 | 0.308 |

Prop: Proportion (weighted) 95% CI: 95% confidence intervals;

ͳ England only

**Supplement D table 5** **(pg 3) Weighted proportion exceeding psychological distress symptom cut-off (14 or over), 2009-10 to 2019-20, Usoc**

| Variable | 2017-18 | | | 2018-19 | | | 2019-20 | | |
|---|---|---|---|---|---|---|---|---|---|
| | Prop. | 95% CI | | Prop. | 95% CI | | Prop. | 95% CI | |
| Overall | | | | | | | | | |
| Case (14 or over) | 0.282 | 0.269 | 0.294 | 0.289 | 0.277 | 0.302 | 0.300 | 0.287 | 0.313 |
| Sex | | | | | | | | | |
| Female | 0.335 | 0.318 | 0.352 | 0.340 | 0.323 | 0.357 | 0.351 | 0.334 | 0.369 |
| Male | 0.226 | 0.209 | 0.243 | 0.236 | 0.218 | 0.254 | 0.244 | 0.226 | 0.262 |
| Age group | | | | | | | | | |
| 16-19 | 0.310 | 0.284 | 0.337 | 0.329 | 0.300 | 0.357 | 0.287 | 0.258 | 0.316 |
| 20-24 | 0.285 | 0.260 | 0.310 | 0.283 | 0.257 | 0.309 | 0.319 | 0.291 | 0.348 |
| 25-29 | 0.268 | 0.243 | 0.294 | 0.261 | 0.235 | 0.287 | 0.292 | 0.263 | 0.321 |
| 30-34 | 0.281 | 0.254 | 0.307 | 0.298 | 0.269 | 0.328 | 0.297 | 0.267 | 0.327 |
| 35-39 | 0.256 | 0.225 | 0.287 | 0.275 | 0.248 | 0.302 | 0.299 | 0.271 | 0.327 |
| Cohort | | | | | | | | | |
| 1980-84 | 0.269 | 0.245 | 0.294 | 0.276 | 0.251 | 0.300 | 0.298 | 0.272 | 0.325 |
| 1985-89 | 0.285 | 0.256 | 0.315 | 0.291 | 0.262 | 0.321 | 0.298 | 0.268 | 0.328 |
| 1990-94 | 0.259 | 0.235 | 0.283 | 0.262 | 0.236 | 0.288 | 0.292 | 0.263 | 0.321 |
| 1995-99 | 0.299 | 0.274 | 0.324 | 0.304 | 0.278 | 0.331 | 0.319 | 0.292 | 0.347 |
| 2000-03 | 0.313 | 0.276 | 0.349 | 0.323 | 0.290 | 0.356 | 0.289 | 0.260 | 0.318 |
| Ethnicity | | | | | | | | | |
| Asian | 0.201 | 0.170 | 0.233 | 0.239 | 0.205 | 0.272 | 0.232 | 0.197 | 0.267 |
| Black | 0.277 | 0.214 | 0.340 | 0.237 | 0.181 | 0.293 | 0.266 | 0.201 | 0.332 |
| Mixed | 0.322 | 0.251 | 0.393 | 0.357 | 0.280 | 0.433 | 0.356 | 0.284 | 0.427 |
| Other | 0.239 | 0.101 | 0.377 | 0.230 | 0.111 | 0.348 | 0.265 | 0.114 | 0.417 |
| White | 0.288 | 0.274 | 0.302 | 0.294 | 0.280 | 0.309 | 0.307 | 0.292 | 0.321 |
| Country | | | | | | | | | |
| England | 0.284 | 0.270 | 0.298 | 0.291 | 0.277 | 0.305 | 0.303 | 0.288 | 0.318 |
| Scotland | 0.276 | 0.241 | 0.311 | 0.299 | 0.247 | 0.351 | 0.314 | 0.267 | 0.362 |
| Northern Ireland | 0.292 | 0.248 | 0.336 | 0.286 | 0.242 | 0.329 | 0.284 | 0.236 | 0.333 |
| Wales | 0.222 | 0.177 | 0.267 | 0.241 | 0.193 | 0.288 | 0.242 | 0.198 | 0.285 |
| Region † | | | | | | | | | |
| North East | 0.306 | 0.240 | 0.372 | 0.317 | 0.251 | 0.382 | 0.306 | 0.243 | 0.370 |
| North West | 0.289 | 0.251 | 0.327 | 0.283 | 0.246 | 0.321 | 0.306 | 0.265 | 0.346 |
| Yorksh. & Humb. | 0.288 | 0.242 | 0.333 | 0.296 | 0.253 | 0.338 | 0.314 | 0.271 | 0.356 |
| East Midlands | 0.252 | 0.209 | 0.294 | 0.265 | 0.222 | 0.308 | 0.297 | 0.253 | 0.341 |
| West Midlands | 0.270 | 0.231 | 0.308 | 0.283 | 0.243 | 0.324 | 0.303 | 0.257 | 0.348 |
| East of England | 0.276 | 0.236 | 0.317 | 0.286 | 0.246 | 0.326 | 0.283 | 0.239 | 0.327 |
| London (ref.) | 0.265 | 0.228 | 0.302 | 0.273 | 0.236 | 0.310 | 0.289 | 0.249 | 0.328 |
| South East | 0.316 | 0.278 | 0.355 | 0.330 | 0.291 | 0.370 | 0.310 | 0.274 | 0.346 |
| South West | 0.294 | 0.254 | 0.334 | 0.287 | 0.246 | 0.327 | 0.330 | 0.282 | 0.378 |
| Deprivation † | | | | | | | | | |
| Fifth 1 (least deprived) | 0.267 | 0.235 | 0.300 | 0.277 | 0.246 | 0.307 | 0.276 | 0.243 | 0.309 |
| Fifth 2 | 0.272 | 0.241 | 0.303 | 0.284 | 0.252 | 0.317 | 0.291 | 0.259 | 0.323 |
| Fifth 3 | 0.273 | 0.243 | 0.303 | 0.267 | 0.238 | 0.297 | 0.297 | 0.265 | 0.329 |
| Fifth 4 | 0.300 | 0.270 | 0.329 | 0.310 | 0.280 | 0.341 | 0.332 | 0.298 | 0.366 |
| Fifth 5 (most deprived) | 0.302 | 0.271 | 0.333 | 0.311 | 0.281 | 0.341 | 0.312 | 0.279 | 0.346 |

Prop: Proportion (weighted) 95% CI: 95% confidence intervals;
† England only
